# Dutch Pharmacogenetics Working Group (DPWG) guideline for the gene–drug interaction of *DPYD* and fluoropyrimidines

**DOI:** 10.1038/s41431-019-0540-0

**Published:** 2019-11-19

**Authors:** Carin A. T. C. Lunenburg, Cathelijne H. van der Wouden, Marga Nijenhuis, Mandy H. Crommentuijn-van Rhenen, Nienke J. de Boer-Veger, Anne Marie Buunk, Elisa J. F. Houwink, Hans Mulder, Gerard A. Rongen, Ron H. N. van Schaik, Jan van der Weide, Bob Wilffert, Vera H. M. Deneer, Jesse J. Swen, Henk-Jan Guchelaar

**Affiliations:** 1 Department of Medical Oncology, Leiden University Medical Centre, Leiden, The Netherlands; 2 Department of Clinical Pharmacy & Toxicology, Leiden University Medical Centre, Leiden, The Netherlands; 3 Royal Dutch Pharmacists Association (KNMP), The Hague, The Netherlands; 4 Pharmacy Boterdiep, Groningen, The Netherlands; 5 Pharmacy De Katwijkse Apotheek, Katwijk, The Netherlands; 6 Department of Public Health and Primary Care (PHEG), Leiden University Medical Center, Leiden, The Netherlands; 7 Department of Clinical Pharmacy, Wilhelmina Hospital, Assen, The Netherlands; 8 Department of Internal Medicine, Radboud University Medical Centre, Nijmegen, The Netherlands; 9 Department of Pharmacology and Toxicology, Radboud University Medical Centre, Nijmegen, The Netherlands; 10 Department of Clinical Chemistry, Erasmus University Medical Center, Rotterdam, The Netherlands; 11 Department of Clinical Chemistry, St. Jansdal Hospital, Harderwijk, The Netherlands; 12 Groningen Research Institute of Pharmacy, Department of PharmacoTherapy, -Epidemiology and -Economics, University of Groningen, Groningen, The Netherlands; 13 Department of Clinical Pharmacy and Pharmacology, University of Groningen, University Medical Center Groningen, Groningen, The Netherlands; 14 Department of Clinical Pharmacy, University Medical Centre Utrecht, Utrecht, The Netherlands

**Keywords:** Prognostic markers, Chemotherapy, Haplotypes

## Abstract

Despite advances in the field of pharmacogenetics (PGx), clinical acceptance has remained limited. The Dutch Pharmacogenetics Working Group (DPWG) aims to facilitate PGx implementation by developing evidence-based pharmacogenetics guidelines to optimize pharmacotherapy. This guideline describes the starting dose optimization of three anti-cancer drugs (fluoropyrimidines: 5-fluorouracil, capecitabine and tegafur) to decrease the risk of severe, potentially fatal, toxicity (such as diarrhoea, hand-foot syndrome, mucositis or myelosuppression). Dihydropyrimidine dehydrogenase (DPD, encoded by the *DPYD* gene) enzyme deficiency increases risk of fluoropyrimidine-induced toxicity. The *DPYD-*gene activity score, determined by four *DPYD* variants, predicts DPD activity and can be used to optimize an individual’s starting dose. The gene activity score ranges from 0 (no DPD activity) to 2 (normal DPD activity). In case it is not possible to calculate the gene activity score based on *DPYD* genotype, we recommend to determine the DPD activity and adjust the initial dose based on available data. For patients initiating 5-fluorouracil or capecitabine: subjects with a gene activity score of 0 are recommended to avoid systemic and cutaneous 5-fluorouracil or capecitabine; subjects with a gene activity score of 1 or 1.5 are recommended to initiate therapy with 50% the standard dose of 5-fluorouracil or capecitabine. For subjects initiating tegafur: subjects with a gene activity score of 0, 1 or 1.5 are recommended to avoid tegafur. Subjects with a gene activity score of 2 (reference) should receive a standard dose. Based on the DPWG clinical implication score, *DPYD* genotyping is considered “essential”, therefore directing *DPYD* testing prior to initiating fluoropyrimidines.

## Introduction

The role of heritable genetic variation on drug response is referred to as pharmacogenetics (PGx). Germline mutations in pharmacogenetic loci can predict phenotypic differences in drug response and can be used to guide dose and drug selection to achieve safer and more (cost-) effective pharmacotherapy. PGx guided pharmacotherapy is one of the first clinical applications of genomics in medicine. Despite scientific and clinical advances in the field of PGx, clinical adoption has remained limited. Barriers preventing implementation have been previously reported [1]. Some of these barriers have been overcome in the past years. One of these barriers was the lack of clear guidelines on how to interpret and apply PGx test results.

The Royal Dutch Pharmacists Association (KNMP) established the Dutch Pharmacogenetics Working Group (DPWG) in 2005 to overcome this barrier [2]. The main objectives of the DPWG are (1) to develop PGx informed therapeutic recommendations based on systematic literature review, and (2) to assist physicians and pharmacists by integrating the recommendations into computerized systems for drug prescription, dispensing and automated medication surveillance. This manuscript thus provides both the content required for enabling local translation of assay results into the predicted phenotype (in this case the gene activity score) and for programming therapeutic recommendations into local clinical decision support systems. With the objective of implementing PGx into routine care, the DPWG has additionally developed the clinical implication score, which is given to every gene–drug interaction. The aim of this score is to direct clinicians on whether or not to order relevant PGx genotyping tests before initiating therapy. Recently, the DPWG guidelines were endorsed by the European Association of Clinical Pharmacology and Therapeutics and the European Association of Hospital Pharmacists [3, 4]. Other initiatives such as the Clinical Pharmacogenetics Implementation Consortium (CPIC) were also established to support clinical implementation [5, 6].

The DPWG is a multidisciplinary group in which (clinical) pharmacists, physicians, clinical pharmacologists, clinical chemists and epidemiologists are represented. From 2005 onwards, the DPWG has systematically executed 90 risk analyses for potential gene–drug interactions resulting in 49 guidelines providing therapeutic recommendations for one or more aberrant phenotypes [7]. Available DPWG guidelines and future updates will be published in an effort to provide transparency of their development and to fulfil the public demand for their publication.

This guideline describes the starting dose optimization of three anti-cancer drugs (fluoropyrimidines: 5-fluorouracil, capecitabine and tegafur) to decrease the risk of severe, potentially fatal, toxicity; such as diarrhoea, hand-foot syndrome, mucositis or myelosuppression. Dihydropyrimidine dehydrogenase enzyme (DPD) deficiency (which is encoded by the *DPYD* gene) increases the risk of fluoropyrimidine-induced toxicity. The gene activity score is currently based on the results of four *DPYD* variants, predicts DPD enzyme activity and is used to optimize an individual’s starting dose. The gene activity score ranges from 0 (no DPD activity) to 2 (normal DPD activity). This manuscript provides an overview of the guideline development and summarizes the pharmacotherapeutic recommendations. In addition, a comparison to alternative guidelines is presented. The “gene–drug interaction” section includes background on the pharmacological mechanism of the interaction. In addition it also includes a list of the *DPYD* variants associated with toxicity and the method developed by DPWG for local translation of assay results into the gene activity score. This information may be useful for laboratories to select and design a *DPYD* genotyping assay and subsequently determine the patients’ predicted phenotype based on the genotype results. Consequently, the literature review supporting the *DPYD-*fluoropyrimidine interaction is described and the DPWG guideline is presented. A summary of all references identified by the systematic review that were subsequently used to develop this guideline, can be found in Supplementary Tables 1 and 2. The recommendations provided in this manuscript can be used in combination with a patients’ predicted phenotype to optimize starting dose of fluoropyrimidines, thereby decreasing the risk of severe and potentially fatal toxicity.

### Drugs: fluoropyrimidines (5-fluorouracil, capecitabine and tegafur with DPD-inhibitors)

Fluoropyrimidines are antimetabolite drugs widely used in the treatment of colorectal, breast, stomach and skin cancer. Each year, over two million patients worldwide receive treatment with fluoropyrimidines. This includes 5-FU and its oral pro-drugs capecitabine and tegafur. Up to 30% of patients experience severe toxicity (Common Terminology Criteria for Adverse Events, CTCAE, Grade ≥3), including diarrhoea, hand-foot syndrome, mucositis and myelosuppression. For ~1% of patients toxicity is fatal [8, 9]. Toxicity may occur within the first treatment cycle (early onset), supporting the importance of optimizing the starting dose of fluoropyrimidine pharmacotherapy on a personalized basis, before initiating therapy [10].

Capecitabine is metabolised into 5-FU in three consecutive steps. Capecitabine is firstly metabolised to 5′-deoxy-5-fluorocytidine (5′-DFCR) by carboxylesterase, subsequently, 5′-DFCR is converted into 5′-deoxy-5-fluorouridine (5′-DFUR) by cytidine deaminase, and to 5-FU by thymidine phosphorylase. 5-FU is metabolised in tissues to 5-fluoro-2′-deoxyuridine and then to 5-fluoro-2′-deoxyuridine-5′-monophosphate, the active metabolite of the drug. The active metabolite inhibits the enzyme thymidylate synthase, resulting in inhibition of DNA synthesis and repair, inducing cell apoptosis and thus, its effect. In addition, toxic effects resulting from partial incorporation of 5-FU and its metabolites in DNA and RNA contribute to the drug’s mechanism of action [11].

Tegafur is metabolised into 5-FU and into the less cytotoxic metabolites 3-hydroxytegafur, 4-hydroxytegafur and dihydrotegafur by CYP2A6. The less toxic metabolites are renally cleared. Tegafur was combined with the DPD inhibitor uracil and is now combined with the DPD inhibitor gimeracil and the orotate phoshoribosyltransferase inhibitor oteracil. Oteracil diminishes the activity of 5-FU in normal gastrointestinal mucosa. The DPD inhibitors diminish the formation of functionally inactive metabolites of 5-FU that contribute to adverse events like stomatitis and mucositis. Both uracil and gimeracil inhibit DPD activity reversibly and have a shorter elimination half-life and thus shorter period of action than tegafur. For this reason, genetic variants influencing DPD enzyme activity are clinically relevant for tegafur in combination with DPD inhibitors.

### Gene: dihydropyrimidine dehydrogenase (*DPYD*)

The *DPYD* gene encodes the enzyme DPD. *DPYD* is located on chromosome 1p21.3, and transcription variant 1 (NM_000110.3) has 26 exons, spanning ~900 kb [12]. Over 160 different allele variants in *DPYD* have been identified and described in literature [13]. According to the gnomAD browser [14], which contains whole exome data of almost 140,000 individuals, *DPYD* contains 2190 known variants. The prevalence of individual variants is low. The effect of genetic variation on DPD enzyme activity is not fully established for the majority of variants and the size of the effect can differ between variants.

The frequency of the various *DPYD* variants and the associated phenotypes appears to vary significantly between nations and ethnic groups. For example, in the Caucasian population, ~3–5% has a partial DPD enzyme deficiency and 0.1–0.2% has a complete DPD enzyme deficiency. On the other hand, ~8% of the African American population has a partial DPD enzyme deficiency [15, 16].

### Gene–drug interaction

#### Pharmacological mechanism

**Fig. 1:**
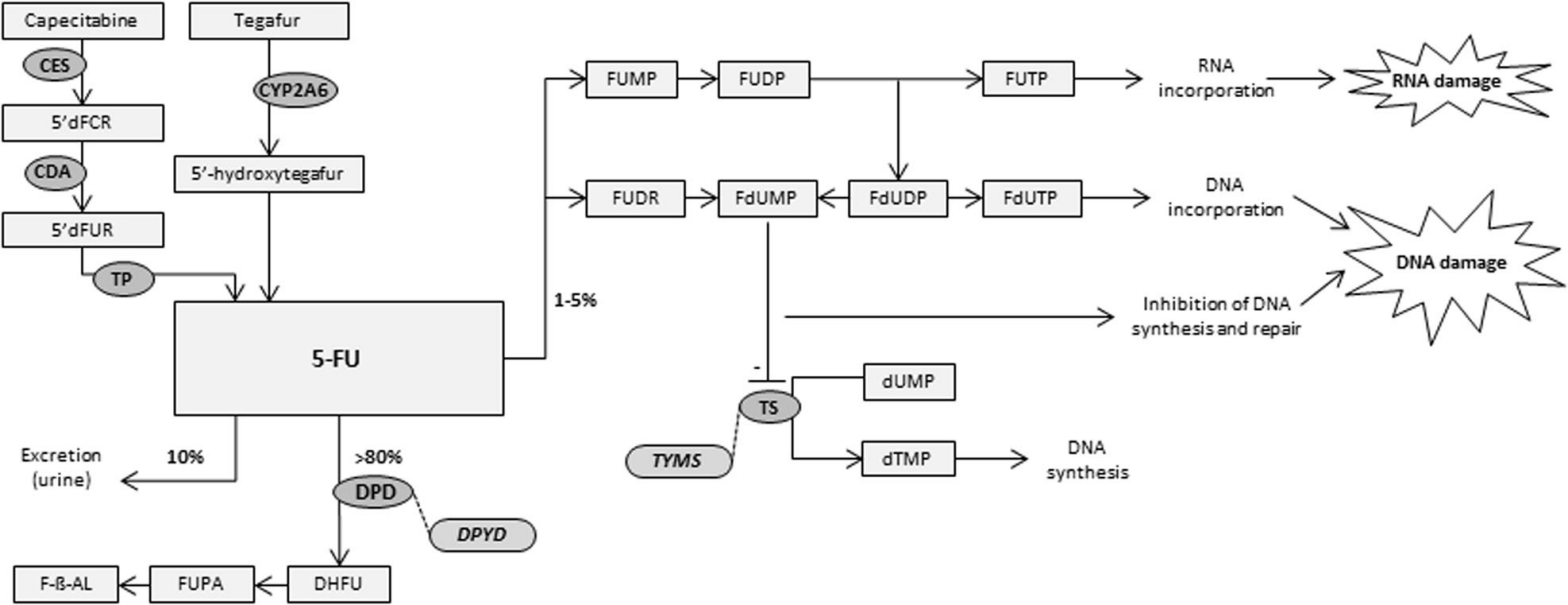
Schematic overview of fluoropyrimidine metabolism. In brief: tegafur, 5FU, capecitabine are metabolised into three major metabolites. FdUMP, which inhibits TS and prevents conversion of dUMP to dTMP, which is necessary for pyrimidine and DNA synthesis. FdUTP is incorporated in DNA, FdUTP is incorporated in RNA, both resulting in cell death. CES carboxylesterase, CDA cytidine deaminase, 5′dFCR 5′-deoxy-5-fluorocytidine, 5′dFUR 5′-deoxy-5-fluorouridine, 5-FU 5-fluorouracil, TP thymidine phosphorylase, *DPYD* gene encoding DPD, DPD dihydropyrimidine dehydrogenase, DHFU 5,6-dihydrofluorouracil, FUPA fluoro-ß-ureidopropionate, F-ß-AL Fluoro-ß-alanine, FUMP fluorouridine monophosphate, FUDP fluorouridine diphosphate, FUTP fluorouridine triphosphate, FUDR fluorodeoxyuridine, FdUMP fluorodeoxyuridine monophosphate, FdUDP fluorodeoxyuridine diphosphate, FdUTP fluorodeoxyuridine triphosphate, dUMP deoxyuridine monophosphate, dTMP deoxythymidine monophosphate, TS thymidylate synthase, *TYMS* gene encoding TS

A schematic overview of fluoropyrimidine metabolism is shown in Fig. 1. The DPD enzyme is mainly found in liver, but also intestinal mucosa, leucocytes, tumour cells and other tissues. Over 80% of 5-FU is inactivated to 5-fluoro-5,6-dihydrouracil (DHFU) by DPD. The decreased metabolic activity of DPD leads to increased intracellular concentrations of active metabolites of 5-FU [17]. The increased intracellular concentration of 5-fluoro-2′-deoxyuridine-5′-monophosphate (FdUMP) increases the risk of toxicity such as diarrhoea, hand-foot syndrome, mucositis and myelosuppression. Variants in the *DPYD* gene can result in reduced or even absent DPD enzyme activity, increasing the risk of severe toxicity. For example, 73% of the patients with DPYD*2A experienced severe toxicity when treated with a full dose, compared with 23% of *1 allele carriers (wild-type patients) who experienced toxicity [18]. Many enzymes are involved in fluoropyrimidine metabolism, however, this guideline is limited to the role of the DPD enzyme in causing toxicity.

Since the genetic variation in *DPYD* only partially determines DPD enzyme activity, these guidelines for dose adjustment based on the predicted phenotype are no more than a tool that can be used to achieve the desired intracellular concentration of the active metabolite, to minimize risk of toxicity. The absence of tested variants does not eliminate the risk of toxicity. Pharmacokinetic dose adjustment (guided by steady-state plasma concentrations or AUC) may also be useful to optimize the dose of 5-FU. This is, however, currently not routinely used for capecitabine and tegafur, as they are mainly converted into 5-FU within tissue.

#### *DPYD* variants associated with toxicity

The variants known or suspected to have an effect on DPD enzyme activity, are listed in Table 1. These variants are mapped by the level of evidence for which association with toxicity has been established (columns) and the variant’s effect on DPD enzyme activity (rows). Novel variants in *DPYD* will continue to be identified with the introduction of NGS techniques to clinical practice. The *DPYD* variants reported in this manuscript are reported on www.PharmGKB.org, which is referred to on https://databases.lovd.nl/shared/genes/*DPYD*. However, in order for these variants to be included in Table 1, sufficient evidence regarding the effect on enzyme function or the onset of toxicity must be investigated, possibly by using the *DPYD-*Varifier [19] or by phenotyping patients who carry a novel variant. An update of this guideline will be published when a renewed recommendation is given following newly published articles.

**Table 1:** Known *DPYD* variants stratified by level of evidence on the association with toxicity and predicted DPD enzyme activity

Level of evidence	Sufficient evidence^a^	Insufficient evidence^b^
DPD enzyme activity		
Fully functional^c^	DPYD*5 = c.1627A>G DPYD*9A = c.85T>C	DPYD*4 = c.1601G>A DPYD*6 = c.2194G>A DPYD*9B = c.[85T>C;2657G>A] DPYD*11 = c.1003 G > T c.496A>G c.1896T>C c.1129-15T>C (IVS10-15T>C)
Reduced functionality^d^	c.2846A>T c.[1236G>A;1129–5923C>G] (hapB3)^e^	
Fully dysfunctional^f^	DPYD*2A = c.1905 + 1G>A DPYD*13 = c.1679T>G	DPYD*3 = c.1898del DPYD*7 = c.299_302del DPYD*8 = c.703C>T DPYD*10 = c.2983G>T DPYD*12 = c.[62 G > A;1156 G > T] c.1651G>A c.300 C>A^g^ c.1024 G>A^g^ c.1025 A>G^g^ c.1475 C>T^g^ c.1774C>T^g^ c.(2058+1_2059-1)_(2299+1_2300-1)dup c.257C>T^g^

The variants in this table were selected based on literature in Supplementary Table 1 and 2. However high allele frequency variants reported only in case reports with fluoropyrimidine toxicity were excluded. For these variants the association with DPD enzyme activity, and resulting severe fluoropyrimidine-induced toxicity, cannot be determined.

^a^DPWG has concluded an association between fully functional variants and no resulting toxicity, and an association between reduced functionality variants or fully dysfunctional variants and association with the onset of severe fluoropyrimidine-induced toxicity

^b^DPWG has concluded there is insufficient evidence to associate a predicted DPD enzyme activity for these variants and the onset of severe fluoropyrimidine-induced toxicity

^c^These variants are not included in the prospective *DPYD* genotyping panel, as there is no effect on predicted DPD enzyme activity, and therefore there is no association with the onset of severe fluoropyrimidine-induced toxicity

^d^The effect of the variant on the protein sequence suggests that the protein may still be partially functional. Therefore residual metabolic DPD capacity may be present

^e^Variant c.1236G>A, which does not lead to an alternative amino acid, is in complete linkage disequilibrium with variant c.1129–5923C>G, which leads to aberrant splicing in mRNA, which leads to a premature stop codon as a result. The resulting DPD enzyme activity is 50% of the normal activity. Both variants are part of haplotype B3

^f^The effect of the variant on the protein sequence suggests that the protein may be fully dysfunctional

^g^These variants have decreased in vitro enzyme activity

Variants from the table according to multiple nomenclatures (HGVS: NM_000110.3, NP_000101.2 and NC_000001.10)

#### Translation of genotype to predicted phenotype

The DPWG has concluded that four variants have sufficient evidence to be implemented into clinical care: DPYD*2A (c.1905+1G>A, IVS14+1G>A), DPYD*13 (c.1679T>G), c.2846A>T and c.1236G>A (in linkage disequilibrium with c.1129–5923C>G). The current guideline only reports recommendations for these four variants; no recommendations are provided for other variants in *DPYD* or other genes. The results of this genotyping panel can be used to predict a patient’s phenotype, i.e. the DPD enzyme activity. This predicted DPD activity can be expressed as the *DPYD-*gene activity score, which ranges from 0 (no or virtually no DPD enzyme activity) to 2 (normal DPD enzyme activity due to homozygosity for fully functional alleles, both assigned an activity score 1). The gene activity score is a sum of the two activities of protein isoforms expressed from both alleles. The development of the gene activity score is published elsewhere [20]. Although the gene activity score 0.5 can be predicted based on genotype, the predicted enzyme activity is not reliable. Therefore the gene activity score 0.5 has been replaced with predict the gene activity score correctly (PHENO). A simplified translation of genotypes to predicted phenotypes as gene activity scores, is presented in Table 2. The included variants are those for which substantial and sufficient evidence on the relation to severe toxicity has been established. It is a limitation to restrict to these four variants, as other variants may influence DPD activity as well. However, not all variants having a possible effect on DPD enzyme activity may have been identified yet or evidence for identified variants is insufficient. Therefore, this may result in the incorrect prediction of the DPD enzyme activity. Another limitation is that currently used genotyping methods are unable to determine the allelic location of the variants, but only the dichotomous presence or absence of the variant. This becomes a limitation when two or more different genetic variants are identified in a patient. In this case, either both genetic variants may be on the same allele, resulting in a genotype with one fully functional allele and one reduced functionality allele, or alternatively, both genetic variants may reside on different alleles, resulting in two alleles with inactive or reduced functionality. The latter is more likely to occur. The total gene activity score, however, differs between these cases. When the DPD enzyme activity cannot be predicted correctly, an additional phenotyping test is required to determine the DPD enzyme activity. The relationship between genotype result and predicted phenotype in patients carrying no variants or one or more variants leading to decreased DPD enzyme activity are shown in Supplementary Table 3. The frequency of individuals carrying two or more of four variants considered in the current guideline is rare, but can be assigned a gene activity score. A complete genotype to predicted phenotype translation table can be found in Supplementary Table 4, which can be used to programme the translation of genotype results into predicted phenotypes in laboratory information systems.

**Table 2:** The translation of genotypes to predicted phenotypes, as gene activity scores

Patient genotype	Gene activity score
Carrier of no variants associated with either reduced functionality or fully dysfunctional DPD activity (*1/*1)	Gene activity score 2
Carrier of one variant associated with reduced functionality of DPD activity (*1/c.1236G>A or *1/c.2846A>T)	Gene activity score 1.5
Carrier of one variant associated with fully dysfunctional DPD activity (*1/*2A or *1/*13)	Gene activity score 1
Carrier of two variants associated with reduced functionality of DPD activity (for example c.1236G>A/c.2846A>T) Or Carrier of one variant associated with reduced functionality of DPD activity and one variant associated with fully dysfunctional DPD activity (combinations of c.2846A>T or c.1236G>A with *2A or *13, example given *2A/c.2846A>T)	PHENO: DPD enzyme activity cannot be predicted correctly, an additional phenotyping test is required to determine the DPD enzyme activity
Carrier of two variants associated with fully dysfunctional DPD activity (*2A/*2A or *13/*13 or *2A/*13)	Gene activity score 0

#### Additional phenotyping test when genotype is unable to predict phenotype

In contrast to the *DPYD* genotyping test, which aims to predict DPD enzyme activity, a DPD phenotyping test can be performed to measure the actual DPD enzyme activity. Possible methods to perform phenotyping are to measure the DPD enzyme activity in peripheral blood mononuclear cells (PBMCs) or to measure the uracil concentrations in plasma or urine [21]. The average Caucasian DPD enzyme activity is 9.9 ± 0.95 nmol/h per mg protein [22]. Less commonly performed methods include: (1) the 2-^13^C-uracil breath test [23], where ^13^C0_2_ is measured, which is a product of 2-C^13^-uracil degradation by DPD and other enzymes involved in the katabolic route of pyrimidines; (2) the quantification of the uracil/dihydrouracil ratio in plasma, where endogenous substrates uracil and dihydrouracil are measured [24, 25], although recently it was shown that uracil levels were superior to the dihydrouracil/uracil ratio as a predictor of severe toxicity [26]; and (3) measurement the metabolism of a single dose of uracil [27]. However, all DPD phenotyping tests have their limitations. Currently, the DPD enzyme activity measurements from PBMCs are considered the best developed DPD phenotyping test in The Netherlands [27, 28].

### Supporting body of evidence

A detailed description of the methods used for literature collection, assessment and preparation of the gene-drug monograph has previously been published elsewhere [2, 7]. In brief, a systematic review of literature was performed and relevant articles were summarized by a scientist of the Royal Dutch Pharmacists Association (MN). The performed search strategy can be found in Supplementary Material 1. Each article was provided with two scores: (1) quality of evidence and (2) clinical impact. The quality of evidence was scored on a five-point scale ranging from 0 (lowest—data on file) to 4 (highest—well performed controlled studies or meta-analysis) and the clinical impact of clinical effect was scored on a seven-point scale ranging from AA^#^ (positive effect) to F (highest negative effect). The criteria used to develop these scores have been published in detail previously [2, 7]. This clinical impact scale (AA^#^-F) runs parallel to the Common Terminology Criteria for Adverse Events (CTCAE); where CTCAE grade 5 severity is equal to clinical relevance score F (death) and CTCAE grade 1 severity is equal to clinical relevance score B. The clinical relevance score additionally includes the scores AA^#^, AA and A, since these do not exist in the CTCAE. These regard “positive clinical effect”, “no clinical or kinetic effect”, and “significant kinetic effect or not clinically relevant effect”, respectively. The summaries of articles, and their respective scores, reviewed to devise this guideline can be found in the Supplementary Tables 1 and 2. The summaries of each article and their respective scores were checked by two independent DPWG members.

For 5-FU/capecitabine, the initial literature search was performed on March 24, 2009, followed by a second and third search on July 9, 2014 and October 19, 2017. To update this guideline to the current date, an additional literature search was performed on January 30, 2019. Case reports concerning systemic 5-FU or capecitabine therapy were excluded in this literature review, due to a large number of case reports and other available publications of greater evidentiary quality. Kinetic studies from 2009 onwards were only included if the kinetic parameters were given per genotype. Clinical studies were only included if the patient numbers exceeded 500 (from 2009 onwards) or 1000 (from May 2014 onwards) and the patient numbers with partially functional activity were at least ten or if the study investigated a variant for which no studies were as yet included or if the study investigated the effect of dose adjustment. From 2009, articles investigating the effect of a group containing both polymorphisms known to increase the risk of toxicity and polymorphisms not known to increase the risk of toxicity were not included. If more than one article described data of the same patient group and the same polymorphisms, only the article with data from the largest amount of patients was included.

For tegafur, the initial literature search was performed on August 20, 2009, followed by a second, third and fourth search on October 2, 2012, July 27, 2015, and October 19, 2017. To update this guideline to current date, an additional literature search was performed on January 30, 2019.

### General conclusion of evidence

In the systematic review performed for 5-FU/capecitabine, 18 of 20 studies and all three meta-analyses found an increased risk of grade ≥3 toxicity (either overall toxicity or at least one specified type of toxicity) for patients carrying variants resulting in reduced DPD enzyme activity (ranging from gene activity score 0 to 1.5, and PHENO). This increased risk was shown separately for patients assigned *DPYD-*gene activity scores 1 and 1.5, but gene activity scores 0 and PHENO were only investigated when grouped with patients assigned other gene activity scores. However, the increased risk of toxicity for patients assigned gene activity scores 0 and PHENO can be concluded based on the confirmed association for gene activity scores 1 and 1.5, where deficiency is less, and is further supported by cases of patients assigned gene activity scores 0 and PHENO who developed severe toxicity. Only one study investigating clinical outcome concluded there was no effect of variants on risk of toxicity. Based on the systematic review, the DPWG concludes that a gene–drug interaction is present and that DPD enzyme deficiency increases risk of severe toxicity in patients using capecitabine/5-FU. The highest quality of evidence concluding a gene–drug interaction was scored 4.

In the systematic review performed for tegafur with the DPD inhibitor uracil, one case report described four patients who used standard doses and developed severe toxicity. These patients were assigned *DPYD*-gene activity scores 1 and 1.5. Toxicity (CTCAE grade 4) was similar to that reported in patients treated with 5-FU or capecitabine, both of which are given without a DPD inhibitor. There were no data available for patients assigned *DPYD-*gene activity score 0 or PHENO, however, the increased risk of toxicity among these patients can be concluded based on the confirmed association with toxicity for gene activity scores 1 and 1.5, where deficiency is less. Based on the systematic review, the DPWG concludes that there is a clinically relevant gene–drug interaction present and that DPD enzyme deficiency increases risk of severe toxicity in patients using tegafur with DPD inhibitors. The highest quality of evidence concluding a gene–drug interaction was scored 2.

### Pharmacotherapeutic recommendations

The DPWG therapeutic recommendation using a patient’s pre-therapeutic PGx test result to optimize starting dose of 5-FU/capecitabine and tegafur with DPD inhibitors is summarized in Supplementary Tables 5 and 6, respectively.

In brief, for patients initiating 5-fluorouracil or capecitabine: subjects with a gene activity score of 0 are recommended to avoid both systemic and cutaneous 5-fluorouracil or capecitabine, alternatively, DPD enzyme activity may be determined to adjust the systemic dose accordingly; subjects with a gene activity score of 1 or 1.5 are recommended to initiate therapy with 50% of the standard dose of 5-fluorouracil or capecitabine. Further titration of the dose is possible, guided by toxicity. If genotype results cannot PHENO, for example due to multiple identified variants, it is advised to determine the DPD enzyme activity to define an initial starting dose. For patients initiating tegafur, a gene activity score of 0, 1 or 1.5 recommends avoiding tegafur; when this is not possible, starting with a low dose and titrating dose based upon toxicity. If genotype results cannot PHENO, for example due to multiple identified variants, it is advised to determine the DPD enzyme activity to define an initial starting dose. A gene activity score of 2 (reference value) does not result in a recommendation for dose adaptation for 5-FU, capecitabine or tegafur.

Where possible, dose adjustments have been calculated based on 5-FU clearance or AUC after administration of 5-FU or capecitabine. Data were also extrapolated to tegafur with DPD inhibitor, as this compound also follows the same catabolic and anabolic routes after conversion to 5-FU after clearance of the DPD inhibitor from the body. Data on 5-FU clearance are only available for patients carrying DPYD*1/*2A, DPYD*1/c.2846A>T and DPYD*2A/c.2846A>T. There are data from one patient with DPYD*1/*13 who developed severe toxicity after 5-FU use, from one patient with c.2846A>T/c.2846A>T and from one patient with c.1236G>A/c.2846A>T.

See Supplementary Tables 7 and 8 for an overview of suggested pop-up texts for electronic prescribing systems for pharmacists and physicians. These can be used to programme alerts into the clinical decision support system (CDSS). The guidelines and background information will be available on PharmGKB.org.

### Implications for clinical practice

There is currently an ongoing debate regarding whether and which single drug–gene pairs should be implemented into routine care. Points of debate include the amount of evidence that is necessary supporting effectiveness of pre-emptive genotyping, the cost-effectiveness of the intervention and reimbursement of PGx testing [29, 30]. This inconclusive debate seems to have hampered implementation of drug–gene pairs which seem ready for implementation [1, 31]. In an effort to overcome this inconclusiveness and to direct clinicians on whether or not to order relevant PGx genotyping tests before initiating therapy, the DPWG has developed the clinical implication score. The pre-emptive PGx results for a certain drug–gene pair can be scored as: essential, beneficial, potentially beneficial or not required. The development of these categories and the systematic scoring criteria are discussed elsewhere [32]. In brief, the implications for clinical practice are based on a list of four criteria regarding the following: the clinical effect associated with the gene–drug interaction, the level of evidence supporting the clinical effect, the effectiveness of the intervention in preventing the clinical effect (which includes the number needed to genotype) and the PGx information included in the drug-label. The scores provided for each of these criteria by the DPWG can be found in Supplementary Table 9.

As a result, the DPWG has concluded the clinical implication score of *DPYD*-fluoropyrimidines to be “essential”. This score dictates that *DPYD* genotyping prior to treatment must be performed for all patients initially being prescribed therapy with 5-FU, capecitabine or tegafur with DPD inhibitors, to optimize the initial dose and to prevent potentially fatal toxicity.

### Differences between available guidelines

Other guidelines regarding the gene–drug interaction of *DPYD* and fluoropyrimidines have been developed. To the best of our knowledge, guidelines are available from CPIC [11, 33], French (French Network of Pharmacogenetics—RNPGx) [34] and Italian (Associazione Italiana di Oncologia Medica—AIOM-SIF) [unpublished guidelines, *edited by the AIOM-SIF Working Group*] initiatives. We have compared the DPWG guidelines with other available guidelines published in English. This regards only the CPIC guideline, since the French and Italian guidelines are unpublished or not in English.

#### CPIC

Differences between CPIC and DPWG methodology, genotype to phenotype conversion and recommendations have previously been described in detail [6]. However, both guidelines have been updated [5, 33, 35, 36]. The current DPWG and CPIC guidelines [5] for *DPYD*/fluoropyrimidines differ regarding the therapeutic recommendations. In contrast to CPIC, DPWG distinguishes between 5-FU/capecitabine and tegafur within the therapeutic recommendations for fluoropyrimidines, where the CPIC guideline does not provide any dosing recommendations for tegafur due to the limited available evidence. DPWG also further distinguishes between systemic and cutaneous routes of administration within the 5-FU/capecitabine recommendations. The therapeutic recommendations for 5-FU/capecitabine also differ regarding the following: (1) For patients with gene activity score 0: DPWG recommends phenotyping while CPIC does not when no alternative is available. (2) For patients with gene activity score PHENO DPWG recommends phenotyping to determine starting dose or selection of an alternative whereas CPIC recommends an alternative or a strongly reduced dose with therapeutic drug monitoring. (3) DPWG recommends to phenotype all homozygous carriers of any variant, whereas CPIC recommends to adjust the dose for a homozygous carrier of c.2846A>T with more than 50%.

## Supplementary information

Literature review of *DPYD*-[5-FU/capecitabine] interactions to support the therapeutic dose guidelines to optimize dose

Literature review of *DPYD*/[tegafur with DPD inhibitor] interactions to support the therapeutic dose guidelines to optimize dose

Relationship between genotype result and predicted phenotype in patients carrying no variants or one or more variants leading to decreased DPD enzyme activity

Genotype to predicted phenotype translation to be programmed into laboratory information system

Dutch Pharmacogenetics Working Group (DPWG) Guideline for *DPYD* and 5-FU/capecitabine

Dutch Pharmacogenetics Working Group (DPWG) Guideline for *DPYD* and tegafur with DPD inhibitors

Suggested clinical decision support texts for various health care professionals for 5-FU/capecitabine

Suggested clinical decision support texts for health care professionals for tegafur with DPD inhibitors

The clinical implication score of *DPYD*-fluoropyrimidines is “essential”, based on the criteria and corresponding scores given by the DPWG

Supplementary Material

## Data Availability

All data and material are either included in the supplementary information or publicly available (i.e. the published articles, PubMed). The guidelines and background information will be available on PharmGKB.org.
