# Supplementary material for: Dutch Pharmacogenetics Working Group (DPWG) guideline for the gene–drug interaction of DPYD and fluoropyrimidines
Source: Eur J Hum Genet. 2019 Nov 19;28(4):508–17. doi: 10.1038/s41431-019-0540-0 (PMC7080718; doi:10.1038/s41431-019-0540-0)
Supplement: Supplementary file 1 — Literature review of DPYD-[5-FU/capecitabine] interactions to support the therapeutic dose guidelines to optimize dose [file 41431_2019_540_MOESM1_ESM.docx]

**Supplementary Table 1:** Literature review of *DPYD*-[5-FU/capecitabine] interactions to support the therapeutic dose guidelines to optimize dose

| **Reference** | **Code** | **Effect** | **Comments** |
| --- | --- | --- | --- |
| ref. 1 – CAP, mono/comb  Kleinjan JP et al.  Tolerance-based capecitabine dose escalation after DPYD genotype-guided dosing in heterozygote DPYD variant carriers: a single center observational study.  Anticancer Drugs 2019 Jan 8  [Epub ahead of print].  PubMed PMID: 30628914.  ref. 1, continuation  ref. 1, continuation  ref. 1, continuation  ref. 1, continuation  ref. 1, continuation  ref. 1, continuation | Level of evidence score: 3  AS 1-1.5 on genotype guided dose: Clinical Relevance Score AA | 11 patients, heterozygous for a gene variant, received capecitabine treatment with reduced doses (75% of the normal dose for gene activity 1.5 and 50% of the normal dose for gene activity 1), that were subsequently adjusted on basis of tolerance according to a prespecified protocol. Only patients receiving capecitabine mono- or combination therapy without radiation and completing at least one treatment cycle were considered. Capecitabine doses according to protocol (normal doses) were 1000-1250 mg/m^2^ twice daily. Therapy was evaluated after 1-2 cycles. Clinically relevant capecitabine-induced toxicities resulted in a dose reduction according to standard practice, whereas no or minimal toxicities allowed for a 15% dose escalation. In the remaining cases, the dose was maintained because of acceptable toxicities. The capecitabine dose could be increased repeatedly on the basis of tolerability, but could never exceed the conventional dose for the intended treatment. As is standard practice, the patients’ clinical condition was also taken into account. Doses were only increased if the patient had a good clinical condition and if oncologist and patient agreed on increasing the dose.  The dose was increased in 6 patients and subsequently reduced again in 2 patients. Of the 6 patients with a dose increase 1 (17%) experienced a grade 3 toxicity. 2 of the 11 patients (18%) developed a grade 3 adverse event on the reduced starting dose. For one patient with grade 3 diarrhoea the dose was reduced because of this, for the other patient with grade 3 neutropenia, the dose was not reduced. The 3 patients maintained on the reduced starting dose because low-grade toxicities and a moderate clinical condition did not allow for a dose increase, developed disease progression. Of the 5 patients who tolerated a dose increase, 2 developed disease progression and a 3^rd^ patient had his colon resected. A median dose increase of 8.5% (range 4-31%) was achieved.  Diarrhoea and haematological toxicities grade ≥ 3 and hand-foot syndrome grade ≥ 2 were considered severe. Hospitalisations were defined as capecitabine treatment related or possibly treatment related hospitalisations. Cycles were noted only if at least 50% of the capecitabine days were completed.  Results were compared with 174 patients without gene variant treated with normal dose capecitabine.  Genotyping:  - 7x gene activity score 1.5 (6x *1/c.1236G>A, 1x *1/c.2846A>T)  - 4x gene activity score 1 (*1/*2A)  Results:   \| Result for carriers of a gene variant on reduced dose compared to non-carriers on the normal dose: \| \| \| \| --- \| --- \| --- \| \| outcome \|  \| value for non-carriers \| \| % of patients with severe toxicity \| NS \| 37.9% \| \| The % of patients with severe toxicity was numerically lower for carriers of a gene variant on reduced dose (27.3%). \| \| % of patients with diarrhoea grade ≥ 3 \| NS \| 19.5% \| \| % of patients with diarrhoea resulting in hospitalisation \| NS \| 11.5% \| \| % of patients with hand-foot syndrome grade ≥ 2 \| NS \| 20.1% \| \| % of patients with haematological toxicity grade ≥ 3 \| NS \| 3.4% \| \| total prevalence of severe toxicities \| NS \| 43.1% \| \| hospitalisations \| NS \| 11.5% \| \| median days of hospitalisation \| NS \| 8 days \| \| cycle of first severe toxicity \| NS \| 2 \|  \| - Of the 6 patients with genotype *1/c.1236G>A:  - 2 tolerated a dose increase in cycle 3 (to 83% or 98% of the normal dose). The patient with the highest increased dose developed disease progression in cycle 3. The other patient only moderately tolerated the dose increase.  - 1 received an initial dose increase (to 85% of the normal dose in cycle 2 and 93% in cycle 3), but due to the general condition changing from good to poor, the dose increase was reversed (decrease to 85% of the normal dose in cycle 4 and to 75% in cycle 6).  - 2 were maintained on the starting dose, 1 due to a moderate general condition combined with the absence of toxicity and 1 due to grade 3 neutropenia in cycle 2 being accepted. The first patient developed disease progression (in cycle 2).  - 1 received a dose reduction to 66% in cycle 2 after grade 3 diarrhoea occurred in cycle 1. In this patient the colon was resected.  - The mean dose in the last cycle was 78% of the normal dose for these 6 patients. \| \| --- \| \| - The only patient with genotype *1/c.2846A>T tolerated a dose increase in cycle 3 to 85% of the normal dose. She developed disease progression in cycle 9. \| \| - Of the 4 patients with gene activity score 1 (*1/*2A):  - 1 tolerated three dose increases (to 57% of the normal dose in cycle 2, 63% in cycle 3 and 73% in cycle 4). The patient received a colon resection in cycle 6.  - 1 developed grade 3 diarrhoea after dose increase to 59% of the normal dose in cycle 2.  - 2 were maintained on the starting dose due to a mode-rate general condition (and either no or grade 1 toxicity). Both developed disease progression (in respectively cycle 3 and 6).  - The mean dose in the last cycle was 54% of the normal dose for these 4 patients. \|   NOTE: Because 42% of patients were not genotyped for c.1236G>A, approximately 4 patients in the group without gene variant, treated with the normal dose, were actually *1/c.1236G>A.  However, reducing the number of patients experiencing toxicity in the group without a gene variant with 4, still results in a numerically higher percentage of patients in the group without gene variant experiencing toxicity than in the group with gene variant (36% versus 24%).  NOTE: All patients were genotyped for *2A and c.2846A>T. 58% of patients was also genotyped for *13 and c.1236G>A. *13 was not found in these patients. | Authors’ conclusion:  ‘Tolerance-based capecitabine dose escalation did not lead to more toxicity in DPYD variant carriers compared with wild-type patients.’  Tolerated dose compared to AS 2:  AS 1.5: 79%  AS 1: 54% |
| ref. 2 – CAP, mono/comb  Lunenburg CATC et al.  Diagnostic and therapeutic strategies for fluoropyrimidine treatment of patients carrying multiple DPYD variants.  Genes (Basel)  2018;9:E585.  PubMed PMID:  30487465.  ref. 2, continuation | Level of evidence score: 2  PHENO: CTC-AE 5  PHENO on 50% of the normal dose: Clinical Relevance Score AA | 6 patients with multiple gene variants were identified either by additional retrospective genotyping after development of toxicity grade ≥ 3 from capecitabine-containing therapy in the study of Deenen 2016 (n = 4) or prior to treatment in routine clinical care (n = 2). One of these patients (with *2A and c.2846A>T) was already described in Lunenburg 2016, but was included because the data on DPD activity are new for this patient. A 7^th^ patient (c.1236G>A/c.2846A>T), already described in Henricks 2017, was not included in this summary. DPD enzyme activity in peripheral blood mononuclear cells was deter-mined either retrospectively after the occurrence of toxicity grade ≥ 3 (n = 4) or during treatment (n = 2).  Genotyping:  - 6x ‘phenotyping’ (3x *2A/c.1236G>A or *1/*2A+c.1236G>A, 3x *2A/c.2846A>T or *1/*2A+c.2846A>T)  Results:   \| - The 6 patients had 1%, 9%, 16%, 38%, 60% or 72% of the normal DPD activity (9%, 16% and 38% for *2A/c.1236G>A or *1/*2A+c.1236G>A; 1%, 60% and 72% for *2A/c.2846A>T or *1/*2A+c.2846A>T). Of the 2 patients with a DPD activity higher than 50% of normal, the patient with 72% of the normal DPD activity was verified to be *1/*2A+c.2846A>T (gene variants on the same allele), but the patient with 60% of the normal DPD activity was verified to be *2A/c.2846A>T (gene variants on separate alleles).  - the 4 patients with the lowest DPD activity had developed toxicity grade ≥ 3 (1x grade 3, 2x grade 4, 1x grade 5) on the normal dose. Three of them were admitted to hospital for 7-14 days. The authors indicate that low DPD enzyme activities might be due to neutropenia, because DPD activity is measured in mononuclear cells.  - the 2 patients with the highest DPD activity tolerated 50% of the normal starting dose (toxicity grade 0 for the patient with 72% of the normal DPD activity (starting dose based on the detection of the *2A allele prior to treatment in Deenen 2016) and grade 1-2 for the patient with 60% of the normal DPD activity (starting dose based on the genotype and the previous tolerance for adjuvant therapy with 5-fluorouracil 600 mg/m^2^ as described earlier in Lunenburg 2016)). A dose increase was not attempted for these patients. The palliative therapy for the patient with 60% of the normal DPD activity was discontinued due to the side effects. \| \| --- \|   NOTE: Genotyping was for *2A, *13, c.1236G>A and c.2846A>T. | Authors’ conclusion:  ‘In patients carrying  multiple DPYD variants, we recommend that a DPD phenotyping assay be carried out to determine a safe starting dose.’  Tolerated dose compared to AS 2:  PHENO: 50%  DPD activity compared to AS 2:  PHENO: 33% (66% if measured prior to toxicity) |
| ref. 3 – CAP/FU, mono/comb  Henricks LM et al. Effectiveness and safety of reduced-dose fluoropyrimidine therapy in patients carrying the DPYD*2A variant: a matched pair analysis.  Int J Cancer  2018 Nov 28 [Epub ahead of  print].  PubMed PMID: 30485432.  ref. 3, continuation  ref. 3, continuation  ref. 3, continuation  ref. 3, continuation | Level of evidence score: 3  AS 1 on genotype guided dose: Clinical Relevance Score AA  genotype guided versus not-genotype guided therapy for AS  1: Clinical Relevance Score AA^#^ | 40 patients with genotype *1/*2A and treated with an approximately 50% reduced fluoropyrimidine dose were compared to patients without *2A and to *1/*2A treated with full dose. To compare efficacy, 37 *1/*2A patients treated with reduced dose were matched to patients without *2A, *13, c.1236G>A, and c.2846A>T, treated with full dose. Only 13 matched pairs could be evaluated for disease control. To compare safety, *1/*2A patients treated with a reduced dose were compared with 1606 patients without *2A treated with full dose from Deenen 2016 and with 86 historical controls (*2A-carriers treated with full dose; including the historical controls in Deenen 2016).  16 of the 40 *1/*2A patients were from the study of Deenen 2016. The mean dose during the first cycle of these patients was 52% of the normal dose and during the entire treatment duration 53%. It was allowed to titrate the dose upwards during treatment after two cycles based on tolerance, as decided by the treating physician. In 11 patients, doses were titrated upwards during treatment, in 7 patients doses had to be further reduced after the initial dose reduction of 50%.  Overall survival was defined as the time between initiation of treatment and death by any cause. Progression-free survival was defined as the time between initiation of treatment and first signs of disease progression by either radiology or clinical signs, or death, whichever came first. Objective tumour response was defined according to RECIST 1.1 criteria. Disease control was defined as complete response, partial response or stable disease.  When anticipating a 15% decrease in overall survival (from 45% in the group without *2A to 30% in the *1/*2A-group at the end of the study, with a hazard ratio of no *2A versus *1/*2A of 0.66), 154 pairs (192 events in total) will be needed to reach 80% power, with a 5% significance level. With the  37 pairs of patients in this study, only a difference of at least 33% is detectable with 80% power.  Genotyping:  - 1606x gene activity score 2 (37 for efficacy analysis)  - 40x gene activity score 1 (*1/*2A) (37 for efficacy analysis)  Results:   \| Result for *1/*2A on approximately 50% of the normal dose compared to patients without *2A on the normal dose: \| \| \| \| --- \| --- \| --- \| \| outcome \|  \| value for patients without *2A \| \| median overall survival \| NS \| 24 months \| \| median progression-free survival \| NS \| 10 months \| \| % of patients with con-trolled disease \| NS \| 48% \| \| overall toxicity grade ≥ 3 \| NS \| 23% \| \| hand-foot syndrome  grade 3 \| NS \| 5% \| \| haematological toxicity  grade ≥ 3 \| NS \| 10% \| \| gastrointestinal toxicity  grade ≥ 3 \| NS \| 9% \| \| treatment interruptions \| NS \| 19% \| \| treatment discontinuation  due to toxicity \| NS \| 16% \| \| treatment-related hospitalisation \| NS \| 11% \| \| treatment-related death \| NS \| 0% \|  \| Result for *1/*2A on approximately 50% of the normal dose compared to *2A-carriers on the normal dose: \| \| \| \| --- \| --- \| --- \| \| outcome \|  \| value for *2A-carriers on the nor-mal dose \| \| overall toxicity grade ≥ 3 \| RR = 0.23 (95% CI: 0.12-0.45) (S) \| 77% \| \| haematological toxicity  grade ≥ 3 \| x 0.18 (S) \| 56% \| \| gastrointestinal toxicity  grade ≥ 3 \| x 0.26 (S) \| 38% \| \| treatment-related death \| trend for a decrease (p = 0.096) (NS) \| 8% \|   NOTE: Genotyping was for *2A. For the 37 patients without *2A included in the efficacy analysis, the presence of c.1236G>A, c.2846A>T and *13 was excluded. | Authors’ conclusion:  ‘Our study is the first to show that DPYD *2A genotype-guided dosing appears to have no negative effect on effectiveness of fluoropyrimidine-based chemotherapy, while resulting in significantly improved patient safety.’ |
| ref. 4 – CAP/FU, mono  Lunenburg CATC et al.  Standard fluoropyrimidine dosages in chemoradiation therapy result in an increased risk of severe toxicity in DPYD variant allele carriers.  Eur J Cancer 2018;104:210-8. PubMed PMID: 30361102.  ref. 4, continuation  ref. 4, continuation  ref. 4, continuation  ref. 4, continuation  ref. 4, continuation | Level of evidence score: 3  AS 1-1.5 on genotype guided dose: Clinical Relevance Score AA  AS 1-1.5 + PHENO: CTC-AE 4  genotype guided versus not-genotype guided therapy for AS  1-1.5: Clinical Relevance Score AA^#^ | 828 patients were treated with chemoradiation with a fluoropyrimidine. 22 patients with DPYD variant alleles treated with a reduced fluoropyrimidine dose and 34 patients with DPYD variant alleles treated with the normal dose were compared to 771 patients without variant alleles treated with the normal dose. In addition, a *1/*2A case started on reduced dose followed by a strong dose increase was described. Starting doses in 22 patients with DPYD variant alleles were reduced to 50% or 75% of the normal starting dose according to Dutch Pharmacogenetics Working Group 2015 and Clinical Pharmacogenetics Implementation Consortium 2017 guidelines (i.e. 50% of the normal dose for *1/*2A, 60% for *1/c.2846A>T and 50-75% for *1/c.1236G>A). 495 of the 828 patients were from the study of Deenen 2016. In addition, 240 patients from the Leiden University Medical Center and 93 from the Italian Cancer Institute were included in the analysis. Differences in toxicities were observed between the locations. In chemoradiotherapy the fluoropyrimidine dosing is different (for example capecitabine 825 mg/m^2^ twice daily continuously instead of 1250 mg/m^2^ twice daily for 2 weeks followed by 1 week rest for (colo)rectal cancer).  Of the gastrointestinal toxicity, diarrhoea and mucositis were scored for all of the included patients, but nausea and vomiting were only scored in the Leiden plus Italian groups and the Leiden group respectively.  Genotyping:   \| Reduced dose group: \| Normal dose group: \| \| --- \| --- \| \|  \| - 771x gene act. 2 (*1/*1) \| \| - 12x gene act. 1.5 (11x *1/c.1236G>A, 1x *1/c.2846A>T) \| - 20x gene act. 1.5 (20x *1/c.1236G>A, 9x *1/c.2846A>T) \| \| - 10x gene act. 1 (*1/*2A) \| - 3x gene act. 1 (2x *1/*2A, 1x *1/*13) \| \|  \| - 2x ‘PHENO’ (c.1236G>A/c.1236G>A) \|   Results:   \| Result for gene variant carriers on reduced dose compared to gene activity 2 on the normal dose: \| \| \| \| --- \| --- \| --- \| \| outcome \|  \| value for gene act. 2 on normal dose \| \| overall toxicity grade ≥ 3 \| NS \| 13.6% \| \| haematological toxicity  grade ≥ 3 \| trend for an increase (p = 0.083) (NS) \| 2.9% \| \| gastrointestinal toxicity  grade ≥ 3 \| NS \| 8.0% \| \| dose reductions \| NS \| 4.4% \| \| dose increases \| NS \| 0.5% \| \| treatment interruptions \| NS \| 4.9% \| \| prematurely stopped \| NS \| 9.9% \| \| treatment-related hospitalisation \| NS \| 7.8% \| \| days of hospitalisation \| NS \| 13 \|  \| Result for gene variant carriers on normal dose compared to gene activity 2 on normal dose: \| \| \| \| --- \| --- \| --- \| \| outcome \|  \| value for gene act. 2 on normal dose \| \| overall toxicity grade ≥ 3 \| NS \| 13.6% \| \| haematological toxicity  grade ≥ 3 \| OR_adj_ = 4.19 (95% CI: 1.32-13.25) (S) \| 2.9% \| \| gastrointestinal toxicity  grade ≥ 3 \| OR_adj_ = 2.58 (95% CI: 1.02-6.53) (S) \| 8.0% \| \| dose reductions \| NS \| 4.4% \| \| Doses were reduced from 100 to 60-77%. \| \| dose increases \| NS \| 0.5% \| \| treatment interruptions \| NS \| 4.9% \| \| prematurely stopped \| NS \| 9.9% \| \| treatment-related hospitalisation \| NS \| 7.8% \| \| days of hospitalisation \| NS \| 13 \|  \| Result for gene variant carriers on reduced dose compared to gene variant carriers on the normal dose: \| \| \| \| --- \| --- \| --- \| \| outcome \|  \| value for carriers on normal dose \| \| treatment-related hospitalisation \| NS \| 17.6% \| \| days of hospitalisation \| x 0.17 (S) \| 23 \|  \| - One *1/*2A was started on 50% of the normal dose. Despite diarrhea grade I-II after 2 weeks, the dose was increased to 83% of the normal dose. After 4 weeks, severe toxicity (diarrhoea, vomiting, nausea grade III and dermatitis grade II) occurred, and chemotherapy, and later radiotherapy, was stopped prematurely. The patient was hospitalised for 31 d, of which 3 d at the intensive care unit. After hospitalisation, the patient had to recover completely from toxicity for 39 d in a nursing home (rehabilitation). \| \| --- \|   NOTE: Genotyping was for *2A, *13, c.1236G>A and c.2846A>T. | Authors’ conclusion:  ‘DPYD variant allele carriers who received dose reductions  (n = 22) showed a comparable frequency of severe gastrointestinal toxicity compared with wild-type patients, but more (not statistically significant) severe haematological toxicity. Hospitalisations for all DPYD variant allele carriers were com-parable, independent of dose adjustments; however, the mean duration of hospitalisation was significantly shorter in the dose reduction group.’ |
| ref. 5 – CAP/FU, mono/comb  Henricks LM et al. DPYD genotype-guided dose individualisation of fluoropyrimidine  therapy in patients with cancer: a prospective safety analysis. Lancet Oncol  2018;19:1459-67.  PubMed PMID: 30348537  and personal communication (titrated dose and median DPD activity)  ref. 5, continuation  ref. 5, continuation  ref. 5, continuation  ref. 5, continuation  ref. 5, continuation  ref. 5, continuation | Level of evidence score: 3  AS 1.5: CTC-AE 5 (2)^#^  AS 1.5 on 75% of the normal starting dose: CTC-AE 3  AS 1 on 50% of the normal starting dose: Clinical Relevance Score AA | 1103 patients with either no or a single gene variant were treated with genotype-guided fluoropyrimidine-based chemo-therapy for a median of 71 days. The median number of treatment cycles was 3. Patient without a gene variant received the normal starting dose. *1/*2A and *1/*13 received 50% of the normal starting dose. *1/c.1236G>A and *1/c.2846A>T received 75% of the normal starting dose. Dose increase was allowed after the first two cycles provided that treatment was well tolerated, and the decision was left to the discretion of the treating physician. For one *1/*c.2846A>T no dose reduction was applied. The patient died after two cycles on full dose. Doses were increased more than 4 times as often in patients with a gene variant (13% of patients) than in patients without a gene variant (3% of patients). The dose increase was not well tolerated in 6 of the 11 patients with a gene variant (2 of the 4 *1/*2A, 3 of the 4 *1/c.1236G>A, 1 of the 2 *1/c.2846A>T and 0 of the 1 *1/*13), resulting in the *1/c.2846A>T discontinuing therapy because of toxicity and a dose reduction in the other patients. So, in 8% of the 85 patients with a gene variant, intolerance/toxicity was caused by either a dose increase or the starting dose not being reduced.  Toxicities scored as possibly, probably, or definitely related to fluoropyrimidine treatment were considered treatment-related. DPD enzyme activity in peripheral blood mononuclear cells was determined in pre-treatment samples (in 56 patients with a gene variant and 82 patients without).  Relative toxicity risks for patients with a gene variant and patients without a gene variant were compared to the relative risks in a historical control of patients receiving not-genotype- guided therapy (i.e. the normal starting dose for all patients) (the meta-analysis of Meulendijks 2015).  Sample size calculations revealed a required sample size of 11 variant carriers.  Genotyping:  - 1018x gene activity score 2 (*1/*1)  - 68x gene activity score 1.5 (51x *1/c.1236G>A, 17x *1/c.2846A>T)  - 17x gene activity score 1 (16x *1/*2A, 1x *1/*13)  Results:   \| Result for gene variant carriers on reduced dose compared to patients without gene variant on the normal dose: \| \| \| \| \| --- \| --- \| --- \| --- \| \|  \| \|  \| value for patients without gene variant \| \| overall grade ≥ 3 toxicity \| all carriers \| x 1.7 (S) \| 23% \| \|  \|  \| \|  \|  \| \| *1/c.1236G>A \| RR = 1.69 (95% CI: 1.18-2.42) (S) \| \| *1/c.2846A>T \| RR = 2.00 (95% CI: 1.19-3.34) (S) \| \| *1/*2A \| NS \| \| *1/*13 \| - \| \| grade ≥ 3 gastrointestinal toxicity \| \| x 2.5 (S) \| 8% \| \| grade ≥ 3 haematological toxicity \| \| x 2.5 (S) \| 6% \| \| grade 3 hand-foot syndrome \| \| NS \| 4% \| \| grade ≥ 3 cardiac toxicity \| \| NS \| 1% \| \| grade ≥ 3 other treatment-related toxicity \| \| NS \| 8% \| \| overall grade ≥ 4 toxicity \| \| NS \| 3% \| \| grade ≥ 4 gastrointestinal toxicity \| \| NS \| 1% \| \| grade ≥ 4 haematological toxicity \| \| NS \| 1% \| \| grade ≥ 4 cardiac toxicity \| \| NS \| <1% \| \| grade ≥ 4 other treatment-related toxicity \| \| NS \| 1% \| \| fluoropyrimidine-related hospital admission \| \| NS \| 14% \| \| stop of fluoropyrimidines because of adverse  events \| \| NS \| 17% \| \| fluoropyrimidine-related death \| \| NS \| <1% \|  \| Results per variant genotype: \| \| \| \| \| \| --- \| --- \| --- \| --- \| --- \| \|  \| *1/  c.1236G>A \| *1/  c.2846A>T \| *1/*2A \| *1/*13 \| \| % of normal dose in first cycle \| 74 \| 73 \| 51 \| 50 \| \| % of normal dose whole treatment \| 74 \| 72 \| 53 \| 54 \| \| titrated dose \| 74 \| 64 \| 57 \| 63 \| \| DPD-enzyme activity (% of normal) \| 80 \| 66 \| 55 \| 40 \| \| median DPD-enzyme activity (% of normal) \| 74 \| 67 \| 56 \| - \|  \| Relative risk (95% CI) for overall grade ≥ 3 toxicity for carriers on reduced dose compared to carriers on the normal dose: \| \| \| \| --- \| --- \| --- \| \|  \| reduced dose \| normal dose \| \| c.1236G>A \| 1.69 (1.18-2.42) (S) \| 1.72 (1.22-2.42) (S) \| \| c.2846A>T \| 2.00 (1.19-3.34) (S) \| 3.11 (2.25-4.28) (S) \| \| *2A \| 1.31 (0.63-2.73) (NS) \| 2.87 (2.14-3.86) (S) \| \| *13 \| - \| 4.30 (2.10-8.80) (S) \|   NOTE: The authors did not investigate whether the overall grade ≥ 3 toxicity in carriers on reduced dose after correction for the toxicity caused by dose increase and exclusion of the patient that never received a dose reduction was still significantly higher than in patients without gene variant on full dose. This correction reduces the overall grade ≥ 3 toxicity in all carriers from 39% to 31%, in *1/c.1236G>A from 39% to 33% and in *1/c.2846A>T from 47% to 38%. (For *1/*2A, this correction reduces the overall grade ≥ 3 toxicity from 31% to 19%, while the value for *1/*13 remains 0%.)  NOTE: The authors indicated that although the mean DPD enzyme activity for *1/c.1236G>A was reduced by around 20%, the large variation in this activity suggests that a proportion of patients needs a larger dose reduction, while other patients might tolerate a full dose. However, the absence of a correlation between DPD enzyme activity and the occurrence of severe fluoropyrimidine-related toxicity for each of the genotypes, also for *1/236A (and for *1/c.2846A>T), argues against an important role of DPD activity variation in the occurrence of toxicity.  NOTE: In the discussion, the authors mentioned twice that the applied genotype-guided dose reduction is unlikely to result in under dosing/undertreatment. However, they did not address this issue in their recommendation of a more cautious initial dose reduction of 50% for *1/236A and *1/c.2846A>T, followed by close monitoring and individual dose titration. Because the titrated dose in these patients is respectively 74% and 64% of the normal dose and corresponding percentages for the remaining mean and median DPD activity were found, a starting dose of 50% would be under dosing for the majority of these patients.  NOTE: Genotyping was for *2A, *13, c.1236G>A and c.2846A>T. | Authors’ conclusion:  ‘Prospective DPYD genotyping was feasible in routine clinical practice, and DPYD genotype-based dose reductions improved patient safety of fluoropyrimidine treatment. For DPYD*2A and c.1679T>G carriers, a 50% initial dose reduction was adequate. For c.1236G>A and c.2846A>T carriers, a larger dose reduction of 50% (instead of 25%) requires investigation.’  Titrated dose compared to AS 2:  AS 1.5: 72%  AS 1: 57%  DPD activity compared to AS 2:  AS 1.5: 77%  AS 1: 53% |
| ref. 6 – CAP/FU, comb  Madi A et al.  Pharmacogenetic analyses of 2183 patients with advanced colorectal cancer;  potential role for common dihydro-pyrimidine dehydrogenase variants in toxicity to chemotherapy. Eur J Cancer 2018;102:31-9. PubMed PMID: 30114658.  ref. 6, continuation  ref. 6, continuation  ref. 6, continuation  ref. 6, continuation  ref. 6, continuation | Level of evidence score: 3  AS 1.5: CTC-AE 3  AS 1: CTC-AE 4 | 2116 patients with advanced colorectal cancer were treated with fluoropyrimidine-oxaliplatin chemotherapy with or without cetuximab for 24 weeks. 62% of patients received capecitabine plus oxaliplatin and 38% received infusional 5-fluorouracil plus oxaliplatin. 37% of patients also received cetuximab. After 12 weeks, therapies also differed in being either continuous or intermittent.  Any 12-week toxicity was defined as a dose reduction or delay in chemotherapy in the first 12 weeks of treatment due to any toxicity except peripheral neuropathy. Peripheral neuropathy is an oxaliplatin-associated toxicity.  Associations were tested with a codominant model (i.e. homozygous variant versus heterozygous versus homozygous wild type). Odds ratios were calculated using the best model that fitted the data (dominant (i.e. homozygous+heterozygous variant versus homozygous wild type), recessive (i.e. homozygous variant versus heterozygous+homozygous wild type), or additive (i.e. homozygous variant versus heterozygous versus homozygous wild type)) and were adjusted for cetuximab use and type of fluoropyrimidine.  The power was > 85% to detect odds ratios of 1.3 for variants with frequencies > 20% and to detect odds ratios of 1.6 for variants with frequencies > 5%. This corresponds to respectively a 7% difference in response or toxicity (45% responded and 35% had toxicity) and an 11% difference in response.  Genotyping:   \| c.2846A>T (Asp949Val): \| *2A (2105 patients genotyped): \| \| --- \| --- \| \| - 2086x *1/*1 \| - 2082x *1/*1 \| \| - 30x *1/c.2846A>T \| - 23x *1/*2A \|   Results:   \| Results for gene variant carriers compared to patients without the gene variant: \| \| \| \| \| --- \| --- \| --- \| --- \| \|  \| c.2846A>T \| *2A \| value for patients without gene variant \| \| dose reduction or therapy delay in the first 12 weeks due to any toxicity except peripheral neuropathy \| OR = 2.2 (95% CI: 1.1-4.5)  (S, but NS after correction for the 8 tested gene variants) \| NS \| 36% \| \| neutropenia grade ≥ 2 \| OR = 3.2 (95% CI: 1.2-8.2)  (S, but NS after correction for the 8 tested gene variants) \| NS \| 13% \| \| lethargy grade ≥ 2 \| NS \| OR = 5.3 (95% CI: 1.9-14.9)  (S, also after correction for the 8 tested gene variants) \| 34% \| \| nausea and vomiting grade ≥ 2 \| OR = 3.4 (95% CI: 1.5-7.3)  (S, also after correction for the 8 tested gene variants) \| NS \| 20% \| \| diarrhoea grade ≥ 2 \| OR = 4.6 (95% CI: 2.1-10.1)  (S, also after correction for the 8 tested gene variants) \| OR = 4.4 (95% CI: 1.7-11.0)  (S, also after correction for the 8 tested gene variants) \| 25% \| \| stomatitis grade ≥ 2 \| NS \| OR = 4.6 (95% CI: 1.7-12.6)  (S, also after correction for the 8 tested gene variants) \| 10% \| \| hand-foot syndrome grade ≥ 2 \| NS \| OR = 3.8 (95% CI: 1.2-11.8)  (S, but NS after correction for the 8 tested gene variants) \| 9% \| \| infection with neutropenia grade ≥ 3 \| OR = 5.5 (95% CI: 1.3-24.2)  (S, but NS after correction for the 8 tested gene variants) \| OR = 19 (95% CI: 5.0-73.8)  (S, also after correction for the 8 tested gene variants) \| 3% \|   NOTE: Only data on the gene variants proven to be associated with toxicity and reduced DPD enzyme activity were included in the table above.  For the common gene variants *9A (Cys29Arg) and *6 (Val732Ile), this study found an association with a decrease and an increase in any toxicity in the first 12 weeks respectively. However, significance disappeared after Bonferroni correction for multiple testing. For *9A, no associations with specific toxicities were found. For *6, this study found an association with neutropenia grade ≥ 2, but significance disappeared after correction for the testing of 8 variants of the DPD encoding gene.  The study found an association of *4 (Ser534Asn) with any toxicity in the first 12 weeks, but not with specific toxicities and significance was lost after correction for the testing of 8 variants of the DPD encoding gene.  The study found an association of c.775A>G (Lys259Glu) with stomatitis, but significance was both lost after calculation of an odds ratio and after correction for the testing of 8 variants of the DPD encoding gene. The study found no association of c.496A>G (Met166Val) and c.1627A>G (Ile543Val) with either any or specific toxicity.  So, the study confirms the lack of sufficient proof for an association with toxicity for these gene variants.  NOTE: Genotyping was for the indicated gene variants. | Authors’ conclusion:  ‘Our data suggest that both common and rare DPYD variants may be associated with toxicity to fluoropyrimidine-based chemotherapy. …. No common variant associations remained significant after Bonferroni correction.’ |
| ref. 7 – CAP, comb  Henricks LM et al. Capecitabine-based treatment of a patient with a novel *DPYD* genotype and complete dihydropyrimidine dehydrogenase deficiency.  Int J Cancer  2018; 142:424-30 PubMed PMID: 28929491. | Level of evidence score: 2  AS 0: Clinical Relevance Score A | A 59-year-old women with 0.5% of the normal DPD activity tolerated adjuvant chemotherapy with 0.8% of the normal capecitabine dose (77 mg/m^2^ on days 1 and 6 of the first cycle and on days 1, 6 and 11 of the following cycles) in combination with oxaliplatin for eight cycles. Capecitabine-related toxicity like diarrhoea, hand-foot syndrome or leukopenia did not occur. However, sensory neuropathy developed during the first cycle, and became more severe (grade 3) during the second cycle. Because this was most likely caused by oxaliplatin, the oxaliplatin dose was decreased to 75% from the third cycle onwards and discontinued after the sixth cycle.  The dose-corrected AUC of 5-FU in this patient was 11.271% of that of control patients.  Her genotype was *2A/(duplication of exon 17 and 18).  NOTE: The patient was initially genotyped for *2A, *13, c.2846A>T and c.1236G>A. Additional gene variants were not found by sequencing of all 23 coding exons and flanking intronic regions, after which copy numbers of sequences were analysed. | Authors’ conclusion:  ‘This case report demonstrates that a more comprehensive genotyping and phenotyping approach, combined with pharmacokinetically-guided dose  administration, enables save fluoropyrimidine treatment with adequate drug exposure in completely DPD deficient patients.’  Dose-corrected AUC versus gene activity 2:  gene act. 0: 11271% |
| ref. 8 – CAP/FU, mono/comb  Henricks LM et al. Treatment algorithm for homozygous or com-pound heterozygous DPYD variant allele carriers with low-dose capecitabine. JCO Precis Oncol - published online 2017 Oct 6.  ref. 8, continuation  ref. 8, continuation  ref. 8, continuation  ref. 8, continuation | Level of evidence score: 2  PHENO: Clinical Relevance Score A  AS 0: Clinical Relevance Score A  PHENO: Clinical Relevance Score A | 5 patients, being either homozygous for a gene variant or having two different gene variants, received capecitabine or 5-fluorouracil treatment with doses based on the pre-treatment DPD activity in peripheral blood mononuclear cells. Pre-treatment DPD activity was also determined in a patient with genotype c.2846A>T/c.2846A>T, who did not receive treatment, because she was disease free after surgery. For 3 patients, the AUC of fluorouracil after the first dose of capecitabine was determined, normalised to a dose of 850 mg/m^2^ and compared to 22 patients from another study receiving combined chemotherapy with capecitabine 850 mg/m^2^.  Genotyping:  - 2x c.1236G>A/c.1236G>A - 2x c.2846A>T/c.2846A>T  - 1x *2A/*2A  - 1 carrier of both c.1236G>A and c.2846A>T (verified as c.1236G>A/c.2846A>T (variants on separate alleles) by Lunenburg, Genes 2018)  Results:   \| - Of the four patients homozygous for a partially functional allele, the two patients with genotype c.1236G>A/c.1236G>A had respectively 79% and 42% of the normal DPD activity. The first was treated with 75% of the normal capecitabine dose in cycle 1 and with 100% in cycle 2. The second was treated with 50% of the normal 5-fluorouracil dose. The patients did not have severe toxicity on the reduced doses.  The two patients with genotype c.2846A>T/c.2846A>T had respectively 29% and 10% of the normal DPD activity. The first was treated with 17% of the normal capecitabine dose (278 mg/m^2^ once daily in combination with radiotherapy as neoadjuvant treatment) and the second was the patient who did not need treatment. The first patient tole-rated treatment well without occurrence of severe toxicity and surgery was performed after treatment. The dose-corrected AUC of 5-fluorouracil in this patient was 866% of that of control patients.  The mean DPD activity in these patients was 40%. There was a large variance in DPD activity between these patients (10-79%). \| \| --- \| \| - The patient with genotype *2A/*2A had undetectable DPD activity and tolerated monotherapy with 0.65% of the normal capecitabine dose (65 mg/m^2^ every 5 days) for 1 month after which grade 2 diarrhoea developed. After a rest period of 3 weeks, treatment was restarted with the same dose, but every third gift was skipped (0.43% of the normal dose). The patient tolerated this dose also after addition of oxaliplatin and bevacizumab as originally planned and had stable metastatic colorectal carcinoma as best treatment response. The dose-corrected AUC of 5-fluorouracil in this patient was 13.812% of that of control patients. \| \| - The carrier of both c.1236G>A and c.2846A>T had 45% of the normal DPD activity, corresponding to a patient with variants on different alleles. He was treated with 51% of the normal capecitabine dose in cycle 1 (daily dose of 900 mg/m^2^ in combination with oxaliplatin), which was tolerated without toxicity. Increase to 71% of the planned dose (daily dose of 1250 mg/m^2^) in cycle 2 resulted in grade 3 thrombocytopenia. The dose was reduced to 57% of the normal dose (1000 mg/m^2^ daily), which was continued during cycle 3. However, because grade 2 thrombocytopenia developed after 8 days, the dose was reduced to 29% of the normal dose (500 mg/m^2^ daily) for the rest of the cycle, resulting in platelets to increase to normal values. Progression of metastatic colorectal cancer was established after 3 cycles and capecitabine treatment was discontinued. The dose corrected AUC of 5-fluorouracil in this patient was 227% of that of control patients. \|   NOTE: Patients were genotyped for *2A, *13, c.2846A>T and c.1236G>A. | Authors’ conclusion:  ‘We showed that fluoropyrimidine treatment in homozygous or com-pound heterozygous  DPYD variant allele carriers is feasible and that therapy does not have to be withheld. Additional  DPD phenotyping tests, such as measurement of DPD activity in PBMCs, are recommended  to compose an individualized treatment. After an initial dose reduction, tolerability in patients should be monitored closely, and the dose should be individually titrated according to tolerance.’  Dose-corrected AUC versus AS 2:  PHENO: 546%  AS 0: 13812%  Tolerated dose compared to AS 2:  PHENO: 55%  AS 0: 0.43%  DPD activity compared to AS 2:  PHENO: 41%  AS 0: 0% |
| ref. 9 – FU, mono/comb  Meulendijks D et al.  Pretreatment serum uracil concentration as a predictor of severe and fatal fluoropyrimidine-associated toxicity.  Br J Cancer 2017;116:1415-24.  PubMed PMID: 28427087.  ref. 9, continuation  ref. 9, continuation | Level of evidence score: 4  AS 1: CTC-AE 4  AS 1-1.5: CTC-AE 4  AS 1.5: Clinical Relevance Score AA | 1606 *2A-negative patients from Deenen 2016 were genotyped for other gene variants.  Toxicity was defined as toxicity grade ≥ 3, global toxicity as any toxicity, hospitalisation as toxicity related hospitalisation. Only outcomes during the first cycle of chemotherapy were included.  ORs were adjusted for age, sex and treatment regimen.  Genotyping:  - 19 carriers of c.2846A>T  - 3 carriers of *13  - 58 carriers of c.1236G>A    Results:   \| Result for carriers compared to non-carriers of the gene variant: \| \| \| \| --- \| --- \| --- \| \| gene variant \| outcome \| OR_adj_ (95% CI) \| \| c.2846A>T \| global toxicity \| NS, trend for an increase (p = 0.095) \| \| gastrointestinal toxicity \| NS \| \| haematological toxicity \| NS, trend for an increase (p = 0.066) \| \| hospitalisation \| NS \| \| *13 \| global toxicity \| NS \| \| gastrointestinal toxicity \| NS, trend for an increase (p = 0.090) \| \| haematological toxicity \| 24.9 (1.74-354) (S) \| \| hospitalisation \| NS, trend for an increase (p = 0.094) \| \| c.2846A>T and *13 \| global toxicity \| 3.0 (1.05-8.77) (S) \| \| c.1236G>A \| global toxicity \| NS \| \| gastrointestinal toxicity \| NS \| \| haematological toxicity \| NS \| \| hospitalisation \| NS, trend for an increase (p = 0.069) \| \| For the 3 gene variants combined, sensitivity was 6%, specificity 95%, positive predictive value 13% and negative predictive value 88% for prediction of global toxicity grade ≥ 3 in the first cycle. \| \| \|   NOTE: No association was found for the gene variants *4 (84 carriers), except for a trend for gastrointestinal toxicity. However, most studies including a meta-analysis (Meulendijks 2015) do not show an association of this gene variant with toxicity. In addition, results regarding the effect on DPD activity are inconsistent. | Authors’ conclusion:  ‘None of the individual *DPYD* variants were found to be associated with global severe toxicity. For c.2846A>T and c.1679T>G combined, there was evidence for an association with global severe toxicity. In addition, *DPYD* c.1679T>G alone was associated with haematological toxicity.’ |
| ref. 10 – FU/CAP, mono/comb  Kodali S et al. Capecitabine-induced severe toxicity secondary to DPD deficiency and successful treatment with low dose 5-fluorouracil.  J Gastrointest Cancer 2017;48:66-69. PubMed PMID: 26744322. | Level of evidence score: 2  AS 1: CTC-AE 4 | A 51-year old male developed severe colitis with mucous stools (grade 4 toxicity) and neutropenic fever (neutrophils 0.18x10^9^/L) on day 21 of neoadjuvant treatment with standard dose capecitabine (825 mg/m^2^ twice daily) and radiotherapy. His genotype was *1/*2A.  The patient tolerated adjuvant therapy with 5-FU 300 mg/m^2^ per day as a continuous intravenous infusion (25% of the standard dose) and without bolus injections of 5-fluorouracil very well. Higher doses were not attempted, because they were judged not to influence recurrence or survival. | Authors’ conclusion:  ‘The utility of pharmacokinetic-based dosing remains questionable as patients experienced toxicity even with 50% dose reduction of 5-FU, as recommended by current consortium guidelines. We therefore suggest that dosing of 5-FU should be customized in patients with DPD deficiency based on clinical judgment taking into account the severity of toxicity from initial exposure.’ |
| ref. 11 – CAP, mono/comb  Meulendijks D et al.  Patients homozygous for *DPYD* c.1129-5923C>G/ haplotype B3 have partial DPD deficiency and require a dose reduction when treated with fluoropyrimidines. Cancer Chemother Pharmacol 2016;78:875-80. PubMed PMID: 27544765.  ref. 11, continuation  ref. 11, continuation | Level of evidence score: 2  PHENO: CTC-AE 2 | Three patients treated with capecitabine containing chemotherapy were retrospectively determined to have genotype c.1236G>A/c.1236G>A. Gene variants *2A, *13 and c.2846A>T were not present in these patients. More than 4 weeks after the last treatment with fluoropyrimidines, DPD enzyme activity in peripheral blood mononuclear cells was determined and cDNA was analysed.  Results:  - A 47-year old female developed leukocytopenia grade 2 (2.3x10^9^/L), neutropenia grade 2 (1.3x10^9^/L), hand-foot syndrome grade 1, diarrhoea grade 1 and fatigue grade 1 on day 9 of neoadjuvant treatment with standard dose capecitabine (825 mg/m^2^ twice daily) and radiotherapy. Because the symptoms intensified, the capecitabine dose was reduced by 40% on day 15. After dose reduction, treatment was well tolerated. Five days after a dose increase by 10%, she again developed leukopenia grade 2 (2.5x10^9^/L) and neutropenia grade 1 (1.5x10^9^/L). Despite this, treatment could be finished at reduced dose. The patient received surgery and was disease-free four years after treatment.  The DPD activity of the patient was 41% of the normal DPD activity.  - A 67-year old male developed fatigue grade 2 on day 7 of treatment with capecitabine 850 mg/m^2^ on day 1-14 of the three-week cycle, docetaxel, oxaliplatin and bevacizumab. On day 11, the patient was hospitalised with neutropenia grade 2 (1.3x10^9^/L) and fever grade 1 (38.7^o^C, without apparent focus). After release from hospital, he refused further treatment. Because of disease progression, capecitabine 800 mg/m2 twice daily (64% of the standard dose) was started four months later as monotherapy. The patient again developed fatigue grade 2 and refused further treatment after cycle 1. The DPD activity of the patient was 55% of the normal DPD activity.  - A 69-year old male tolerated 4 weeks of neoadjuvant treatment with standard dose capecitabine (825 mg/m^2^ twice daily) and radiotherapy well. Treatment was completed without dose reductions or delays, and without adverse events and haematological changes. The patient had a relapse one year after surgery and died as a result of progressive disease before determination of DPD activity could be performed.  cDNA analysis of the first two patients showed that they produced roughly equal amounts of wild type mRNA and aberrantly spliced mRNA with a premature stop codon.  The authors indicate that the starting dose of capecitabine was relatively low in these patients (compared to the monotherapy dose of 1250 mg/m^2^ twice daily). So, higher doses might have resulted in more pronounced toxicity. Amstutz 2009 describes a patient with genotype c.1236G>A/c.1236G>A, who developed fatal toxicity during the first cycle with full dose 5-FU plus cisplatin.  NOTE: Patients were genotyped for c.1129-5923C>G and checked for the presence of c.1236G>A and c.959-51T>G, which are in complete linkage disequilibrium with c.1129-5923C>G in haplotype B3. | Authors’ conclusion:  ‘The presented functional and clinical data indicate that the c.1129-5923 C>G variant is both functionally and clinically relevant, and support an upfront dose reduction of the fluoropyrimidine starting dose in patients carrying c.1129-5923C>G homozygously.’  Tolerated dose versus gene activity 2:  gene activity 1: 60%  DPD activity versus gene activity 2:  gene activity 1: 48% |
| ref. 12 – FU/CAP, mono/comb  Lunenburg CA et al.  Evaluation of clinical implementation of prospective *DPYD* genotyping in 5-fluorouracil- or capecitabine-treated patients. Pharmacogenomics  2016;17:721-9. PubMed PMID: 27181275.  ref. 12, continuation  ref. 12, continuation  ref. 12, continuation | Level of evidence score: 3  AS  1.5:CTC-AE 4(2)^#^  AS  1:CTC-AE 4(2)^#^  PHENO on 50% of normal dose: CTC-AE 2 (2)^#^ | The results of routine prospective genotyping and genotype-guided dosing were retrospectively evaluated in patients receiving capecitabine or 5-fluorouracil, either as combined chemotherapy (different combinations) or as monotherapy (with or without radiotherapy). Genotyping was originally only for *2A (275 patients), but from approximately 30% of the total study time genotyping for *13 and c.2846A>T was added (214 patients) and from 65% of the total study time genotyping for c.1236G>A was added (n = 109). Recommended dosing reductions were 50% of the normal dose per *2A- and *13-variant and 25% per c.1236G>A-variant. Recommended dosing reduction per c.2846A>T-variant was 50% (change to a recommendation of 25% reduction was only after the study), but was not applied. 14 patients with gene variants were identified.  Due to the low number of patients with DPD variants the study was not powered to formally test the effect of genotype-guided dosing on fluoropyrimidine-induced toxicity and only explorative analyses could be performed.  Genotyping:  - 8x *1/c.1236G>A  - 5x *1/*2A  - 1 carrier of both *2A and c.2846A>T (either *2A/c.2846A>T (on separate alleles) or *1/(*2A+c.2846A>T) (variants on the same allele))  Results:   \| - 8 patients (5x *1/c.1236G>A and 3x *1/*2A) received the recommended initial dose reduction and did not develop toxicity grade 3-4 in cycle 1.  The dose of 4 patients was subsequently increased. Two patients (1x *1/c.1236G>A with a dose increase to 100% of the normal dose and 1x *1/*2A with a dose increase to 60% of the normal dose) did not develop toxicity grade 3-4. A patient with genotype *1/*2A developed diarrhoea grade 3 and enteritis after dose increase to 80% of the normal dose. Another patient with this genotype developed hand-foot-syndrome grade 2-3 after multiple cycles with the normal dose. \| \| --- \| \| - 3 patients (1x *1/c.1236G>A and 2x *1/*2A) did not receive an initial dose reduction and developed toxicity grade 3-4 in cycle 1. For two of these patients, therapy was started before the genotype was known. For the third patient, the oncologist did not reduce the dose, because the dose in the chemotherapy regimen was already relatively low (capecitabine plus radiotherapy). For 1 patient with genotype *1/*2A, the dose was subsequently reduced to 50% of the normal dose and the patient did not develop toxicity grade 3-4 anymore. The other 2 patients quitted fluoropyrimidine therapy. \| \| - For the carrier of both *2A and c.2846A>T, there was no dose recommendation, because it was not known whether the variants were on different alleles or on the same allele. Because therapy had to be started before the DPD-activity would have been determined, the physician decided to use a 50% dose reduction, taking into account the results of genotyping and that this patient had tolerated 5-FU containing regimens before. Fluoropyrimidine therapy was stopped in this patient after the first cycle due to toxicity (≤ grade 3). \| \| - 2 patients (both with genotype *1/c.1236G>A) did not start fluoropyrimidine therapy. \| | Authors’ conclusion:  ‘Prospective *DPYD* screening can be implemented successfully in a real-world clinical setting, is well accepted by physicians and results in low toxicity.’ |
| ref. 13 – FU, comb  Lee AM et al. Association between *DPYD* c.1129-5923 C>G/hapB3 and severe toxicity to 5-fluorouracil-based chemotherapy in stage III colon cancer patients: NCCTG N0147 (Alliance). Pharmacogenet Genomics 2016;26:133-7. PubMed PMID: 26658227.  ref. 13, continuation  ref. 13, continuation | Level of evidence score: 3  AS 1.5 + PHENO: CTC-AE 4 | A subset of patients from Lee 2014 was reanalysed: 1953 patients, negative for *2A, *13 and c.2846A>T, and treated with 12 cycles of adjuvant FOLFOX therapy (5-FU, folinic acid and oxaliplatin) with or without cetuximab. 62.9% of patients had any grade ≥ 3 adverse event, with 32.7% having any grade ≥ 3 adverse event common to 5-FU treatment.  Adverse events classified as common to 5-FU treatment were fatigue, anorexia, dehydration, diarrhoea, stomatitis/mucositis, nausea/vomiting, leukopenia, neutropenia, febrile neutropenia, thrombocytopenia, and pain. Most frequent 5-FU adverse events included diarrhoea (12.5%), neutropenia (10.3%), pain (5.4%), fatigue (5.2%), nausea/vomiting (4.7%), and mucositis (4.1%).  Results were adjusted for clinicopathological factors like age, sex, treatment, total number of treatment cycles and dose modifications. The latter two outcomes (higher percentage of patients with premature continuation and with dose modification) might be results of 5-FU adverse events instead of causes.  Cetuximab increased the risk of 5-FU adverse events. Results were adjusted for this, but this indicates that adverse events common to 5-FU are not the same as 5-fluorouracil-induced adverse events.  Genotyping:  - 1875x *1/*1  - 77x *1/c.1236G>A  - 1x c.1236G>A/c.1236G>A    Results:   \| Risk of grade ≥ 3 adverse event for c.1236G>A/c.1236G>A versus *1/c.1236G>A versus *1/*1: \| \| \| --- \| --- \| \| any adverse event \| NS, trend for an increase (p = 0.082) \| \| OR_adj_ for (*1/c.1236G>A + c.1236G>A/c.1236G>A) compared to *1/*1 also showed a trend for an increase (NS, p = 0.127). \| \| diarrhoea \| NS \| \| neutropenia \| S for an increase \| \| pain \| NS \| \| fatigue \| NS \| \| nausea/vomiting \| NS \| \| stomatis/mucositis \| NS \| \| dehydration \| NS \| \| leukopenia \| NS \|   NOTE: Results were reported for c.1129-5923C>G, which was in complete linkage disequilibrium with the also genotyped c.1236G>A. | Authors’ conclusion:  ‘No significant associations were identified between c.1129 -5923 C>G/hapB3 and overall grade≥3 adverse event rate. Our results suggest that c.1129-5923 C>G/hapB3 have limited predictive value for severe toxicity to 5-FU-based combination chemotherapy.’ |
| ref. 14 – FU/CAP, mono/comb  Deenen MJ et al. Upfront genotyping of *DPYD**2A to individualize fluoropyrimidine therapy: a safety and cost analysis. J Clin Oncol 2016;34:227-34. PubMed PMID: 26573078.  ref. 14, continuation  ref. 14, continuation  ref. 14, continuation  ref. 14, continuation  ref. 14, continuation | Level of evidence score: 3  AS 1 on 48% of the normal dose: Clinical Relevance Score AA  AS  1: Clinical Relevance Score A | 1631 patients received genotype-guided therapy with capecitabine (90% of patients) or 5-FU (10% of patients), either as combined chemotherapy (different combinations) or as monotherapy (with or without radiotherapy). Genotyping was for *2A. For *1/*2A, dose reduction in the first two cycles was ≥ 50% and was followed by dose titration based on tolerance. Initial dose was not reduced for *1/*1. Patients with the *1/*2A genotype were compared with 48 patients with this genotype, treated with the full initial dose in published cohorts studies without genotype-guided dosing. Of these 48 patients, 79% was treated with 5-fluorouracil, 19% with capecitabine and 2% with tegafur combined with uracil. In addition, patients with the *1/*2A genotype were compared to patients with the *1/*1 genotype.  For 16 *1/*2A-patients, 5-fluorouracil AUC in blood plasma after the first capecitabine dose was compared with that of 25 unselected patients from two studies (n = 11 and n = 14 per study).  For 15 *1/*2A-patients, DPD enzyme activity in peripheral mononuclear blood cells was determined and compared with the mean Caucasian DPD enzyme activity (mainly *1/*1-patients).  The study had 100% power to detect a reduction of the incidence of grade ≥ 3 toxicity in *2A-carriers from 85% to 20%.  The risk of grade ≥ 3 toxicity was higher in combination therapy than in monotherapy and chemo-radiotherapy regimens.  Genotyping:  - 1613x *1/*1  - 18x *1/*2A  Results:   \| Treatment characteristics of *1/*2A-patients: \| \| --- \| \| - The initial dose varied from 29% to 60% of the full dose (median 46%). The final dose varied from 17% to 91% of the full dose. The median dose per treatment cycle was 48% (range 17% to 91%). All patients were treated with capecitabine. \| \| - 5 patients developed toxicity grade ≥ 3 (first cycle 29% to 60% of the normal dose, final cycle 17% to 60% and maximum 29% to 67%)  - 2 patients developed toxicity grade 0 (first of the two cycles with 29% and second cycle with 59% of the nor-mal dose and all five cycles 48% of the normal dose, respectively)  - 11 patients developed toxicity grade 1 to 2 (first cycle 30% to 50% of the normal dose, final cycle 24% to 91% and maximum 46% to 91%)  - Toxicity was short in duration and well controlled using standard supportive care. \| \| - For 6 patients, the dose was increased during treatment (dose in first cycle 29% to 47% of the normal dose; maxi-mum dose 46% to 91%).  In two of these patients (dose increase from 47% to 53% and from 44% to 67%, respectively), the dose was later reduced to the initial dose again because of toxicity. \| \| - For 3 patients, the initial dose was still too high and had to be reduced further (initial dose 29% to 44% of the normal dose, final dose 17% to 24%). \| \| - Of 4 evaluable patients, 2 achieved a partial response and 2 had stable disease. In 4 of 5 patients with rectal cancer treated with chemo-radiotherapy, down staging of the tumour from pT3-4 to ypT0-2 was reached. \|  \| Percentage of *1/*2A patients with toxicity for reduced dosing compared to full dosing: \| \| \| \| --- \| --- \| --- \| \|  \|  \| value for full do-sing \| \| any grade ≥ 3 toxicity \| x 0.38 (S) \| 73% \| \| In addition, the observed toxicity was short in duration with reduced dosing and usually long-lasting with full dosing. \| \| \| grade ≥ 3 haematological toxicity \| x 0.26 (S) \| 66% \| \| grade ≥ 3 gastrointestinal toxicity \| x 0.20 (S) \| 56% \| \| fluoropyrimidine-induced death \| NS \| 10% \|  \| Percentage of patients with toxicity for *1/*2A on reduced dosing compared to *1/*1 on full dosing: \| \| \| \| \| --- \| --- \| --- \| --- \| \|  \|  \|  \| value for *1/*1 \| \| any toxicity \| grade ≥ 3 \| NS \| 23% \| \| grade 1-2 \| NS \| 54% \| \| haematological toxicity \| grade ≥ 3 \| NS \| 10% \| \| grade 1-2 \| NS \| 35% \| \| diarrhoea \| grade ≥ 3 \| NS \| 8% \| \| grade 1-2 \| NS \| 29% \| \| hand-foot syndrome \| grade ≥ 3 \| NS \| 5% \| \| grade 1-2 \| NS \| 28% \| \| The authors indicate that the comparable toxicity burden suggests that *1/*2A is not underexposed when treated with a median dose of 48%. \| \| \| \|  \| Dose-normalised pharmacokinetics and DPD enzyme activity for *1/*2A compared to *1/*1: \| \| \| \| --- \| --- \| --- \| \|  \|  \| value for *1/*1 \| \| 5-FU AUC normalised to a capecitabine dose of 1250 mg/m^2^ \| x 2.03 (NS) \| 602 ng.h/ml \| \| DPD enzyme activity in peripheral mononuclear blood cells \| x 0.64 (S) \| 9.9 nmol/ hr per mg protein \| | Authors’ conclusion:  ‘*DPYD**2A genotype-guided dosing results in adequate systemic drug exposure and significantly improves safety of fluoropyrimidine therapy for the individual patient. On a population level, upfront genotyping seemed cost saving.’  AUC versus gene activity 2:  gene activity 1: 203% |
| ref. 15 – FU/CAP, mono/comb  Meulendijks D et al.  Clinical relevance of *DPYD* variants  c.1679T>G, c.1236G>A/HapB3, and c.1601G>A as predictors of severe fluoropyrimidine-associated toxicity: a systematic review and meta-analysis of individual patient data.  Lancet Oncol 2015;16:1639-50. PubMed PMID: 26603945.  ref. 15, continuation  ref. 15, continuation  ref. 15, continuation  ref. 15, continuation  ref. 15, continuation  ref. 15, continuation | Level of evidence score: 4  AS 1: CTC-AE 4  AS 1.5 + PHENO: CTC-AE 4  AS 1.5: CTC-AE 4 | Meta-analysis of 8 cohort studies with in total 7365 patients treated with 5-FU or capecitabine, either as combined chemotherapy (different combinations) or as monotherapy (with or without radiotherapy).  Data on *13 were derived from 5 studies including a total of 5,616 patients and 11 carriers of *13. Data on c.1236G>A were derived from 6 studies including a total of 4,261 patients and 174 heterozygous carriers and 3 homozygous carriers of c.1236G>A. Data on *2A were derived from 7 studies including a total of 5.737 patients and 60 carriers of *2A. Data on c.2846A>T were derived from all 8 studies including a total of 7,318 patients and 85 carriers of c.2846A>T.  1 of the 8 studies in this meta-analysis is also included in the meta-analysis of Rosmarin 2014 (Rosmarin 2014). 2 of the 8 studies in this meta-analysis are also included in the meta-analysis of Terrazzino 2013 (Morel 2006 and Deenen 2011).  5 of the 8 studies in this meta-analysis are also included separately in this risk analysis: Morel 2006, Deenen 2011, Lee 2014, Rosmarin 2014 and Meulendijks 2017.  If possible, a RR was calculated for each study based on individual patient data and adjusted for age, sex, and treatment regimen. For 2 of the 5 studies for *13, it was not possible to use individual patient data. A random-effects model was used for the meta-analysis.  Haematological toxicity included thrombocytopenia, neutropenia, leukocytopenia, and anaemia. Gastro-intestinal toxicity included diarrhoea, mucositis/stomatitis, and nausea/vomiting.  Short timeframe was defined as shorter than the complete treatment duration, long timeframe as the whole treatment duration.  In addition, a meta-analysis of 3 case-control studies with in total 799 patients was performed for c.1236G>A. One of these case-control studies is also included in the meta-analysis of Rosmarin 2014 (Schwab 2008) and two in the meta-analysis of Terrazzino 2013 (Schwab 2008 and Kleibl 2009). One of these case-control studies is also included separately in this risk analysis (Schwab 2008).  Results:   \| Risk of grade ≥ 3 toxicity for *1/*13 compared to *1/*1: \| \| \| \| --- \| --- \| --- \| \|  \| RR_adj_ (95% CI) \| incidence for *1/*1 (% of patients) \| \| any toxicity \| 4.40 (2.08-9.30) (S) \| 22% \| \|  \|  \|  \| \| haematological toxicity \| 9.76 (3.03-31.48) (S) \|  \| \| gastrointestinal toxicity \| 5.72 (1.40-23.33) (S) \|  \| \| hand-foot syndrome \| - (RR could not be calculated due to an incidence of 0% in *1/*13) \|  \| \| The heterogeneity between the studies was significant and substantial, possibly because of the small number of *1/*13.  There was no indication of publication bias. \| \| \| \| The results for any toxicity were similar when patients carrying *2A and/or c.2846A>T were excluded from the meta-analysis. The association remained significant with p < 0.0167 after exclusion of any study from the meta-analysis, except for Loganayagam 2013. After exclusion of Loganayagam 2013, the p-value was 0.0433. \| \| \| \| The effect of *13 on risk of severe toxicity seemed similar in studies with long and short timeframes. \| \| \| \| The sensitivity of *13 in prediction of grade ≥ 3 toxicity was 0.3% and the positive predictive value 46%. \| \| \|  \| Risk of grade ≥ 3 toxicity for (*1/c.1236G>A + c.1236G>A/c.1236G>A) compared to *1/*1: \| \| \| \| --- \| --- \| --- \| \|  \| RR_adj_ (95% CI) \| incidence for *1/*1 (% of patients) \| \| any toxicity \| 1.59 (1.29-1.97) (S) \| 22% \| \|  \|  \|  \| \| haematological toxicity \| 2.07 (1.17-3.68) (S) \|  \| \| gastrointestinal toxicity \| 2.04 (1.49-2.78) (S) \|  \| \| hand-foot syndrome \| NS (also for the sub-group treated with capecitabine) \|  \| \| There was no significant heterogeneity between the studies.  There was no indication of publication bias. \| \| \| \| The results for any toxicity were similar when patients carrying *2A and/or c.2846A>T were excluded from the meta-analysis. The association remained significant after exclusion of any study from the meta-analysis. \| \| \| \| The effect of c.1236G>A on risk of severe toxicity seemed similar in studies with long and short timeframes. \| \| \| \| The sensitivity of c.1236G>A in prediction of grade ≥ 3 toxicity was 6.4% and the positive predictive value 41%. \| \| \| \| The meta-analysis of the case-control studies did not show a significant result, probably due to the smaller number of patients. \| \| \| \| The authors reported to have treated 3 patients with genotype c.1236G>A/c.1236G>A safely with low dose capecitabine (825 mg/m^2^ twice a day). \| \| \|  \| Risk of grade ≥ 3 toxicity for *2A-carriers compared to *1/*1: \| \| \| \| --- \| --- \| --- \| \|  \| RR_adj_ (95% CI) \| incidence for *1/*1 (% of patients) \| \| any toxicity \| 2.85 (1.75-4.62) (S) \| 29% \| \| The heterogeneity between the studies was significant and strong.  There was no indication of publication bias. \| \| \|  \| Risk of grade ≥ 3 toxicity for c.2846A>T-carriers compared to *1/*1: \| \| \| \| --- \| --- \| --- \| \|  \| RR_adj_ (95% CI) \| incidence for *1/*1 (% of patients) \| \| any toxicity \| 3.02 (2.22-4.10) (S) \| 25% \| \| The heterogeneity between the studies was significant and strong.  There was no indication of publication bias. \| \| \|   NOTE: c.1236G>A is in complete linkage disequilibrium with c.1129-5923C>G in haplotype B3. Studies analysing both gene variants were pooled.  NOTE: Meta-analysis of 5 studies with in total 3900 patients, 182x *1/*4 and 2x *4/*4, showed no significant association between *4 and grade ≥ 3 toxicity. The only study that found a significant effect (Loganayagam 2013) was the cause of strong heterogeneity between the studies. In addition, results regarding the effect of *4 on DPD activity are inconsistent. | Authors’ conclusion:  ‘*DPYD* variants c.1679T>G and c.1236G>A/HapB3 are clinically relevant predictors of fluoropyrimidine-associated toxicity. Upfront screening for these variants, in addition to the established variants *DPYD**2A and  c.2846A>T, is recommended to improve the safety of patients with cancer treated with fluoropyrimidines.’ |
| ref. 16 – FU, comb  Lee AM et al. *DPYD* variants as predictors of 5-fluorouracil toxicity in adjuvant colon cancer treatment (NCCTG N0147).  J Natl Cancer Inst 2014;106:dju298. PubMed PMID:  25381393.  ref. 16, continuation  ref. 16, continuation  ref. 16, continuation  ref. 16, continuation  ref. 16, continuation  ref. 16, continuation | Level of evidence score: 3  AS 1 + PHENO: CTC-AE 4  AS 1.5 + PHENO: CTC-AE 4  PHENO:CTC-AE 5(2)^#^ | 2594 patients were treated with 12 cycles of adjuvant FOLFOX therapy (5-fluorouracil, folinic acid and oxaliplatin; 91.9% of patients) or FOLFIRI therapy (5-fluorouracil, folinic acid and irinotecan; 8.1% of patients) with or without cetuximab. Part of the patients received 6 cycles of FOLFOX followed by six cycles of FOLFIRI with or without cetuximab. 62.0% of patients had any grade ≥ 3 adverse event, with 33.1% having any grade ≥ 3 adverse event common to 5-fluorouracil treatment.  Adverse events classified as common to 5-fluorouracil treatment were fatigue, anorexia, dehydration, diarrhoea, stomatitis/mucositis, nausea/vomiting, leukopenia, neutropenia, febrile neutropenia, thrombocytopenia, and pain. Most frequent 5-fluorouracil adverse events included diarrhoea (12.0%), neutropenia (11.7 %), nausea/vomiting (5.0%), fatigue (4.9%), and mucositis (4.2%).  Follow-up for disease free survival was for 5 years.  Results were adjusted for clinicopathological factors like age, sex, treatment, total number of treatment cycles and dose modifications. The latter two outcomes (higher percentage of patients with premature continuation and with dose modification) might be results of 5-fluorouracil adverse events instead of causes.  Cetuximab increased the risk of 5-fluorouracil adverse events. OR’s were adjusted for this, but other outcomes were not. In addition, this indicates that adverse events common to 5-fluorouracil are not the same as 5-FU-induced adverse events.  Genotyping:  - 2532x *1/*1  - 24x *1/*2A  - 26x *1/c.2846A>T  - 1x *2A/c.2846A>T  - 1x *1/274C  - 5x *2A-genotyping failed  - 5x c.2846A>T-genotyping failed    Results:   \| Risk of grade ≥ 3 toxicity, premature treatment termination and disease free survival for *2A-carriers compared to non-carriers: \| \| \| \| --- \| --- \| --- \| \|  \|  \| incidence for non-carriers \| \| any toxicity \| OR_adj_ = 3.58 (95% CI: 1.01-12.64) (S) \| 62% \| \| any 5-FU toxicity \| OR_adj_ = 14.91 (95% CI: 4.26-52.18) (S) \| 33% \| \|  \|  \|  \| \| diarrhoea \| NS \| 12% \| \| neutropenia \| x 5.7 (S) \| 11% \| \| nausea/vomiting \| x 4.2 (S) \| 4.8% \| \| fatigue \| NS \| 4.8% \| \| stomatitis/mucositis \| NS, trend for an increase, p = 0.09 \| 4.2% \| \| dehydration \| NS \| 2.3% \| \| leukopenia \| NS, trend for an increase, p = 0.08 \| 1.8% \| \| febrile neutropenia \| NS, trend for an increase, p = 0.07 \| 1.6% \| \| anorexia \| NS \| 1.5% \| \| pain \| NS \| 0.8% \| \| thrombocytopenia \| NS, trend for an increase, p = 0.08 \| 0.3% \| \| premature treatment termination \| x 1.7 (S) \| 26% \| \| dose modification \| NS \| 74% \| \|  \|  \|  \| \| disease free survival  after 3 year \| NS \| 73% \| \| When restricting the analysis to Caucasians, sex or treatment, the association between *2A and grade ≥ 3 5-FU toxicity remained significant, whereas the association between *2A and grade ≥ 3 overall toxicity did not. \| \| \|  \| Risk of grade ≥ 3 toxicity, premature treatment termination and disease free survival for *c.2846A>T-carriers compared to non-carriers: \| \| \| \| --- \| --- \| --- \| \|  \|  \| incidence for non-carriers \| \| any toxicity \| OR_adj_ = 5.43 (95% CI: 1.52-19.43) (S) \| 62% \| \| any 5-FU toxicity \| OR_adj_ = 10.24 (95% CI: 3.57-29.40) (S) \| 33% \| \|  \|  \|  \| \| diarrhoea \| x 2.8 (S) \| 12% \| \| neutropenia \| x 4.9 (S) \| 11% \| \| nausea/vomiting \| NS \| 5.0% \| \| fatigue \| NS \| 4.8% \| \| stomatitis/mucositis \| NS \| 4.1% \| \| dehydration \| x 5.0 (S) \| 2.2% \| \| leukopenia \| x 8.2 (S) \| 1.8% \| \| febrile neutropenia \| NS, trend for an increase, p = 0.08 \| 1.6% \| \| anorexia \| NS \| 1.5% \| \| pain \| NS \| 0.8% \| \| thrombocytopenia \| x 55.5 (S) \| 0.2% \| \| premature treatment termination \| NS \| 26% \| \| dose modification \| NS \| 74% \| \|  \|  \|  \| \| disease free survival after 3 year \| NS \| 73% \| \| When restricting the analysis to Caucasians, sex or treatment, the association between c.2846A>T and grade ≥ 3 5-FU toxicity remained significant. The association between c.2846A>T and grade ≥ 3 overall toxicity remained significant in the subgroups of Caucasians and males, but not in the subgroups of females, FOLFOX only and FOLFOX + cetuximab. \| \| \|  \| Other results: \| \| --- \| \| - Because of its low frequency, a statistically significant association could not be demonstrated between *13 and either 5-FU or overall grade ≥ 3 toxicity (NS). \| \| - The *2A/c.2846A>T-patient had a grade 5 adverse event. The patient was only able to receive one cycle of FOLFOX + cetuximab. \| \| - The *1/274C-patient had no grade ≥ 3 adverse events. \| \| - The gene variants *2A, *13 and c.2846A>T together predicted 5-FU grade ≥ 3 toxicity with a sensitivity of 5.3%, specificity of 99.4%, positive predictive value of 81.8% and negative predictive value of 68%. The low sensitivity and negative predictive value might be attributed to the combination chemotherapy, which may add to the 5-FU toxicity. \|   NOTE: Genotyping was for 25 gene variants of which only 4 (*2A, *13, c.2846A>T and c.274G>C) were found in this population from the USA. | Authors’ conclusion:  ‘Statistically significant associations were found between *DPYD* variants (*DPYD**2A and 2846A>T) and increased incidence of grade 3 or greater 5FU-adverse events in patients treated with adjuvant 5-FU-based combination chemotherapy.’ |
| ref. 17 – CAP/FU, comb  Rosmarin D et al. Genetic markers of toxicity from capecitabine and  other fluorouracil-based regimens: investigation in the QUASAR2 study, systematic review, and meta-analysis.  J Clin Oncol 2014;32:1031-9. PubMed PMID: 24590654.  ref. 17, continuation  ref. 17, continuation  ref. 17, continuation | Level of evidence score: 4  AS 0-1.5 + PHENO: CTC-AE 4 | After colorectal cancer excision, 927 patients received adjuvant therapy with capecitabine 1250 mg/m^2^ twice daily on days 1-14 of a 3-week cycle either as monotherapy (n = 436) or in combination with bevacizumab (n = 491). Grade III-V toxicity comprised hand-foot syndrome (n = 206), diarrhoea (n = 97) and neutropenia (n = 19).  Variant c.2846A>T:  - Associated with grade III-V toxicity (OR = 9.35; 95% CI: 2.01-43.4) (S)  - No association with grade III-V diarrhoea and grade III-V hand-foot syndrome (NS). Given the allele frequency found, this is apparently based on 5 defect alleles.  Variants *2A, c.496A>G, c.1236G>A:  - No association with grade III-V toxicity, grade III-V diarrhoea and grade III-V hand-foot syndrome (NS). Given the allele frequency found, this is apparently based on 4 defect alleles for *2A, 83 for c.496A>G and 18 for c.1236G>A.  Variant c.2846A>T and/or *2A:  - Associated with grade III-V toxicity (OR = 5.51; 95% CI: 1.95-15.5) (S)  - No association with grade III-V diarrhoea and grade III-V hand-foot syndrome (NS)  - Both patients who died were carriers of *2A or c.2846A>T  Meta-analysis of 6 studies during which Caucasian patients received capecitabine or 5-FU-based therapy. Of these 6 studies, the study covered in the paragraph above and Schwab, 2008, were also included separately in this risk analysis.  Variant *2A:  - No association with grade III-V toxicity for capecitabine (2 studies, n = 1035) (NS)  - No significant association with grade III-V toxicity for 5-FU infusion, but there was a trend (2 studies, n = 732) (NS; p = 0.0075, whilst this should be less than 0.0048 due to multiple testing)  - No significant association with grade III-V toxicity for 5-FU bolus injection, but increased risk of grade III-V neutropenia (OR = 12.9; 95% CI: 3.13-53.3) (1 study, n = 338) (S)  Variant c.2846A>T:  - No meta-analysis for capecitabine, 5-FU infusion and 5-FU bolus injection (1 study each time)  Variant c.496G>A:  - No meta-analysis for capecitabine and 5-FU infusion (in both cases only 1 study)  - No association with grade III-V toxicity for 5-FU bolus injection (2 studies, n = 379) (NS)  Variant c.1236G>A:  - No meta-analysis for capecitabine, 5-FU infusion and 5-FU bolus injection (1 study each time)  Variant c.2846A>T and/or *2A:  - No meta-analysis for capecitabine (only 1 study)  - There was a significant association (p = 0.05) with grade III-V toxicity for 5-FU infusion and 5-FU bolus injection (S)  NOTE: No association was found for the gene variants *4, *5, *6 and *9A. However, associations with severe toxicity have never been found in studies concerning these gene variants. | Authors’ conclusion:  “Global capecitabine toxicity (grades 0/1/2 v grades 3/4/5) was associated with the rare, functional *DPYD* alleles c.2846A>T>A and *2A (combined odds ratio, 5.51).” |
| ref. 18 – FU/CAP, mono/comb  Terrazzino S et al. *DPYD* IVS14+1 G>A and c.2846A>T genotyping for the prediction of severe fluoropyrimidine-related toxicity: a meta-analysis. Pharmacogenomics  2013;14:1255-72. PubMed PMID: 23930673.  ref. 18, continuation  ref. 18, continuation  ref. 18, continuation | Level of evidence score: 4  AS 1: CTC-AE 4  AS 1.5: CTC-AE 4 | Meta-analysis of 15 studies investigating patients treated with fluorouracil, capecitabine or tegafur-uracil (1 study). Data on *2A (IVS14+1G>A) were derived from 15 studies including a total of 4,094 patients and 60 carriers of *2A. Data on c.2846A>T were derived from 7 studies including a total of 2,308 patients and 34 carriers of c.2846A>T. These 15 studies include 8 studies that have also been included separately in this risk analysis: Salgueiro 2004, Morel 2006, Largillier 2006, Boisdron-Celle 2007, Schwab 2008, Sulzyc-Bielicka 2008, Kristensen 2010 and Deenen 2011.  *2A versus (no *2A):  Increased risk of grade III-V toxicity (OR = 5.42; 95% CI: 2.79-10.52; increase in the percentage of patients with grade III-V toxicity from 39% to 68%) (S) Exclusion of each of the studies from the meta-analysis did not lead to substantially different results (OR = 4.05 - 7.32 (S)). The risk was increased in studies in which the percentage of patients with grade III-V toxicity was less than 40% (OR = 8.31; 95% CI: 3.63-19.06) (S). However, the increase was non-significant in studies including ≥40% of patients with toxicity. The results were similar if only prospective studies, only higher quality studies or only studies including ≥ 200 patients were analysed. In prospective studies, the risk also increased as the incidence of grade III-V toxicity decreased in the study. The risk was also increased when only studies investigating 5-FU-based therapy or 5-FU monotherapy were analysed.  Increased risk of grade III-V haematological toxicity (OR = 15.77; 95% CI: 6.36-39.06) (S)  Increased risk of grade III-V diarrhoea (OR = 5.54; 95% CI: 2.31-13.29) (S)  Increased risk of grade III-V mucositis (OR = 7.48; 95% CI: 3.03-18.47) (S)  *2A had a sensitivity of 5.2% (95% CI: 3.0-8.9) and a specificity of 99.2% (95% CI: 98.8-99.4) for predicting grade III-V toxicity (S)  The sensitivity was 9.0% for studies that showed less than 40% grade III-V toxicity (95% CI: 5.7-13.9) (S). There was study heterogeneity in the overall group, but not in the group with less than 40% toxicity.  *2A had a sensitivity of 13% (95% CI: 6.6-24.1) for predicting grade III-V haematological toxicity (S)  *2A had a sensitivity of 5.6% (95% CI: 3.2-9.7) for predicting grade III-V diarrhoea (S)  *2A had a sensitivity of 11.5% (95% CI: 6.2-20.5) for predicting grade III-V mucositis (S)  c.2846A>T versus (no c.2846A>T):  Increased risk of grade III-V toxicity (OR = 8.18; 95% CI: 2.65-25.25; increase in the percentage of patients with grade III-V toxicity from 34% to 71%) (S) Exclusion of each of the studies from the meta-analysis did not lead to substantially different results (OR = 6.20 - 12.88 (S)). The risk was increased in studies in which the percentage of patients with grade III-V toxicity was less than 40% (OR = 16.59; 95% CI: 5.06-54.43) (S). However, the increase was non-significant in studies including ≥40% of patients with toxicity. The results were similar if higher only quality studies or only studies including ≥ 200 patients were analysed.  The risk was also increased when only prospective studies were analysed (OR = 18.14; 95% CI: 6.26-52.58) (S) or only studies investigating 5-FU-based therapy (OR = 21.38; 95% CI: 6.71-68.15) (S). There was moderate study heterogeneity in the overall group, but not in the low or high toxicity subgroups, among prospective studies or among those investigating 5-fluorouracil-based therapy. There may have been publication bias.  Increased risk of grade III-V diarrhoea (OR = 6.04; 95% CI: 1.77-20.66) (S)  c.2846A>T had a sensitivity of 5.4% (95% CI: 1.7-16.1) and a specificity of 99.1% (95% CI: 98.7-99.4) for predicting grade III-V toxicity (S)  The sensitivity was 11.2% for studies that showed less than 40% grade III-V toxicity (95% CI: 2.8-35.1) (S). There was heterogeneity between the studies.  c.2846A>T had a sensitivity of 4.6% (95% CI: 2.2-9.4) for predicting grade III-V diarrhoea (S) | Authors’ conclusion:  “The results of this meta-analysis confirm clinical validity of *DPYD* IVS14+1 G>A and 2846A>T as risk factors for the development of severe toxicities following fluoropyrimidine treatment.” |
| ref. 19 – FU/CAP, comb  Magnani E et al. Fluoropyrimidine toxicity in patients with dihydropyrimidine dehydrogenase splice site variant: the need for further revision of dose and schedule.  Intern Emerg Med 2013;8:417-23. PubMed PMID: 23585145.  ref. 19, continuation | Level of evidence score: 2  AS 1: CTC-AE 4 | 3 patients with genotype *1/*2A with gastrointestinal or head and neck tumours received 5-FU or capecitabine-based therapy (adjuvant or metastatic therapy). A 4^th^ patient with genotype *1/*2A was not given adjuvant therapy.  A 43-year-old colon cancer patient was given adjuvant therapy with capecitabine/oxaliplatin and a 50% dose of capecitabine (500 mg/m^2^ twice daily for 14 days, followed by a week-long rest period). The patient developed diarrhoea, grade 4 neutropenia and grade 3 thrombocytopenia after 19 days. The adjuvant therapy was discontinued.  A 71-year-old colon cancer patient received the same adjuvant therapy including 40% of the normal capecitabine dose (400 mg/m^2^ twice daily). After 1 day, the patient started vomiting and developed grade 3 abdominal pain. The adjuvant therapy was discontinued.  A 68-year-old patient with metastatic maxillary sinus cancer initially received 5-FU/carboplatin/folinic acid with standard-dose 5-FU (3000 mg/m^2^ continuous infusion + 400 mg/m^2^ bolus every 3 weeks). After 15 days, he developed grade 4 neutropenia and thrombocytopenia, and grade 3 sepsis and ulceration of the palate. After recovery, the treatment was restarted at 44% of the original dose (1500 mg/m^2^ by continuous infusion) and prophylactic growth factors. There was no toxicity for 2 cycles. In the third cycle, the dose was increased to 59% of the standard dose (2000 mg/m^2^ bolus) and no growth factors were given. After 14 days, the patient developed grade 4 febrile neutropenia and grade 2 anaemia. He was henceforth given non-fluoropyrimidine-based therapy.  The authors indicated that a 50% dose decrease in gene activity score 1 is not always adequate. | Authors’ conclusion:  “Our data suggest  that greater dose reductions or alternative therapies are needed for patients with DPD IVS14+1 G>A mutations.” |
| ref. 20 – FU, comb  Vulsteke C et al. Genetic variability in the multidrug resistance associated protein-1 (ABCC1/MRP1) predicts hematological toxicity in breast cancer patients receiving (neo-)adjuvant chemotherapy with 5-fluorouracil, epirubicin and cyclophosphamide (FEC).  Ann Oncol 2013;24:1513-25. PubMed PMID:  23396606.  ref. 20, continuation | Level of evidence score: 4  AS 1: Clinical Relevance Score AA | 1012 breast cancer patients received neoadjuvant/adjuvant therapy with 5-FU, epirubicin and cyclophosphamide. The 5-FU dose was 500 mg/m^2^ every 3 weeks with a maximum of 1000 mg (n=902) or 600 mg/m^2^ with a maximum of 1200 mg (n = 110).  Variant *2A (c.1905+1G>A, rs3918290):  No significant association with serious adverse events (febrile neutropenia, prolonged grade III-IV neutropenia or severe neutropenia, grade III-IV anaemia, grade III-IV thrombocytopenia or grade III-IV non-haematological toxicity) (NS)  The authors indicated that the lack of association is likely due to the fact that 5-FU toxicity is not common among breast cancer patients treated with this combination therapy. The 5-FU dose in this combination therapy is much lower than the dose in combination therapies used for colorectal cancer.  NOTE: Associations were also not found for gene variants *5 (c.1627A>G), *6 (c.2194G>A) and *9A (c.85T>C). However, associations with severe toxicity have never been found in studies concerning these gene variants. | Authors’ conclusion:  “In our study, we did not observe any association with toxicity and IVS14+1 G>A. The absence of a significant association with IVS14+1 G>A probably relates to the fact that 5-FU toxicity is not frequent in breast cancer patients treated with FEC due to a much lower 5-FU dose in breast compared with colorectal cancer patients.” |
| ref. 21 – FU, mono/ comb  van Kuilenburg AB et al. Evaluation of 5-fluorouracil pharmacokinetics in cancer patients with a c.1905+1 G>A mutation in *DPYD* by means  of a Bayesian limited sampling strategy.  Clin Pharmacokinet 2012;51:163-74. PubMed PMID: 22339448.  ref. 21, continuation | Level of evidence score: 3  AS 1: CTC-AE 5 | Clinical aspects were determined in 20 patients who had been genotyped as *1/*2A beforehand and were treated with 5-FU. Kinetics were determined in 30 *1/*2A (c.1905+1G>A) and 18 *1/*1, who received a 5-FU bolus injection of 300 mg/m^2^ and/or 450 mg/m^2^. Treatment regimens were not given.  *Clinical*  - All 7 *1/*2A receiving a standard dose of 5-FU showed grade III-V toxicity, of which 3 showed grade IV neutropenia The severe toxicity occurred in the first cycle each time and 1 patient died.  - Among 13 *1/*2A receiving low-dose 5-FU, 4 had grade III toxicity and none had grade IV toxicity The patients with grade III toxicity received on average 74% of the standard dose, and those with grade II or lower toxicity received 61% of the dose.  *Kinetics*  *1/*2A versus *1/*1:  - The 5-FU AUC increased by 52% for the 300 mg/m^2^ dose (from 6.0 to 9.1 mg.hour/L) and by 32% for the 450 mg/m^2^ dose (from 13.4 to 17.7 mg.hour/L) (S) The dose-corrected AUC increased by 32% (from 0.026 to 0.034 mg.hour/L per mg/m^2^; 45 and 25 patient/dose combinations respectively) (S). The AUC seems to be predictive of the first 2 hours after the injection and may therefore cause an underestimate for *1/ *2A. The 5-FU concentration 1 hour after injection was around the detection limit for *1/*1.  - The terminal half-life of 5-FU increased by 109% for the 300 mg/m^2^ dose (from 0.128 to 0.268 hours) and by 69% for the 450 mg/m^2^ dose (from 0.181 to 0.306 hours) (S)  - The maximum enzymatic metabolic capacity (V_max_) calculated in a multi-compartment model decreased by 46% for the 300 mg/m^2^ dose (from 1749 to 942 mg/hour) and by 34% for the 450 mg/m^2^ dose (from 1370 to 900 mg/hour) (S) | Authors’ conclusion:  “Profound differences in the elimination of 5FU could be detected between DPD-deficient patients and control patients. Furthermore, treatment of DPD-deficient patients with standard 5FU-containing chemotherapy was associated with severe (lethal) toxicity.”  Maximum clearance (V_max_ for 300 mg/m^2^) versus EM:  gene activity 1: 54%  AUC_t_ versus EM:  gene activity 1: 132% |
| ref. 22 – CAP, comb  Deenen MJ et al. Relationship between single nucleotide polymorphisms and haplotypes in *DPYD* and toxicity and efficacy of capecitabine in advanced colorectal cancer.  Clin Cancer Res 2011;17:3455-68. PubMed PMID: 21498394.  ref. 22, continuation  ref. 22, continuation  ref. 22, continuation  ref. 22, continuation | Level of evidence score: 4  AS 1: CTC-AE 5  (AS 1.5 + PHENO): CTC-AE 4  AS 1.5: CTC-AE 4 | 568 patients with advanced colorectal cancer were treated with capecitabine 1000 mg/m^2^ twice daily for 14 days every 3 weeks, in combination with oxaliplatin and bevacizumab, with or without cetuximab. Oxaliplatin was discontinued from cycle 7 and the capecitabine dose increased to 1250 mg/m^2^. Grade III-IV toxicity occurred in 85% of the patients.  *1/*2A versus *1/*1:  - Factor 3.0 increase in the percentage of patients with grade III-IV diarrhoea (from 24% to 71%) (S; strong association: false discovery rate < 0.3) The sensitivity of *2A for predicting grade III-IV diarrhoea was 4% and the specificity 100%.  - No increase in the percentage of patients with grade II-III hand-foot syndrome and no significant increase in the percentage of patients with grade III-IV toxicity (NS) All 7 *1/*2A developed grade III-IV toxicity (including 3 women), and 1 patient died during the 3^rd^ cycle.  - Decrease in the cumulative dose over the first 6 cycles (S): the average dose decrease increased from 10% to 51% in the lowest-dose cycle and from 10% to 44% in cycle 6.  - No difference in mortality or progression-free survival (NS)  (*1/c.1236G>A + c.1236G>A/c.1236G>A) versus *1/*1:  - Factor 2.2 increase in the percentage of patients with grade III-IV diarrhoea (from 23% to 50%) (S; strong association: false discovery rate < 0.3) The sensitivity of c.1236G>A for predicting grade III-IV diarrhoea was 10% and the specificity 97%.  - No significant increase in the percentage of patients with grade II-III hand-foot syndrome or with grade III-IV toxicity (NS).  - No significant increase in dose decreases (NS)  - No difference in mortality or progression-free survival (NS)  *1/c.2846A>T versus *1/*1:  - Factor 2.6 increase in the percentage of patients with grade III-IV diarrhoea (from 24% to 62%) (S; medium association: false discovery rate 0.3-0.4) The sensitivity of c.2846A>T for predicting grade III-IV diarrhoea was 4% and the specificity 99%.  - No significant increase in the percentage of patients with grade II-III hand-foot syndrome or with grade III-IV toxicity (NS).  - Decrease in the cumulative dose over the first 6 cycles (S): the average dose decrease increased from 10% to 27% in the lowest-dose cycle and from 10% to 24% in cycle 6.  - No difference in mortality or progression-free survival (NS)  (*1/*6 + *6/*6) versus *1/*1:  - Factor 1.8 increase in the percentage of patients with grade III-IV diarrhoea (from 23% to 41%) (S; medium association: false discovery rate 0.3-0.4) The sensitivity of *6 (c.2194G>A) for predicting grade III-IV diarrhoea was 12% and the specificity 95%.  - No significant increase in the percentage of patients with grade II-III hand-foot syndrome or with grade III-IV toxicity (NS).  - No significant increase in dose decreases (NS)  - No difference in mortality or progression-free survival (NS)  (*1/c.496A>G + c.496A>G/c.496A>G) versus *1/*1:  - Factor 1.4 increase in the percentage of patients with grade III-IV diarrhoea (from 23% to 33%) (S; weak association: false discovery rate < 0.3) The sensitivity of c.496A>G for predicting grade III-IV diarrhoea was 24% and the specificity 84%.  - Factor 1.3 increase in the percentage of patients with grade II-III hand-foot syndrome (from 41% to 53%) (S; weak association: false discovery rate < 0.3) The sensitivity of c.496A>G for predicting grade II-III hand-foot syndrome was 22% and the specificity 85%.  - No significant increase in the percentage of patients with grade III-IV toxicity (NS).  - No significant increase in dose decreases (NS)  - No difference in mortality or progression-free survival (NS)  *13:  - The percentage *1/*13 was 0% among 43 patients with grade IV-V toxicity or two forms of grade III-V toxicity and 1% in 99 randomly selected patients (NS)  The authors indicated that the lack of association with grade III-IV toxicity for each of the investigated SNPs is likely caused by the high risk in the overall population.  NOTE: No associations were found for gene variants *4 (c.1601G>A), *5 (c.1627A>G) and *9A (c.85T>C). However, associations with severe toxicity have never been found in studies concerning these gene variants. | Authors’ conclusion:  “Of the patients polymorphic for *DPYD* IVS14+1G>A, c.2846A>T, and c.1236G>A, 71% (5 of 7), 63% (5 of 8), and 50% (14 of 28) developed grade 3 to 4 diarrhoea, respectively, compared with 24% in the overall population.  ……  *DPYD* IVS14+1G>A and 2846A>T predict for severe toxicity to capecitabine, for which patients require dose reductions.  …..  The data suggest that initial dose reductions of 50% in IVS14+1 G>A and 25% in c.2846A>T variant allele carriers with further dose titration would significantly reduce the total number of severe toxicity events, thereby separate validation is indicated.” |
| ref. 23 – FU/CAP, mono/comb  Kristensen MH et al.  Variants in the  dihydropyrimidine dehydrogenase, methylenetetrahydrofolate reductase and thymidylate synthase genes predict early toxicity of 5-fluorouracil in colorectal cancer patients.  J Int Med Res 2010;38:870-83. PubMed PMID: 20819423. | Level of evidence score: 3  AS 1.5: CTC-AE 4 | 68 patients with advanced colorectal cancer were given adjuvant or palliative treatment with fluoropyrimidine-based therapy. Therapy consisted of either a 5-FU bolus injection 500 mg/m^2^ every 2 weeks plus folinic acid (n=24) or fluorouracil (400 mg/m^2^ bolus plus 600 mg/m^2^ by infusion every 2 weeks) plus folinic acid and oxaliplatin (n=27) or capecitabine 1250 mg/m^2^ twice daily for 14 days every 3 weeks (n=17). There was no significant difference between incidences of grade I-IV toxicity in the first 2 cycles caused by the different chemotherapies. However, the proportion of grade III-IV toxicity did differ (67%, 33% and 0% respectively).  Results:  - Higher frequency of c.1896C>T in the group with grade I-IV toxicity than in the group without toxicity (13% versus 2% c.1896C>T heterozygotes; there were no homozygotes; RR = 6) (S)  - Of the 4 c.1896C>T heterozygotes, 2 developed grade III-IV toxicity, 1 developed grade I toxicity and 1 did not develop toxicity; the number of patients with toxicity was 24, the number of patients without was 44. This is equivalent to 8.3% c.1896C>T heterozygotes in the group with grade III-IV toxicity and 4.5% in the group with < grade III toxicity. This is equivalent to an RR of 1.8 for grade III-IV toxicity. | Authors’ conclusion:  “Patients with the genetic variant  IVS14+1 G/A or c1896 C/T in the *DPYD* gene had a statistically significant increased risk of experiencing toxicity (RR 2 and 6, respectively), both having a high specificity (0.97 and 0.98, respectively) and low sensitivity (0.04 and 0.13, respectively). It is concluded that pre-treatment detection of genetic variants can help to predict early toxicity experienced by patients receiving 5-FU-based chemotherapy.” |
| ref. 24 – FU/CAP, comb  Gross E et al.  Strong association of a common dihydropyrimidine dehydrogenase gene polymorphism with fluoropyrimidine-related toxicity in cancer patients.  PLoS ONE 2008;3:e4003.  ref. 24, continuation | Level of evidence score: 3  AS 1.5: CTC-AE 5  AS 1: CTC-AE 4 | 128 Caucasian patients including 39 with poor tolerance to FU combination therapy (grade III or IV toxicity). 2 of the patients with poor tolerance died as a result of FU-associated toxicity. Independent group of 53 patients with poor tolerance to FU (n=39) or capecitabine combination therapy (n=14). The presence of variants was investigated by fully sequencing the DPD alleles.  Variant c.496A>G:  Strongest association with grade III and IV toxicity: OR = 4.42 [95% CI = 2.12-9.23] for 92 patients with toxicity.  The polymorphism attributable risk was 56.9%.  The association was significant in patients with breast and gastro-oesophageal cancer (n=56 and n=158), but was non-significant in colon cancer patients n=128).  1 of the fatalities was heterozygous.  All 3 homozygotes had grade III or IV toxicity.  Grade III and IV toxicity (especially diarrhoea and hand-foot syndrome) also occurred in carriers using capecitabine-based chemotherapy. Chemotherapy was discontinued in 2 of these.  The association seems stronger with combination therapy than with monotherapy.  Variant c.1129-15T>C (IVS10-15T>C):  Association with grade III and IV toxicity: OR = 3.38 [95% CI = 1.71-8.78] for 39 patients with toxicity.  The association was significant in patients with breast and gastro-oesophageal cancer (n=46 and n=146), but was non-significant in colon cancer patients (n=58).  Variant *2A (IVS14+1G>A):  Low allele frequency in these groups (0.03 in patients with severe toxicity; 0 in healthy people and patients without severe toxicity) (NS difference).  16 other variants identified:  No significant association with severe toxicity. | Authors’ conclusion:  “Our results show compelling evidence that, at least in distinct tumour types, a common *DPYD* polymorphism strongly contributes to the occurrence of fluoropyrimidine-related drug adverse events. Carriers of this variant could benefit from individual dose adjustment of the fluoropyrimidine drug or alternate therapies.” |
| ref. 25 – FU, mono  Capitain O et al.  The influence of fluorouracil outcome parameters on tolerance and efficacy in patients with advanced colorectal cancer.  Pharmacogenomics J 2008;8:256-67. | Level of evidence score: 3  (AS 1-1.5):CTC-AE 4(2)^#^ | 76 French patients with advanced colon cancer received weekly or two-weekly FU plus folinic acid (initial FU dose 1200 and 2500 mg/m^2^ respectively; by continuous infusion, two-weekly regimen partially using a bolus (400 mg/m^2^); dose adjustments based on a target AUC of 25 mg.h/L; dose reduction of 10% in the event of significant grade II toxicity, discontinuation and dose decrease of 25% in the event of grade III toxicity and discontinuation of therapy in the event of grade IV toxicity), screening for *2A (IVS14+1G>A), c.2846A>T, *13 (c.1679T>G) and c.464T>A and for DPD-deficient patients and also for 19 other variants.  *-* 11.8% of the patients (n=9) displayed abnormally low clearance of FU associated with abnormal dihydrouracil/uracil plasma ratio prior to therapy. An SNP was found in 3 of these (2x c.2846A>T, 1x *2A).  *-* Despite pharmacological dose adjustments, the incidence of grade III and IV toxicity was higher in the group with reduced DPD activity (n=9) than in the group with normal DPD activity (33.3% versus 7.5%; S by 347%; OR = 6.20 [95% CI = 1.18-32.56]).  *-* The incidence of grade III and IV toxicity was higher in the group with SNPs (n=3) than in the group without SNPs (66.7% versus 8.2%; S by 711%).  *-* The authors indicated that the increased toxicity in DPD-deficient patients may have been prevented by reduced initial doses followed by pharmacokinetic dose adjustments. | Authors’ conclusion:  “Toxicity was linked to low UH2/U ratio, c.2846 A>T, IVS14+1 G>A for DPD.” |
| ref. 26 – FU  Sulzyc-Bielicka V et al.  5-Fluorouracil toxicity-attributable IVS14 + 1G > A mutation of the dihydropyrimidine dehydrogenase gene in Polish colorectal cancer patients.  Pharmacol Rep 2008;60:238-42. | Level of evidence score: 3  AS 1:CTC-AE 4(2)^#^ | 252 Polish colon cancer patients received FU chemotherapy and screening for *2A (IVS14+1G>A).  - 1 patient was heterozygous. This patient was 1 of the 4 patients with grade III-IV neutropenia. | Authors’ conclusion:  “We conclude that IVS14 + 1G > A *DPYD* (*DPYD**2A) variant occurs in the Polish population and is responsible for a significant proportion of life-threatening toxicity of 5-FU.” |
| ref. 27 – FU, mono  Schwab M et al.  Role of genetic and nongenetic factors for fluorouracil treatment-related severe toxicity: a prospective clinical trial by the German 5-FU Toxicity Study Group.  J Clin Oncol 2008;26:2131-8.  ref. 27, continuation | Level of evidence score: 3  AS 1: CTC-AE 4 | 683 German patients (670x *1/*1, 13x *1/*2A), of whom 110 with grade III/IV toxicity; FU monotherapy with folinic acid or levamisole; screening for *2A (IVS14+1G>A) and also sequencing of exons and exon/intron transitions in 28 patients with grade IV toxicity, grade III toxicity or grade 0-II toxicity.  *1/*2A versus *1/*1:  Increased risk of grade III/IV toxicity: OR = 4.67 [95% CI = 1.54-14.2].  Significantly increased risk of grade III/IV leukopenia and mucositis (OR = 10.19 [95% CI = 3.0-35.1] and OR = 5.8 [95% CI = 1.71-19.4] respectively), but not of grade III/IV diarrhoea.  Significantly increased risk of grade III/IV toxicity in men (OR = 41.8 [95% CI = 9.2-190]), but not in women.  The sensitivity of *2A genotyping for overall toxicity was 5.5% [95% CI = 0.02-0.11] with a positive predictive value of 0.46 [95% CI = 0.19-0.75].  Sequencing of 3x 28 patients with different toxicity classes:  12 additional SNPs, including 4 new ones.  5 variants (c.623G>A, *4 (c.1601G>A), *6 (c.2194G>A), c.2846A>T and c.2585G>C) further investigated in ≥ 250 patients.  c.2585G>C was found in 1 patient with grade IV mucositis, but not in other patients (NS).  The percentage of patients with toxicity was increased for c.2846A>T (60% versus 16.1% in the overall population) (NS).  All other variants did not show a significant association with toxicity.  Inclusion of the additional variants only led to a marginal improvement in the prediction of overall toxicity.  The method of administration is an independent risk factor: the risk of grade III/IV toxicity was greater for the bolus Mayo regimen than for the high-dose infusion (OR=2.44 [95% CI 1.52-3.91]). | Authors’ conclusion:  “*DPYD*, TYMS, and MTHFR play a limited role for FU related toxicity but a pronounced *DPYD*  gene/sex-interaction increases prediction rate for male patients.” |
| ref. 28 – FU, comb  Mercier C et al. Prospective phenotypic screening for DPD deficiency prior to 5-FU administration:  decrease in toxicity, not in efficacy.  J Clin Oncol 2008;26(May 20 suppl):abstr 14556.  (meeting abstract) | Level of evidence score: 3 | 59 French patients with inoperable head and neck cancer; determination of DPD activity (dihydrouracil/uracil ratio) prior to FU combination therapy or radio-chemotherapy; mild DPD deficiency (dihydrouracil/uracil ratio < 0.5): FU dose was 80% of the standard dose, severe DPD deficiency (ratio < 0.33): FU dose was 50% of the standard dose, complete DPD deficiency: no FU.  - 25% of the patients had mild and 22% severe DPD deficiency.  - 12% of the patients with DPD deficiency and dose reduction showed severe toxicity. The incidence of severe toxicity was twofold lower in the overall group compared to the regimen without dose reduction.  - There were no toxicity-induced fatalities.  - The effectiveness was similar to the regimen without dose reduction (percentages of responders 64% and 81% for first-line chemotherapy and radio-chemotherapy and 50% and 38% for treatment for relapsed cancer). | Authors’ conclusion:  “5-FU dose tailoring based upon DPD status evaluation led to 2 fold decrease in occurrence of severe toxicities without impairing efficacy.” |
| ref. 29 – FU, comb  Jatoi A et al.  Paclitaxel, carboplatin, 5-fluoroura-cil, and radiation for locally advanced esophageal cancer: phase II results of preliminary pharmacologic and molecular efforts to mitigate toxicity and predict outcomes: North Central Cancer Treatment Group (N0044).  Am J Clin Oncol 2007;30:507-13. | Level of evidence score: 3  AS 2 + AS 1: Clinical Relevance Score: AA | 50 American patients with locally advanced oesophageal cancer (11x *1/*1, 1x *1/*2A, 16x *1/*5, 3x *1/*6, 13x *1/*9A, 4x *9A/*9A, 1x *5/*5) participating in a phase II study received FU 225 mg/m^2^ per day by continuous infusion in combination with carboplatin, paclitaxel and radiotherapy; FU was temporarily discontinued in the event of FU-related grade III-IV toxicity, after which the dose was decreased by 20%; patients received median 81% and 66% of the standard FU dose during 1 and 2 cycles respectively; screening for *2A (IVS14+1G>A), *5 (c.1627A>G), *6 (c.2194G>A) and *9A (c.85T>C).  - Almost all patients (94%) had at least 1 incident of grade III-IV toxicity, including 3 fatalities.  - No significant associations of the polymorphisms with pathological complete response, time to progression/relapse of cancer, overall survival or grade III/IV toxicity.  NB: *5, *6 and *9A do not have reduced DPD activity. | Authors’ conclusion:  “Genotyping for polymorphisms  of dihydropyrimidine dehydrogenase, cytochrome P3A4, and glutathione-S-transferase did not predict tumour response or serious adverse events.” |
| ref. 30 – FU, comb  Magné N et al.  Dihydropyrimidine dehydrogenase activity and the IVS14+1G>A mutation in patients developing 5FU-related toxicity.  Br J Clin Pharmacol  2007;64:237-40. | Level of evidence score: 3  AS 1:CTC-AE 4(2)^#^ | 131 French patients with poor tolerance to FU combination or monotherapy (grade II neurotoxicity or grade III-IV toxicity), including 9 fatalities, and 185 unselected patients; screening for DPD activity in peripheral mononuclear blood cells and for *2A (IVS14+1G>A).  - 81% of the toxicity occurred during the 1^st^ cycle of FU chemotherapy.  - Inverse association between DPD activity and toxicity score (sum of the different toxicity grades per patient) (S).  - Percentage of patients with clear or severe DPD deficiency was higher in the case group than in the control group (17% versus 2.7% and 6% versus 0% respectively).  - Inverse association between lethal toxicity and DPD activity (S).  - Inverse association between the severity of the individual types of toxicity (grade II central neurotoxicity; grade IV mucositis, diarrhoea, neutropenia or thrombocytopenia) and DPD activity (all five S). Median DPD activity was 1.6-3.2x lower in patients with severe toxicity.  - Only 2 in 93 screened cases (2.2%) had *2A (both *1/*2A). Both had low DPD activity and high toxicity scores during the 1^st^ cycle. Neither died. | Authors’ conclusion:  “Present data suggest that IVS14+1 mutation screening has limited effectiveness in identifying patients at risk for severe 5FU toxicity.” |
| ref. 31 - FU/CAP, mono  Saif MW et al.  Dihydropyrimidine dehydrogenase deficiency (GPD) in GI malignancies: experience of 4-years.  Pak J Med Sci Q 2007;23:832-9. | Level of evidence score: 2  AS 1: CTC-AE 4 | 23 patients with excessive toxicity on FU (n=8) or capecitabine therapy (n=15), including 16 Caucasians, 3 Afro-Americans and 3 South-Asians; screening for DPD activity in peripheral mononuclear blood cells and by genotyping.  *-* 30% of the patients had DPD deficiency (n=7), including 3 who were treated with FU (500 mg/m^2^ per week or 425 mg/m^2^ per week) and folinic acid, 2 who were treated with capecitabine 1800 mg/m^2^ and 2 who were treated with high-dose bolus FU (1400 mg/m^2^) in combination with the uridine prodrug 2’,3’,5’-tri-O-acetyluridine. The deficiency was confirmed by genotyping in 1 patient: he was *1/*2A.  - 28% of the DPD-deficient patients died due to toxicity (n=2), including 1 to capecitabine and 1 to high-dose bolus FU.  - Re-challenge with capecitabine of a patient treated with FU/ folinic acid led to grade III hand-foot syndrome. | Authors’ conclusion:  “Screening patients for DPD deficiency prior to administration of 5-FU or capecitabine using 2-13C uracil breath test could potentially lower risk of toxicity.” |
| ref. 32 – FU, mono  Boisdron-Celle M et al.  5-Fluorouracil-related severe toxicity: a comparison of different methods for the pretherapeutic detection of dihydropyrimidine dehydrogenase deficiency.  Cancer Lett 2007;249:271-82.  ref. 32, continuation  ref. 32, continuation | Level of evidence score: 3  AS 1: CTC-AE 4  AS 1.5: CTC-AE 4  PHENO: CTC-AE 5(2)^#^ | 252 French patients with advanced colon cancer (163x *1/*1, 6x *1/c.2846A>T, 1x *9A/c.2846A>T, 1x *1/*2A, 1x c.-1590T>C/*2A, 1x *2A/c.2846A>T+c.85T>C, 1x *1/c.-1590T>C, 67x *1/*9A, 1x c.-1590T>C/*9A, 10x *9A/*9A*)* received either FU 400 mg/m^2^ bolus + 2500 mg/m^2^ by 46-hour infusion every 2 weeks (n=168) or FU 1200 mg/m^2^ by 4-hour infusion per week (n=84) (both regimens: plus folinic acid); dose adjustment from the second cycle based on the FU plasma concentration at the end of the previous infusion (C_ss_); discontinuation of treatment in the event of grade IV toxicity; screening for *2A (IVS14+1G>A), c.2846A>T, *7 (c.299_302del), c.1156G>T, *9A (c.85T>C), *9B (c.2657G>A), *10 (c.2983G>T), c.-1590T>C.  (*1/*2A + c.-1590T>C/*2A) versus *1/*1:  Clearance decreased by 80% (S; from 104.7 to 21.22 L/h per m^2^)  Increase in the percentage of patients with grade III-IV toxicity by 793% (S; from 5.6% to 50.0%).  (*1/c.2846A>T + 1x *9A/c.2846A>T) versus *1/*1:  Clearance decreased by 40% and 58% for the two-weekly and weekly regimens respectively (both S; from 136.0 to 81.2 L/h per m^2^ and from 104.7 to 43.9 L/h per m^2^).  Increase in the percentage of patients with grade III-IV toxicity by 1175% (S; from 5.6% to 71.4%).  *2A/c.2846A>T+c.85T>C versus *1/*1:  Clearance decreased to almost 0 (NS; by almost 100%).  Increase in the percentage of patients with grade III-IV toxicity by 1686% (NS; from 5.6% to 100%).  The patient had grade IV multi-organ toxicity and died after 40 days in Intensive Care.  (1x *9A + 2x *9A) versus *1/*1:  No difference in clearance and incidence of toxicity (NS).  1x c.-1590T>C versus *1/*1:  No difference in clearance and incidence of toxicity (NS).  Analysis of relevant SNPs had a high specificity (98.3%), but a low sensitivity (47.1%) for detecting DPD deficiency. | Authors’ conclusion:  “Except in cases where alternative treatment is recommended because the 5-FU metabolism is close to zero, IVS14 + 1G>A or 2846A>T heterozygote are not strict contra-indications to 5-FU treatment, provided that the physician is aware of it and that added precautions are taken, such as an initial 5-FU dose reduction and an individual dose adjustment based on a close clinical and pharmacokinetic follow-up.”  “In the case of a homozygous status for a relevant SNP, with a uracil plasma level higher than 100 lg/L or a UH2/U ratio below 1, then fluoropyrimidine administration must be discussed and an alternative treatment proposed.”  Clearance versus AS 2:  AS 1.5: 55%  AS 1: 20%  PHENO: almost 0% |
| ref. 27 – FU, mono  Cho HJ et al.  Thymidylate synthase (TYMS) and dihydropyrimidine dehydrogenase (*DPYD*) polymorphisms in the Korean population for prediction of 5-fluorouracil-associated toxicity.  Ther Drug Monit 2007;29:190-6. | Level of evidence score: 3  AS 1.5: Clinical Relevance Score: AA  AS 1: Clinical Relevance Score: AA | 21 Korean colon cancer patients with grade III-IV toxicity on FU therapy (500 mg/m^2^ by continuous infusion on days 1-5, plus folinic acid) and 100 healthy volunteers; screening by sequencing all exons and flanking introns.  - Very common variants (allele frequency 14-22%) in this Korean group were *5, c.1737T>C and c.1896T>C. No *2A was found.  - The percentage of patients without SNPs was similar to that in healthy volunteers (9.5% versus 10%).  - There was no significant correlation between specific genotypes and toxic response.  NB: *5 does not have reduced DPD activity. | Authors’ conclusion:  “The findings, from Korean patients with colon cancer, suggest that polymorphisms of the *DPYD* gene are not associated with an in-creased risk for toxic response to 5-FU.” |
| ref. 34 – CAP, comb  Salgado J et al.  Polymorphisms in the thymidylate sy35hase and dihydropyrimidine dehydrogenase genes predict response and toxicity to capecitabine-raltitrexed in colorectal cancer.  Oncol Rep 2007;17:325-8. | Level of evidence score: 3  AS 1:CTC-AE 4(2)^#^ | 58 Spanish patients with advanced colon cancer received capecitabine (1000 mg/m^2^ twice daily for 14 days) and raltitrexed every 3 weeks; screening for *2A (IVS14+1G>A).  1 patient was *1/*2A. This patient developed severe toxicity after the first cycle, after which FU was discontinued and more appropriate chemotherapy was started. | Authors’ conclusion:  “Considering the  common use of fluoropyrimidines, genetic screening would be highly recommendable for the presence of the DPD gene mutation (IVS14+1G>A) related to toxicity, prior to 5-FU administration.” |
| ref. 35 – FU, comb  Morel A et al.  Clinical relevance of different dihydropyrimidine dehydrogenase gene single nucleotide polymorphisms on 5-fluorouracil tolerance.  Mol Cancer Ther 2006;5:2895-904.  ref. 35, continuation  ref. 35, continuation | Level of evidence score: 3  AS 0-1.5: CTC-AE 4  AS 1:CTC-AE 5(2)^#^  AS 0:CTC-AE 4(2)^#^ | 487 French patients (300x *1/*1*,* 10x *1/c.2846A>T, 8x *1/*2A, 1x c.-1590T>C/*2A, 1x *2A/*2A, 6x *1/c.-1590T>C, 144x *1/*9A, 15x *9A/*9A, 1x *1/*13*)* received FU monotherapy (n=168) or one of 4 different FU combination therapies (n=319); dose adjustment from the second cycle based on the FU plasma concentration at the end of the previous infusion (C_ss_); discontinuation of treatment or continuation with individual dose adjustment in the event of grade III/IV toxicity; screening for 22 relevant SNPs, including 9 in all patients *2A (IVS14+1G>A), c.2846A>T, *7 (c.299_302del), c.1156G>T, *9A (c.85T>C), *9B (c.2657G>A), *10 (c.2983G>T), c.-1590T>C and *13 (c.1679T>G)) in 171 patients with or without toxicity. 5 variants were found in the population.  (*1/*2A + *2A/*2A + *1/c.2846A>T + *1/*13) versus *1/*1:  Clearance decreased by 43% (S; from 132.3 to 74.9 L/h per m^2^)  Increase in the percentage of patients with grade III-IV toxicity by 838% (S; from 6.6% to 61.9%).  One *1/*2A patient died due to toxicity.  The *2A/*2A patient developed grade IV diarrhoea, neutropenia and mucositis a few days after initiation of low-dose bolus FU in combination with epirubicin and cyclophosphamide. She was treated in Intensive Care for 15 days.  Patients with SNPs: treatment was discontinued in 40% of the patients with severe toxicity and continued with a 25-50% dose reduction and pharmacokinetic follow-up in the other 60%.  (*1/*2A + *1/*13) versus *1/*1:  Clearance decreased by 54% (NS; from 132.5 to 60.8 L/h per m^2^)  *1/c.2846A>T versus *1/*1:  Clearance decreased by 45% (NS; from 132.5 to 72.3 L/h per m^2^)  (*1/*9A + *9A/*9A + *1/c.-1590T>C) versus *1/*1:  No difference in clearance (NS, increased by 3%).  No significant difference in the percentage of patients with grade III-IV toxicity (NS).  None of the homozygous patients had grade III/IV toxicity.  The sensitivity and specificity of the analysis of the 3 most important SNPs for predicting toxicity were 0.31 and 0.98 respectively. | Authors’ conclusion:  “Pretreatment detection of three *DPYD* SNPs could help to avoid serious toxic adverse events. This approach is suitable for clinical practice and should be compared or combined with pharmacologic approaches. In the case of dihydropyrimidine dehydrogenase deficiency, 5-FU administration often can be safely continued with an individual dose adjustment.”  Clearance versus AS 2:  AS 1.5: 55%  AS1: 46% |
| ref. 36 – CAP, mono  Largillier R et al.  Pharmacogenetics of capecitabine in advanced breast cancer patients.  Clin Cancer Res 2006;12:5496-502. | Level of evidence score: 3  AS 1:CTC-AE 5(2)^#^ | 105 French patients with advanced breast cancer received capecitabine monotherapy; screening for *2A (IVS14+1G>A).  1 patient was *1/*2A. This patient died due to haematological toxicity after treatment with capecitabine 1820 mg/m^2^ per day for 12 days. | Authors’ conclusion:  “Our case report clearly identifies DPD deficiency as a source of life-threatening toxicity under capecitabine treatment.” |
| ref. 37 – FU, mono  Salgueiro N et al.  Mutations in exon 14 of dihydropyrimidine dehydrogenase and 5-fluorouracil toxicity in Portuguese colorectal cancer patients.  Genet Med 2004;6:102-7. | Level of evidence score: 3  AS 1: CTC-AE 4 | 73 Portuguese colon cancer patients (71x *1/*1, 1x *1/*2A, 1x *1/c.1845G>T), including 8 with grade III/IV toxicity; various FU regimens; sequencing of exon 14.  SNPs in exon 14 (n=2) versus no SNPs in exon 14:  Increase in the percentage of patients with grade III-IV toxicity by 1076% (S; from 8.5% to 100%). | Authors’ conclusion:  “We conclude that mutations in exon 14 of *DPYD* gene are responsible for a significant proportion of life-threatening toxicity to 5-FU, and should therefore be excluded before its administration to cancer patients.” |
| ref. 38 – FU  Van Kuilenburg AB et al.  High prevalence of the IVS14 + 1G>A mutation in the dihydropyrimidine dehydrogenase gene of patients with severe 5-fluorouracil-associated toxicity.  Pharmacogenetics 2002;12:555-8. | Level of evidence score: 3  AS 1 + AS 0): CTC-AE 4 | 60 Dutch patients with grade III/IV toxicity on FU therapy (43x *1/*1, 16x *1/*2A, 1x *2A/*2A) and 54 controls, including 35 cancer patients; screening for DPD activity in peripheral mononuclear blood cells and for *2A.  *-* 60% of the cases had reduced DPD activity (< 70% of the average activity in controls).  - 29% of the cases had 1 or 2 *2A alleles.  - Significantly higher *2A allele frequency in the cases than in the general population (S; increase by 1548% from 0.91% to 15%). | Authors’ conclusion:  “Our study demonstrates that a DPD deficiency is the major determinant of 5FU-associated toxicity. The apparently high prevalence of the IVS14 + 1G>A mutation warrants genetic screening for this mutation in cancer patients before the administration of 5FU.” |
| ref. 39 – FU, mono  Raida M et al.  Prevalence of a common point mutation in the dihydropyrimidine dehydrogenase (DPD) gene within the 5'-splice donor site of intron 14 in patients with severe 5-fluorouracil (5-FU)-related toxicity compared with controls.  Clin Cancer Res 2001;7:2832-9. | Level of evidence score: 3  AS 1:CTC-AE 5(2)^#^  AS 0:CTC-AE 5(2)^#^ | 25 German patients (19x *1/*1, 5x *1/*2A, 1x *2A/*2A) with grade III/IV toxicity on FU monotherapy (n=20), FU chemo-radiotherapy (n=2) or FU combination therapy (n=3) and 851 controls, including 800 cancer patients; screening for *2A.  - 24% of the cases had 1 or 2 *2A alleles.  - Higher *2A allele frequency in the cases than in the controls (NS; increase by 2879% from 0.47% to 14%).  - The homozygous patient and two heterozygous patients died due to toxicity. | Authors’ conclusion:  “Routine screening for the exon 14-skipping mutation and subsequent individual determination of the 5-FU pharmacokinetics of heterozygous patients provides a concept of individualized therapy and allows the avoidance of undesired treatment toxicity.” |
| ref. 40 – FU, comb  Yamaguchi K et al.  Germline mutation of dihydropyrimidine dehydrogenase gene among a Japanese population in relation to toxicity to 5-fluorouracil.  Jpn J Cancer Res 2001;92:337-42. | Level of evidence score: 3  (AS 2 + AS 1.5): Clinical Relevance Score: AA | 69 Japanese patients (61x *1/*1, 4x *1/*9A; 1x *1/*5; 1x *1/c.74A>G, 1x *1/c.812delT, 1x *1/c.1714C>G); FU combination therapy or monotherapy (FU: either 800 mg/m^2^ by 1-hour infusion or 500 mg/m^2^ per day on days 1 and 5 by continuous infusion); screening by PCR and sequencing.  *-* The percentage of patients with grade III/IV toxicity was lower among the 8 heterozygous patients than among the *1/*1 patients (NS; decrease by 18% to 0%).  NB: *5 and *9A do not have reduced DPD activity. | Authors’ conclusion:  “Our observations of  Japanese patients implied that the heterozygote is not associated with increased toxic response to 5FU.” |
| ref. 41 – FU  van Kuilenburg AB et al.  Clinical implications of dihydropyrimidine dehydrogenase (DPD) deficiency in patients with severe 5-fluorouracil-associated toxicity: identification of new mutations in the DPD gene.  Clin Cancer Res 2000;6:4705-12. | Level of evidence score: 3  (AS 1.5 + AS 1): CTC-AE 4 | 37 Dutch patients with grade III/IV toxicity on FU therapy and 22 controls; sequencing of introns and intron-exon transitions.  *-* 59% of the cases had reduced DPD activity (< 70% of the average activity in controls).  - Weak but significant correlation between DPD activity and time to toxicity.  - Higher prevalence of grade IV neutropenia in patients with reduced DPD activity compared to those with normal DPD activity (S; increased by 323%, from 13% to 55%). No higher prevalence of other types of toxicity.  - 79% of 14 patients with reduced DPD activity had 1 or 2 allele variants (3x *1/*1, 4x *1/*2A, 1x *2A/*9A, 1x *2A/*5, 1x *9A/c.496A>G, 1x *9A/c.496A>G/c.2846A>T, 1x *1/*5, 1x *5/*9A, 1x *6/*6).  NB: *5, *6 and *9A do not have reduced DPD activity. | Authors’ conclusion:  “Our results demonstrated that at least 57% (8 of 14) of the patients with a reduced DPD activity have a molecular basis for their deficient phenotype.” |
| ref. 42 – FU, cutaneous  Johnson MR et al.  Life-threatening toxicity in a dihydropyrimidine dehydrogenase-deficient patient after treatment with topical 5-fluorouracil.  Clin Cancer Res 1999;5:2006-11. | Level of evidence score: 2  AS 0: CTC-AE 3 | A 76-year-old white man developed severe stomatitis, severe inflammatory colitis, erythematous rash, neutropenia 0.6x10^9^/L and thrombocytopenia 57x10^9^/L one week after initiation of 5% FU cream twice daily on the scalp for the treatment of basal cell cancer. FU was discontinued and the patient made a gradual recovery over 3 weeks.  The patient was *2A/*2A and had no detectable DPD enzyme activity in peripheral mononuclear blood cells.  Assuming 10% cutaneous absorption, the authors estimate that application of 2 g of 5% FU cream leads to a total absorbed dose of ~20 mg/day (~0.33 mg/kg for this patient). This is much lower than the IV bolus FU dose of 500-550 mg/kg that is generally used for chemotherapy. | Authors’ conclusion:  “This study represents the first characterization of a DPD deficient patient who developed life-threatening toxicity after exposure to topical 5-FU. Considering the previously reported low cutaneous absorption rate (~10%) of topical 5-FU, we suggest that life-threatening toxicity in the population of patients receiving topical 5-FU will be limited to profoundly DPD-deficient patients (no measurable DPD enzyme activity).” |
| ref. 43 – FU  SmPC Fluorouracil PCH 15-10-12. | Level of evidence score: 0  AS 0-1.5 + PHENO: CTC-AE 4 | Warning: There have been reports of increased 5-FU toxicity in patients with partially functional or non-functional dihydropyrimidine dehydrogenase (DPD). If appropriate, DPD enzyme activity should be determined prior to treatment with 5-fluoropyrimidines. |  |
| ref. 44 – FU  SmPC Efudix (5-fluorouracil) crème 08-10-18.  ref. 44, continuation | Level of evidence score: 0  AS 0-1.5 + PHENO: CTC-AE 4 | Warning: Individuals with a defect in the enzyme dihydropyrimidine dehydrogenase (DPD) may be susceptible to severe systemic toxicity on use of standard doses of Efudix due to an increased systemic 5-FU concentration. Evaluation of DPD activity may be considered in patients with confirmed or suspected systemic toxicity. Due to the relationship between DPD deficiency and systemic toxicity, individuals known to have DPD enzyme deficiency should be intensively monitored for systemic toxicity during Efudix treatment.  Adverse events: Frequency not known: haematological conditions, such as pancytopenia, neutropenia, thrombocytopenia, leukocytosis; haemorrhagic diarrhoea, diarrhoea, vomiting, stomach pain, stomatitis, rash, nasal mucositis.*  * Haematological conditions, stomatitis, rash, nasal mucositis (associated with systemic toxicity to medicinal products). |  |
| ref. 45 - CAP  SPC Xeloda (capecitabine) 20-04-18.  ref. 45, continuation  ref. 45, continuation | Level of evidence score: 0  AS 0: CTC-AE 5  AS 1-1.5 + PHENO: CTC-AE 4 | Contraindications: Patients with known complete absence of dihydropyrimidine dehydrogenase (DPD) activity.  Warning: Rarely, unexpected, severe toxicity (e.g. stomatitis, diarrhoea, mucosal inflammation, neutropenia and neurotoxicity) associated with 5-FU has been attributed to a deficiency of DPD activity. Patients with low or absent DPD activity, an enzyme involved in 5-FU degradation, are at increased risk for severe, life-threatening, or fatal adverse reactions caused by 5-FU. Although DPD deficiency cannot be precisely defined, it is known that patients with certain homozygous or certain compound heterozygous mutations in the *DPYD* gene locus (e.g. DPYD*2A, c.1679T>G, c.2846A>T and c.1236G>A/HapB3 variants), which can cause complete or near complete absence of DPD enzymatic activity (as determined from laboratory assays), have the highest risk of life-threatening or fatal toxicity and should not be treated with Xeloda. No dose has been proven safe for patients with complete absence of DPD activity. Patients with certain heterozygous DPYD variants (including DPYD*2A, c.1679T>G, c.2846A>T and c.1236G>A/HapB3 variants) have been shown to have increased risk of severe toxicity when treated with capecitabine.  The frequency of the heterozygous DPYD*2A genotype in the DPYD gene in Caucasian patients is around 1%, 1.1% for c.2846A>T, 2.6-6.3% for c.1236G>A/HapB3 variants and 0.07 to 0.1% for c.1679T>G. Genotyping for these alleles is recommended to identify patients at increased risk for severe toxicity. Data on the frequency of these DPYD variants in other populations than Caucasian is limited. It cannot be excluded that other rare variants may also be associated with an increased risk of severe toxicity.  For patients with partial DPD deficiency (such as those with heterozygous mutations in the *DPYD* gene) and where the benefits of Xeloda are considered to outweigh the risks (taking into account the suitability of an alternative non-fluoropyrimidine chemotherapeutic regimen), these patients must be treated with extreme caution and frequent monitoring with dose adjustment according to toxicity. There is insufficient data to recommend a specific dose in patients with partial DPD activity as measured by specific test. It has been reported that the DPYD*2A, c.1679T>G variants lead to a greater reduction in enzymatic activity than the other variants with a higher risk of side effects. The consequences of a reduced dose for efficacy are currently uncertain. Therefore, in the absence of serious toxicity the dose could be increased while carefully monitoring the patient.  The patients who are tested negative for the above-mentioned alleles may still have a risk of severe adverse events. In patients with unrecognised DPD deficiency treated with capecitabine, as well as in those patients who test negative for specific DPYD variations life-threatening toxicities manifesting as acute overdose may occur. In the event of grade 2-4 acute toxicity, treatment must be discontinued immediately. Permanent discontinuation should be considered based on clinical assessment of the onset, duration and severity of the observed toxicities. |  |
| ref. 46 – FU  SPC Fluorouracil 29-07-16 (USA) and other^1^  ref. 46, continuation | Level of evidence score: 0  AS 0: CTC-AE 5  AS 1-1.5 + PHENO: CTC-AE 5 | Warning:  Based on post-marketing reports, patients with certain homozygous or certain compound heterozygous mutations in the DPD gene that result in complete or near complete absence of DPD activity are at increased risk for acute early-onset of toxicity and severe, life-threatening, or fatal adverse reactions caused by 5-FU (e.g., mucositis, diarrhoea, neutropenia, and neurotoxicity). Patients with partial DPD activity may also have increased risk of severe, life-threatening, or fatal adverse reactions caused by 5-FU.  Withhold or permanently discontinue 5-FU based on clinical assessment of the onset, duration and severity of the observed toxicities in patients with evidence of acute early-onset or unusually severe toxicity, which may indicate near complete or total absence of DPD activity. No 5-FU dose has been proven safe for patients with complete absence of DPD activity. There is insufficient data to recommend a specific dose in patients with partial DPD activity as measured by any specific test. |  |
| ref. 47 – FU  SmPC Carac (5-fluorouracil) cream 16-12-03 (USA).  ref. 47, continuation | Level of evidence score: 0  AS 0: CTC-AE 4 | Contraindications: Carac should not be used in patients with dihydropyrimidine dehydrogenase (DPD) deficiency. DPD deficiency may lead to 5-FU entering the anabolic route, resulting in cytotoxic activity and possible toxicity.  Warning: Patients should discontinue treatment with Carac if symptoms of DPD deficiency develop.  Rare, unexpected systemic toxicity (e.g. stomatitis, diarrhoea, neutropenia and neurotoxicity) associated with parenteral administration of 5-FU has been attributed to DPD deficiency. A case of life-threatening systemic toxicity has been reported following topical use of 5% 5-FU by a patient with fully non-functional DPD. Symptoms included severe abdominal pain, haemorrhagic diarrhoea, vomiting, fever and chills. Physical examination showed stomatitis, erythematous rash, neutropenia, thrombocytopenia, inflammation of the oesophagus, stomach and small intestine. Although this patient had used 5% 5-FU cream, it is not known whether patients with severe DPD deficiency develop systemic toxicity in response to lower concentrations of topically administered 5-FU. |  |

AS= Gene Activity Score, AS 0 = gene activity score 0 = two non-functional alleles (*2A/*2A, *2A/*13 or *13/*13) or more general two gene variants leading to non-functional alleles (*2A-homozygosity, *13-homozygosity, or both *2A and *13), AS 1 = gene activity score 1 = one fully functional and one non-functional allele (*1/*2A or *1/*13), AS 1.5 = gene activity score 1.5 = one fully functional and one partially functional allele (*1/c.1236G>A or *1/c.2846A>T), AS 2 = gene activity score 2 = two fully functional alleles (extensive normal metaboliser; *1/*1), PHENO = phenotyping = two partially functional alleles (c.1236G>A/c.1236G>A, c.1236G>A/c.2846A>T or c.2846A>T/c.2846A>T) or one non-functional and one partially functional allele (*2A/c.1236G>A, *2A/c.2846A>T, *13/c.1236G>A or *13/c.2846A>T) or more general two gene variants leading to partially functional alleles (c.1236G>A-homozygosity, c.2486A>T-homozygosity, or both c.1236G>A and c.2846A>T) or a gene variant leading to a non-functional allele and a gene variant leading to a partially functional allele (*2A plus c.1236G>A, *2A plus c.2846A>T, *13 plus c.1236G>A or *13 plus c.2846A>T), 5-FU = 5-fluorouracil, 95% CI = 95% confidence interval, CAP = capecitabine, Cl = clearance, comb = combination therapy (≥ 2 oncolytic drugs, C_ss_ = steady-state plasma concentration, DPD = dihydropyrimidine dehydrogenase, mono = monotherapy (1 oncolytic drug), NS = non-significant, RR = relative risk, S = significant, SNP = single nucleotide polymorphism

^#^ For studies that did not show significant differences for IM or PM due to very low numbers of IM or PM in the study (< 4), the effect for IM or PM was scored as if this concerned a case. This was indicated by placing the case code (2) behind the score.

^1^: SmPC Xeloda (capecitabine) 14-12-16 (USA).
