# Supplementary material for: Dutch Pharmacogenetics Working Group (DPWG) guideline for the gene–drug interaction of DPYD and fluoropyrimidines
Source: Eur J Hum Genet. 2019 Nov 19;28(4):508–17. doi: 10.1038/s41431-019-0540-0 (PMC7080718; doi:10.1038/s41431-019-0540-0)
Supplement: Supplementary file 2 — Literature review of DPYD/[tegafur with DPD inhibitor] interactions to support the therapeutic dose guidelines to optimize dose [file 41431_2019_540_MOESM2_ESM.docx]

**Supplementary Table 2:** Literature review of *DPYD*/[tegafur with DPD inhibitor] interactions to support the therapeutic dose guidelines to optimize dose

| **Reference** | **Code** | **Effect** | **Comments** |
| --- | --- | --- | --- |
| ref. 1  Cubero DI et al. Tegafur-uracil is a safe alternative for the treatment of colorectal cancer in patients with partial dihydropyrimidine dehydrogenase deficiency: a proof of principle.  Ther Adv Med Oncol 2012;4:167-72.  PubMed PMID: 22754590.  ref. 1, continuation | Level of evidence score: 2  AS 1: AA | Four patients with colorectal cancer developed grade 3-4 toxicity after the first cycle of chemotherapy with 5-FU (intravenous bolus of 425 mg/m^2^ on days 1 and 5, in combination with folinic acid). They were found to be *1/*2A. After recovery, treatment with tegafur-uracil in combination with folinic acid was initiated. A full dose (100%) was tegafur 100 mg/m^2^ three times daily for 21 days followed by a week-long rest period. Doses were rounded down to multiples of 100 mg tegafur. Doses were guided by adverse events.  The first patient received 60% in the first cycle, 80% in the second cycle, 100% in the third cycle and 90% in the fourth and fifth cycles of the full dose of tegafur without development of grade 3-4 toxicity. This patient had developed grade 4 mucositis, diarrhoea and myelotoxicity on 5-FU.  The following 3 patients received 90% of the full dose of tegafur during 5 cycles without development of grade 3-4 toxicity in any of the cycles. Of the three patients, one developed grade 4 diarrhoea and grade 3 mucositis on 5-FU, the second grade 3 diarrhoea and myelotoxicity, and the third grade 3 mucositis, diarrhoea and myelotoxicity.  The best response in the first and the last patient, who both had metastatic disease, was achieving stable disease. The second and third patients receiving adjuvant chemotherapy were disease-free two years after the therapy. | Authors’ conclusion:  “Here, we demonstrate a complete absence of severe toxicity in all patients and cycles analysed. We believe that UFT is a safe alternative for the treatment of patients with partial DPD deficiency.” |
| ref. 2  Deenen MJ et al. Standard-dose tegafur combined with uracil is not safe treatment after severe toxicity from 5-fluoro-uracil or capecitabine.  Ann Intern Med 2010;153:767-8. PubMed PMID: 21135311.  ref. 2, continuation | Level of evidence score: 2  AS 1: CTC-AE 4  AS 1.5: CTC-AE 4 | - One patient developed severe abdominal cramps, grade 4 diarrhoea, grade 4 neutropenia, dehydration and severe mucositis 10 days after initiation of capecitabine 1000 mg/m^2^ BSA twice daily (in combination with oxaliplatin and bevacizumab). She recovered after discontinuation of capecitabine and 25 days at the hospital. A few months later she received tegafur-uracil 300 mg/m^2^ per day in combination with folinic acid. After 10 days, she developed severe diarrhoea, mucositis, fever, dehydration and grade 4 neutropenia. She recovered after 25 days at the hospital. The patient was *1/*2A.  - Three other patients requiring hospitalisation due to severe toxicity on 5-FU or capecitabine therapy also developed severe toxicity following treatment with standard-dose tegafur-uracil. The patients were *1/*2A, *1/c.2846A>T and *1/c.1236G>A respectively. The DPD activity was approximately 50% in the latter two patients. This confirms that they were heterozygous and did not have a second unknown non-functional allele.  The authors stated that tegafur-uracil is probably not safe in patients with partial DPD deficiency due to the greater effect of the DPD inhibitor uracil in these patients. They referred to an article that showed that uracil increases the half-life of fluorouracil to a greater extent in DPD-deficient patients, which leads to an increased risk of toxicity.  The authors also stated that the tegafur dose in tegafur-gimeracil-oteracil is 3x as low as in tegafur-uracil, while the DPD inhibitor is 200x more potent. However, 5-FU is still metabolised by DPD after administration of tegafur-gimeracil-oteracil. This means that DPD also remains essential for detoxification of 5-FU in this instance. | Authors’ conclusion:  “The standard dose of UFT is not safe after severe toxicity to 5-FU or capecitabine in DPD-deficient patients.” |
| ref. 3  SPC Teysuno (tegafur/gimeracil/oteracil) 11-04-18.  ref. 3, continuation | Level of evidence score: 0  AS 0: CTC-AE 4  AS 1-1.5 + PHENO: CTC-AE 4 | Contraindications:  Known dihydropyrimidine dehydrogenase (DPD) deficiency.  History of severe and unexpected reactions to fluoropyrimidine therapy.  Pharmacodynamics: Mean 5-FU maximum plasma concentration (C_max_) and area under the concentration-time curve (AUC) values were approximately 3-fold higher after Teysuno administration than after administration of tegafur alone, despite a 16-fold lower Teysuno dose (50 mg of tegafur) compared to tegafur alone (800 mg), and are attributed to inhibition of DPD by gimeracil. Maximum plasma uracil concentration was observed at 4 hours, with a return to baseline levels within approximately 48 hours after dosing, indicating the reversibility of DPD inhibition by gimeracil.  In man, the apparent terminal elimination half-life (T1/2) of 5-FU observed after administration of Teysuno (containing tegafur, a 5-FU prodrug) was longer (approximately 1.6-1.9 hours) than that previously reported after intravenous administration of 5-FU (10 to 20 minutes). Following a single dose of Teysuno, T1/2 values ranged from 6.7 to 11.3 hours for tegafur, from 3.1 to 4.1 hours for gimeracil and from 1.8 to 9.5 hours for oteracil.  Interactions: Sorivudine or its chemically related analogues such as brivudine irreversibly inhibit DPD, resulting in a significant increase in 5-FU exposure. This may lead to increased clinically significant fluoropyrimidine-related toxicities with potentially fatal outcomes. |  |

AS = gene activity score, AS 0 = gene activity score 0 = two non-functional alleles (*2A/*2A, *2A/*13 or *13/*13) or more general two gene variants leading to non-functional alleles (*2A-homozygosity, *13-homozygosity, or both *2A and *13), AS 1 = gene activity score 1 = one fully functional and one non-functional allele (*1/*2A or *1/*13), AS 1.5 = gene activity score 1.5 = one fully functional and one partially functional allele (*1/1236G>A or *1/c.2846A>T), AS 2 = gene activity score 2 = two fully functional alleles (extensive normal metabolizer; *1/*1), DPD = dihydropyrimidine dehydrogenase, PHENO = phenotyping = two partially functional alleles (1236G>A/1236G>A, 1236G>A/c.2846A>T or c.2846A>T/c.2846A>T), or one non-functional and one partially functional allele (*2A/1236G>A, *2A/c.2846A>T *13/1236G>A or *13/c.2846A>T) or more general two gene variants leading to partially functional alleles (1236G>A-homozygosity, 2486T-homozygosity, or both 1236G>A and c.2846A>T) or a gene variant leading to a non-functional allele and a gene variant leading to a partially functional allele (*2A plus 1236G>A, *2A plus c.2846A>T, *13 plus 1236G>A or *13 plus c.2846A>T), 5-FU = 5-fluorouracil, DPD = dihydropyrimidine dehydrogenase, NS = non-significant, S = significant, SmPC = Summary of Product Characteristics.
