# Supplementary material for: Dutch Pharmacogenetics Working Group (DPWG) guideline for the gene–drug interaction of DPYD and fluoropyrimidines
Source: Eur J Hum Genet. 2019 Nov 19;28(4):508–17. doi: 10.1038/s41431-019-0540-0 (PMC7080718; doi:10.1038/s41431-019-0540-0)
Supplement: Supplementary file 3 — Relationship between genotype result and predicted phenotype in patients carrying no variants or one or more variants leading to decreased DPD enzyme activity [file 41431_2019_540_MOESM3_ESM.docx]

**Supplementary Table 3:** Relationship between genotype result and predicted phenotype in patients carrying no variants or one or more variants leading to decreased DPD enzyme activity

| **Patients carrying no or one variant(s)** | | |
| --- | --- | --- |
| **Genotype result** | **Genotype (given as functionality of both alleles)** | **Predicted Phenotype (expected DPD enzyme activity)** |
| No aberrant variant (*1/*1) | Full functionality/full functionality | Gene activity score 2 (100% of normal DPD enzyme activity) |
| Heterozygous for variant with reduced functionality (*1/c.2846A>T or *1/c.1236G>A) | Full functionality/reduced functionality | Gene activity score 1.5 (75% of normal DPD enzyme activity) |
| Heterozygous for variant with inactive functionality (*1/*2A or *1/*13) | Full functionality/inactive functionality | Gene activity score 1 (50% of normal DPD enzyme activity) |
| Homozygous for variant with reduced functionality (c.2846A>T/c.2846A>T or c.1236G>A/c.1236G>A) | Reduced functionality/reduced functionality | PHENO:  DPD enzyme activity cannot be predicted correctly, an additional phenotyping test is required to determine the DPD enzyme activity |
| Homozygous for variant with inactive functionality (*2A/*2A or*13/*13) | Inactive functionality/inactive functionality | Gene activity score 0 (0% of normal DPD enzyme activity) |
| **Patients carrying two variants** | | |
| **Genotype result** | **Possible predicted phenotype** | **Reasoning** |
| Heterozygous for two different variants with reduced functionality  (c.2846A>T/c.1236G>A or *1/c.2846A>T+c.1236G>A) | PHENO:  DPD enzyme activity cannot be predicted correctly, an additional phenotyping test is required to determine the DPD enzyme activity | When two reduced functionality variants are located on different alleles the predicted gene activity score is 1.  When two reduced functionality variants are located on the same allele the predicted gene activity score is dependent on the effect that the two variants have on each other. This effect is unknown. If one of the two variants has no additional effect on the functionality, then the activity of the allele is equal to that without the second variant, thus 0.5, and the gene activity score is 1.5.  When the two variants act synergistic and the allele becomes fully inactive, then the activity of the allele is 0 and the gene activity score is 1.  Since the c.2846A>T and c.1236G>A variants result in reduced DPD enzyme activity through different biological mechanisms (Asp949Val amino acid substitution and an mRNA splicing-defect, respectively), it is probable that they are independent of each other regarding their effect on the allele’s functionality. This would result in an allele activity of 0.25 (each variant resulting in half of the allele functionality) and thus a gene activity score of 1.25. There is no recommendation available for gene activity score 1.25.  However, other factors than genetic variants can also affect the DPD enzyme activity. For this reason, one should resort to the recommendation for the gene activity score of 1 when the measured DPD enzyme activity is approximately equal to 50% of normal DPD enzyme activity and to the recommendation for the gene activity score of 1.5 when the measured DPD enzyme activity is approximately equal to 75% of normal DPD enzyme activity.  When the measured DPD enzyme activity is between 50% and 75% (e.g. 63%) one should resort to the recommendation for gene activity score 1. In this case, one should record a gene activity score of 1.25 in the patients’ medical record. |
| Heterozygous for variants with reduced functionality or inactive functionality (*2A/c.2846A>T or *1/*2A+c.2846A>T; *13/c.2846A>T or *1/*13+c.2846A>T; *2A/c.1236G>A or *1/*2A+c.1236G>A; *13/c.1236G>A or *1/*13+c.1236G>A) | PHENO:  DPD enzyme activity cannot be predicted correctly, an additional phenotyping test is required to determine the DPD enzyme activity | When two variants are located on different alleles the gene activity score is 0.5 (one allele with reduced functionality and one allele with inactive functionality).  When two variants are located on the same allele, the gene activity score is 1 (one allele with full functionality and one allele with inactive functionality). |
| Heterozygous for two different variants with inactive functionality  (*2A/*13 or *1/*2A+*13) | PHENO:  DPD enzyme activity cannot be predicted correctly, an additional phenotyping test is required to determine the DPD enzyme activity | When two variants are located on different alleles the gene activity score is 0 (two alleles with inactive functionality).  When two variants are located on the same allele the gene activity score is 1 (one allele with full functionality and one allele with inactive functionality). |
| Homozygous for one variant with reduced functionality and heterozygous for the other variant with reduced functionality  (c.2846A>T/c.2846A>T+c.1236G>A or c.1236G>A/c.2846A>T+ c.1236G>A) | PHENO:  DPD enzyme activity cannot be predicted correctly, an additional phenotyping test is required to determine the DPD enzyme activity | One of the alleles has an activity of 0.5. The activity of the other allele is unknown, but lies between 0 and 0.5 (see reasoning for heterozygous for two different alleles with reduced functionality).  One should resort to the recommendation for gene activity score 1 when the DPD enzyme activity is 50% of normal DPD enzyme activity. |
| Homozygous for a variant with reduced functionality and heterozygous for a variant with inactive functionality (c.2846A>T/*2A+c.2846A>T or c.2846A>T/*13+c.2846A>T or c.1236G>A/*2A+c.1236G>A or c.1236G>A/*13+c.1236G>A) | PHENO:  DPD enzyme activity cannot be predicted correctly, an additional phenotyping test is required to determine the DPD enzyme activity | One of the alleles has an activity of 0.5, the activity of the other allele is 0. Therefore the gene activity score is 0.5 (PHENO). |
| Heterozygous for a variant with reduced functionality and homozygous for a variant with inactive functionality  (*2A/*2A+c.2846A>T or *2A/*2A+c.1236G>A or *13/*13+c.2846A>T or *13/*13+c.1236G>A) | Gene activity score 0 | Both alleles have an activity of 0. Therefore the gene activity score is 0. |
| Homozygous for a variant with inactive functionality and heterozygous for the other variant with inactive functionality (*2A/*2A+*13 or *13/*2A+*13) | Gene activity score 0 | Both alleles have an activity of 0. Therefore the gene activity score is 0. |
| Homozygous for two different variants with reduced functionality  (c.2846A>T+c.1236G>A/ c.2846A>T+c.1236G>A) | PHENO:  DPD enzyme activity cannot be predicted correctly, an additional phenotyping test is required to determine the DPD enzyme activity | The activity of both alleles is unknown, but lies between 0 and 0.5 (see reasoning for heterozygous for two different reduced functionality alleles).  One should resort to the recommendation for gene activity score 0 when the measured DPD enzyme activity is approximately 0% of normal DPD enzyme activity and the recommendation of gene activity score 1 when the DPD enzyme activity is 50% of normal DPD enzyme activity.. |
| Homozygous for a variant with reduced functionality and a variant with inactive functionality (*2A+c.2846A>T/*2A+c.2846A>T or *13+c.2846A>T/*13+ c.2846A>T or *2A+c.1236G>A/*2A+c.1236G>A or *13+c.1236G>A/*13+ c.1236G>A) | Gene activity score 0 | Both alleles have an activity of 0. Therefore the gene activity score is 0. |
| Homozygous for two different variants with inactive functionality  (*2A+*13/*2A+ *13) | Gene activity score 0 | Both alleles have an activity of 0. Therefore the gene activity score is 0. |
| **Patients carrying three or more variants** | | |
| **Genotype result** | **Reasoning for finding the possible predicted phenotype** | |
| Three or more variants | Since patients carrying three or more different variants are rare, only a general explanation of how to predict the phenotype is given. If one does encounter a patient carrying three or more variants, one must determine how these variants can be located among two alleles and determine if this leads to different predicted phenotypes.  Since there are only two validated variants which result in a reduced functionality, an allele with three different variants will always have a variant with an inactive functionality and therefore the allele will have an activity of 0. The predicted allele activities for alleles with 0, 1 or 2 variants are indicated in the tables above.  If all possible distributions of the variants across the alleles lead to the same gene activity score of the genotype (i.e. the sum of allele activities), then one can conclude this as the patient’s gene activity score.  If different distributions lead to genotypes with different gene activity scores, phenotyping is required to quantify DPD enzyme activity. | |

DPD: dihydropyrimidine dehydrogenase
