# Supplementary material for: Dutch Pharmacogenetics Working Group (DPWG) guideline for the gene–drug interaction of DPYD and fluoropyrimidines
Source: Eur J Hum Genet. 2019 Nov 19;28(4):508–17. doi: 10.1038/s41431-019-0540-0 (PMC7080718; doi:10.1038/s41431-019-0540-0)
Supplement: Supplementary file 4 — Genotype to predicted phenotype translation to be programmed into laboratory information system [file 41431_2019_540_MOESM4_ESM.docx]

**Supplementary Table 4:** Genotype to predicted phenotype translation to be programmed into laboratory information system

| **Genotype** | **rs number variants** | **Nucleotide at position** | **Dose recommendation according to gene activity score** |
| --- | --- | --- | --- |
| *DPYD*: WILDTYPE/WILDTYPE | *DPYD*_rs3918290  *DPYD*_rs55886062  *DPYD*_rs56038477  *DPYD*_rs67376798 | G:G  T:T  G:G  A:A | GENE ACTIVITY SCORE 2 |
| *DPYD*: WILDTYPE/*2A | *DPYD*_rs3918290  *DPYD*_rs55886062  *DPYD*_rs56038477  *DPYD*_rs67376798 | G:A *2A  T:T  G:G  A:A | GENE ACTIVITY SCORE 1 |
| *DPYD*: WILDTYPE/*13 | *DPYD*_rs3918290  *DPYD*_rs55886062  *DPYD*_rs56038477  *DPYD*_rs67376798 | G:G  T:G *13  G:G  A:A | GENE ACTIVITY SCORE 1 |
| *DPYD*: WILDTYPE/c.2846A>T | *DPYD*_rs3918290  *DPYD*_rs55886062  *DPYD*_rs56038477  *DPYD*_rs67376798 | G:G  T:T  G:G  A:T c.2846A>T | GENE ACTIVITY SCORE 1,5 |
| *DPYD*: WILDTYPE/c.1236G>A | *DPYD*_rs3918290  *DPYD*_rs55886062  *DPYD*_rs56038477  *DPYD*_rs67376798 | G:G  T:T  G:A c.1236G>A  A:A | GENE ACTIVITY SCORE 1,5 |
| *DPYD*: *2A/*2A | *DPYD*_rs3918290  *DPYD*_rs55886062  *DPYD*_rs56038477  *DPYD*_rs67376798 | A:A *2A  T:T  G:G  A:A | GENE ACTIVITY SCORE 0 |
| *DPYD*: *13/*13 | *DPYD*_rs3918290  *DPYD*_rs55886062  *DPYD*_rs56038477  *DPYD*_rs67376798 | G:G  G:G *13  G:G  A:A | GENE ACTIVITY SCORE 0 |
| *DPYD*: c.2846A>T/c.2846A>T | *DPYD*_rs3918290  *DPYD*_rs55886062  *DPYD*_rs56038477  *DPYD*_rs67376798 | G:G  T:T  G:G  T:T c.2846A>T | PHENO:  DPD enzyme activity cannot be predicted correctly, an additional phenotyping test is required to determine the DPD enzyme activity |
| *DPYD*: c.1236G>A/c.1236G>A | *DPYD*_rs3918290  *DPYD*_rs55886062  *DPYD*_rs56038477  *DPYD*_rs67376798 | G:G  T:T  A:A c.1236G>A  A:A | PHENO:  DPD enzyme activity cannot be predicted correctly, an additional phenotyping test is required to determine the DPD enzyme activity |
| *DPYD*: WILDTYPE/*2A  WILDTYPE/*13 | *DPYD*_rs3918290  *DPYD*_rs55886062  *DPYD*_rs56038477  *DPYD*_rs67376798 | G:A *2A  T:G *13  G:G  A:A | GENE ACTIVITY SCORE 0 |
| *DPYD*: WILDTYPE/*2A  WILDTYPE/c.1236G>A | *DPYD*_rs3918290  *DPYD*_rs55886062  *DPYD*_rs56038477  *DPYD*_rs67376798 | G:A *2A  T:T  G:A c.1236G>A  A:A | Unable to predict the gene activity score. Phenotyping should distinguish if both variants are present on separate alleles (GENE ACTIVITY SCORE 0.5(PHENO)) or on the same allele (GENE ACTIVITY SCORE 1). |
| *DPYD*: WILDTYPE/*2A  WILDTYPE/c.2846A>T | *DPYD*_rs3918290  *DPYD*_rs55886062  *DPYD*_rs56038477  *DPYD*_rs67376798 | G:A *2A  T:T  G:G  A:T c.2846A>T | Unable to predict the gene activity score. Phenotyping should distinguish if both variants are present on separate alleles (GENE ACTIVITY SCORE 0.5(PHENO)) or on the same allele (GENE ACTIVITY SCORE 1). |
| *DPYD*: WILDTYPE/*13  WILDTYPE/c.1236G>A | *DPYD*_rs3918290  *DPYD*_rs55886062  *DPYD*_rs56038477  *DPYD*_rs67376798 | G:G  T:G *13  G:A c.1236G>A  A:A | Unable to predict the gene activity score. Phenotyping should distinguish if both variants are present on separate alleles (GENE ACTIVITY SCORE 0.5(PHENO)) or on the same allele (GENE ACTIVITY SCORE 1). |
| *DPYD*: WILDTYPE/*13  WILDTYPE/c.2846A>T | *DPYD*_rs3918290  *DPYD*_rs55886062  *DPYD*_rs56038477  *DPYD*_rs67376798 | G:G  T:G *13  G:G  A:T c.2846A>T | Unable to predict the gene activity score. Phenotyping should distinguish if both variants are present on separate alleles (GENE ACTIVITY SCORE 0.5(PHENO)) or on the same allele (GENE ACTIVITY SCORE 1). |
| *DPYD*: WILDTYPE/c.1236G>A  WILDTYPE/c.2846A>T | *DPYD*_rs3918290  *DPYD*_rs55886062  *DPYD*_rs56038477  *DPYD*_rs67376798 | G:G  T:T  G:A c.1236G>A  A:T c.2846A>T | Unable to predict the gene activity score. Phenotyping should distinguish if both variants are present on separate alleles (GENE ACTIVITY SCORE 1) or on the same allele (GENE ACTIVITY SCORE 1 to 1.5). When both variants are located on the same allele, it is not known whether the variants have an independent or synergistic effect or whether the second variant does not have an additional effect. |
| *DPYD*: c.1236G>A/c.1236G>A  c.2846A>T/c.2846A>T | *DPYD*_rs3918290  *DPYD*_rs55886062  *DPYD*_rs56038477  *DPYD*_rs67376798 | G:G  T:T  A:A c.1236G>A  T:T c.2846A>T | Unable to predict the gene activity score (GENE ACTIVITY SCORE 0 to 1). Both variants are located on the same allele, but it is not known whether the variants have an independent or synergistic effect or whether the second variant does not have an additional effect. |
| *DPYD*: WILDTYPE/c.1236G>A  c.2846A>T/c.2846A>T | *DPYD*_rs3918290  *DPYD*_rs55886062  *DPYD*_rs56038477  *DPYD*_rs67376798 | G:G  T:T  G:A c.1236G>A  T:T c.2846A>T | Unable to predict the gene activity score (GENE ACTIVITY SCORE 0.5(PHENO) to 1). There is one allele with one variant and one allele with two variants, but it is not known whether the two variants on the same allele have an independent or synergistic effect or whether the second variant does not have an additional effect. |
| *DPYD*: c.1236G>A/c.1236G>A  WILDTYPE/c.2846A>T | *DPYD*_rs3918290  *DPYD*_rs55886062  *DPYD*_rs56038477  *DPYD*_rs67376798 | G:G  T:T  A:A c.1236G>A  A:T c.2846A>T | Unable to predict the gene activity score (GENE ACTIVITY SCORE 0.5(PHENO) to 1). There is one allele with one variant and one allele with two variants, but it is not known whether the two variants on the same allele have an independent or synergistic effect or whether the second variant does not have an additional effect. |
| *DPYD*: *13/*13  c.1236G>A/c.1236G>A | *DPYD*_rs3918290  *DPYD*_rs55886062  *DPYD*_rs56038477  *DPYD*_rs67376798 | G:G  G:G *13  A:A c.1236G>A  A:A | GENE ACTIVITY SCORE 0 |
| *DPYD*: *13/*13  WILDTYPE/c.1236G>A | *DPYD*_rs3918290  *DPYD*_rs55886062  *DPYD*_rs56038477  *DPYD*_rs67376798 | G:G  G:G *13  G:A c.1236G>A  A:A | GENE ACTIVITY SCORE 0 |
| *DPYD*: *13/*13  c.2846A>T/c.2846A>T | *DPYD*_rs3918290  *DPYD*_rs55886062  *DPYD*_rs56038477  *DPYD*_rs67376798 | G:G  G:G *13  G:G  T:T c.2846A>T | GENE ACTIVITY SCORE 0 |
| *DPYD*: *13/*13  WILDTYPE/c.2846A>T | *DPYD*_rs3918290  *DPYD*_rs55886062  *DPYD*_rs56038477  *DPYD*_rs67376798 | G:G  G:G *13  G:G  A:T c.2846A>T | GENE ACTIVITY SCORE 0 |
| *DPYD*: *13/*13  *2A/*2A | *DPYD*_rs3918290  *DPYD*_rs55886062  *DPYD*_rs56038477  *DPYD*_rs67376798 | A:A *2A  G:G *13  G:G  A:A | GENE ACTIVITY SCORE 0 |
| *DPYD*: *13/*13  WILDTYPE/*2A | *DPYD*_rs3918290  *DPYD*_rs55886062  *DPYD*_rs56038477  *DPYD*_rs67376798 | G:A *2A  G:G *13  G:G  A:A | GENE ACTIVITY SCORE 0 |
| *DPYD*: *2A/*2A  c.1236G>A/c.1236G>A | *DPYD*_rs3918290  *DPYD*_rs55886062  *DPYD*_rs56038477  *DPYD*_rs67376798 | A:A *2A  T:T  A:A c.1236G>A  A:A | GENE ACTIVITY SCORE 0 |
| *DPYD*: *2A/*2A  WILDTYPE/c.1236G>A | *DPYD*_rs3918290  *DPYD*_rs55886062  *DPYD*_rs56038477  *DPYD*_rs67376798 | A:A *2A  T:T  G:A c.1236G>A  A:A | GENE ACTIVITY SCORE 0 |
| *DPYD*: *2A/*2A  c.2846A>T/c.2846A>T | *DPYD*_rs3918290  *DPYD*_rs55886062  *DPYD*_rs56038477  *DPYD*_rs67376798 | A:A *2A  T:T  G:G  T:T c.2846A>T | GENE ACTIVITY SCORE 0 |
| *DPYD*: *2A/*2A  WILDTYPE/c.2846A>T | *DPYD*_rs3918290  *DPYD*_rs55886062  *DPYD*_rs56038477  *DPYD*_rs67376798 | A:A *2A  T:T  G:G  A:T c.2846A>T | GENE ACTIVITY SCORE 0 |
| *DPYD*: *2A/*2A  WILDTYPE/*13 | *DPYD*_rs3918290  *DPYD*_rs55886062  *DPYD*_rs56038477  *DPYD*_rs67376798 | A:A *2A  T:G *13  G:G  A:A | GENE ACTIVITY SCORE 0 |
| *DPYD*: c.1236G>A/c.1236G>A  WILDTYPE/*2A | *DPYD*_rs3918290  *DPYD*_rs55886062  *DPYD*_rs56038477  *DPYD*_rs67376798 | G:A *2A  T:T  A:A c.1236G>A  A:A | PHENO:  DPD enzyme activity cannot be predicted correctly, an additional phenotyping test is required to determine the DPD enzyme activity |
| *DPYD*: c.1236G>A/c.1236G>A  WILDTYPE/*13 | *DPYD*_rs3918290  *DPYD*_rs55886062  *DPYD*_rs56038477  *DPYD*_rs67376798 | G:G  T:G *13  A:A c.1236G>A  A:A | PHENO:  DPD enzyme activity cannot be predicted correctly, an additional phenotyping test is required to determine the DPD enzyme activity |
| *DPYD*: c.2846A>T/c.2846A>T  WILDTYPE/*2A | *DPYD*_rs3918290  *DPYD*_rs55886062  *DPYD*_rs56038477  *DPYD*_rs67376798 | G:A *2A  T:T  G:G  T:T c.2846A>T | PHENO:  DPD enzyme activity cannot be predicted correctly, an additional phenotyping test is required to determine the DPD enzyme activity |
| *DPYD*: c.2846A>T/c.2846A>T  WILDTYPE/*13 | *DPYD*_rs3918290  *DPYD*_rs55886062  *DPYD*_rs56038477  *DPYD*_rs67376798 | G:G  T:G *13  G:G  T:T c.2846A>T | PHENO:  DPD enzyme activity cannot be predicted correctly, an additional phenotyping test is required to determine the DPD enzyme activity |
| *DPYD*: *13/*13  c.1236G>A/c.1236G>A  c.2846A>T/c.2846A>T | *DPYD*_rs3918290  *DPYD*_rs55886062  *DPYD*_rs56038477  *DPYD*_rs67376798 | G:G  G:G *13  A:A c.1236G>A  T:T c.2846A>T | GENE ACTIVITY SCORE 0 |
| *DPYD*: *13/*13  wildtype/c.1236G>A  c.2846A>T/c.2846A>T | *DPYD*_rs3918290  *DPYD*_rs55886062  *DPYD*_rs56038477  *DPYD*_rs67376798 | G:G  G:G *13  G:A c.1236G>A  T:T c.2846A>T | GENE ACTIVITY SCORE 0 |
| *DPYD*: *13/*13  c.1236G>A/c.1236G>A  wildtype/c.2846A>T | *DPYD*_rs3918290  *DPYD*_rs55886062  *DPYD*_rs56038477  *DPYD*_rs67376798 | G:G  G:G *13  A:A c.1236G>A  A:T c.2846A>T | GENE ACTIVITY SCORE 0 |
| *DPYD*: *13/*13  wildtype/c.1236G>A  wildtype/c.2846A>T | *DPYD*_rs3918290  *DPYD*_rs55886062  *DPYD*_rs56038477  *DPYD*_rs67376798 | G:G  G:G *13  G:A c.1236G>A  A:T c.2846A>T | GENE ACTIVITY SCORE 0 |
| *DPYD*: wildtype/*13  c.1236G>A/c.1236G>A  c.2846A>T/c.2846A>T | *DPYD*_rs3918290  *DPYD*_rs55886062  *DPYD*_rs56038477  *DPYD*_rs67376798 | G:G  T:G *13  A:A c.1236G>A  T:T c.2846A>T | Unable to predict the gene activity score (GENE ACTIVITY SCORE 0 to 0.5(PHENO)). The activity of the allele with two variants (c.1236G>A and c.2846A>T) is not known, because it is not known whether the variants have an independent or synergistic effect or whether the second variant does not have an additional effect. |
| *DPYD*: wildtype/*13  wildtype/c.1236G>A  c.2846A>T/c.2846A>T | *DPYD*_rs3918290  *DPYD*_rs55886062  *DPYD*_rs56038477  *DPYD*_rs67376798 | G:G  T:G *13  G:A c.1236G>A  T:T c.2846A>T | Unable to predict the gene activity score. Phenotyping should distinguish if *13 and c.1236G>A are present on separate alleles (GENE ACTIVITY SCORE 0 to 0.5(PHENO)) or on the same allele (GENE ACTIVITY SCORE 0.5(PHENO)). When both variants are located on separate alleles, the activity of the allele with the two variants c.1236G>A and c.2846A>T is not known, because it is not known whether the variants have an independent or synergistic effect or whether the second variant does not have an additional effect. |
| *DPYD*: wildtype/*13  c.1236G>A/c.1236G>A  wildtype/c.2846A>T | *DPYD*_rs3918290  *DPYD*_rs55886062  *DPYD*_rs56038477  *DPYD*_rs67376798 | G:G  T:G *13  A:A c.1236G>A  A:T c.2846A>T | Unable to predict the gene activity score. Phenotyping should distinguish if *13 and c.2846A>T are present on separate alleles (GENE ACTIVITY SCORE 0 to 0.5(PHENO)) or on the same allele (GENE ACTIVITY SCORE 0.5(PHENO)). When both variants are located on separate alleles, the activity of the allele with the two variants c.1236G>A and c.2846A>T is not known, because it is not known whether the variants have an independent or synergistic effect or whether the second variant does not have an additional effect. |
| *DPYD*: wildtype/*13  wildtype/c.1236G>A  wildtype/c.2846A>T | *DPYD*_rs3918290  *DPYD*_rs55886062  *DPYD*_rs56038477  *DPYD*_rs67376798 | G:G  T:G *13  G:A c.1236G>A  A:T c.2846A>T | Unable to predict the gene activity score (GENE ACTIVITY SCORE 0 to 1). Phenotyping should distinguish which variants are present on the same allele. The activity of an allele with the two variants c.1236G>A and c.2846A>T is not known, because it is not known whether the variants have an independent or synergistic effect or whether the second variant does not have an additional effect. |
| *DPYD*: *2A/*2A  c.1236G>A/c.1236G>A  c.2846A>T/c.2846A>T | *DPYD*_rs3918290  *DPYD*_rs55886062  *DPYD*_rs56038477  *DPYD*_rs67376798 | A:A *2A  T:T  A:A c.1236G>A  T:T c.2846A>T | GENE ACTIVITY SCORE 0 |
| *DPYD*: *2A/*2A  wildtype/c.1236G>A  c.2846A>T/c.2846A>T | *DPYD*_rs3918290  *DPYD*_rs55886062  *DPYD*_rs56038477  *DPYD*_rs67376798 | A:A *2A  T:T  G:A c.1236G>A  T:T c.2846A>T | GENE ACTIVITY SCORE 0 |
| *DPYD*: *2A/*2A  c.1236G>A/c.1236G>A  wildtype/c.2846A>T | *DPYD*_rs3918290  *DPYD*_rs55886062  *DPYD*_rs56038477  *DPYD*_rs67376798 | A:A *2A  T:T  A:A c.1236G>A  A:T c.2846A>T | GENE ACTIVITY SCORE 0 |
| *DPYD*: *2A/*2A  wildtype/c.1236G>A  wildtype/c.2846A>T | *DPYD*_rs3918290  *DPYD*_rs55886062  *DPYD*_rs56038477  *DPYD*_rs67376798 | A:A *2A  T:T  G:A c.1236G>A  A:T c.2846A>T | GENE ACTIVITY SCORE 0 |
| *DPYD*: *2A/*2A  *13/*13  c.1236G>A/c.1236G>A | *DPYD*_rs3918290  *DPYD*_rs55886062  *DPYD*_rs56038477  *DPYD*_rs67376798 | A:A *2A  G:G *13  A:A c.1236G>A  A:A | GENE ACTIVITY SCORE 0 |
| *DPYD*: *2A/*2A  *13/*13  wildtype/c.1236G>A | *DPYD*_rs3918290  *DPYD*_rs55886062  *DPYD*_rs56038477  *DPYD*_rs67376798 | A:A *2A  G:G *13  G:A c.1236G>A  A:A | GENE ACTIVITY SCORE 0 |
| *DPYD*: *2A/*2A  *13/*13  c.2846A>T/c.2846A>T | *DPYD*_rs3918290  *DPYD*_rs55886062  *DPYD*_rs56038477  *DPYD*_rs67376798 | A:A *2A  G:G *13  G:G  T:T c.2846A>T | GENE ACTIVITY SCORE 0 |
| *DPYD*: *2A/*2A  *13/*13  wildtype/c.2846A>T | *DPYD*_rs3918290  *DPYD*_rs55886062  *DPYD*_rs56038477  *DPYD*_rs67376798 | A:A *2A  G:G *13  G:G  A:T c.2846A>T | GENE ACTIVITY SCORE 0 |
| *DPYD*: *2A/*2A  wildtype/*13  c.1236G>A/c.1236G>A | *DPYD*_rs3918290  *DPYD*_rs55886062  *DPYD*_rs56038477  *DPYD*_rs67376798 | A:A *2A  T:G *13  A:A c.1236G>A  A:A | GENE ACTIVITY SCORE 0 |
| *DPYD*: *2A/*2A  wildtype/*13  wildtype/c.1236G>A | *DPYD*_rs3918290  *DPYD*_rs55886062  *DPYD*_rs56038477  *DPYD*_rs67376798 | A:A *2A  T:G *13  G:A c.1236G>A  A:A | GENE ACTIVITY SCORE 0 |
| *DPYD*: *2A/*2A  wildtype/*13  c.2846A>T/c.2846A>T | *DPYD*_rs3918290  *DPYD*_rs55886062  *DPYD*_rs56038477  *DPYD*_rs67376798 | A:A *2A  T:G *13  G:G  T:T c.2846A>T | GENE ACTIVITY SCORE 0 |
| *DPYD*: *2A/*2A  wildtype/*13  wildtype/c.2846A>T | *DPYD*_rs3918290  *DPYD*_rs55886062  *DPYD*_rs56038477  *DPYD*_rs67376798 | A:A *2A  T:G *13  G:G  A:T c.2846A>T | GENE ACTIVITY SCORE 0 |
| *DPYD*: wildtype/*2A  c.1236G>A/c.1236G>A  c.2846A>T/c.2846A>T | *DPYD*_rs3918290  *DPYD*_rs55886062  *DPYD*_rs56038477  *DPYD*_rs67376798 | G:A *2A  T:T  A:A c.1236G>A  T:T c.2846A>T | Unable to predict the gene activity score (GENE ACTIVITY SCORE 0 to 0.5(PHENO)). The activity of the allele with two variants (c.1236G>A and c.2846A>T) is not known, because it is not known whether the variants have an independent or synergistic effect or whether the second variant does not have an additional effect. |
| *DPYD*: wildtype/*2A  wildtype/c.1236G>A  c.2846A>T/c.2846A>T | *DPYD*_rs3918290  *DPYD*_rs55886062  *DPYD*_rs56038477  *DPYD*_rs67376798 | G:A *2A  T:T  G:A c.1236G>A  T:T c.2846A>T | Unable to predict the gene activity score. Phenotyping should distinguish if *2A and c.1236G>A are present on separate alleles (GENE ACTIVITY SCORE 0 to 0.5(PHENO)) or on the same allele (GENE ACTIVITY SCORE 0.5(PHENO)). When both variants are located on separate alleles, the activity of the allele with the two variants c.1236G>A and c.2846A>T is not known, because it is not known whether the variants have an independent or synergistic effect or whether the second variant does not have an additional effect. |
| *DPYD*: wildtype/*2A  c.1236G>A/c.1236G>A  wildtype/c.2846A>T | *DPYD*_rs3918290  *DPYD*_rs55886062  *DPYD*_rs56038477  *DPYD*_rs67376798 | G:A *2A  T:T  A:A c.1236G>A  A:T c.2846A>T | Unable to predict the gene activity score. Phenotyping should distinguish if *2A and c.2846A>T are present on separate alleles (GENE ACTIVITY SCORE 0 to 0.5(PHENO)) or on the same allele (GENE ACTIVITY SCORE 0.5(PHENO)). When both variants are located on separate alleles, the activity of the allele with the two variants c.1236G>A and c.2846A>T is not known, because it is not known whether the variants have an independent or synergistic effect or whether the second variant does not have an additional effect. |
| *DPYD*: wildtype/*2A  wildtype/c.1236G>A  wildtype/c.2846A>T | *DPYD*_rs3918290  *DPYD*_rs55886062  *DPYD*_rs56038477  *DPYD*_rs67376798 | G:A *2A  T:T  G:A c.1236G>A  A:T c.2846A>T | Unable to predict the gene activity score (GENE ACTIVITY SCORE 0 to 1). Phenotyping should distinguish which variants are present on the same allele. The activity of an allele with the two variants c.1236G>A and c.2846A>T is not known, because it is not known whether the variants have an independent or synergistic effect or whether the second variant does not have an additional effect. |
| *DPYD*: wildtype/*2A  *13/*13  c.1236G>A/c.1236G>A | *DPYD*_rs3918290  *DPYD*_rs55886062  *DPYD*_rs56038477  *DPYD*_rs67376798 | G:A *2A  G:G *13  A:A c.1236G>A  A:A | GENE ACTIVITY SCORE 0 |
| *DPYD*: wildtype/*2A  *13/*13  wildtype/c.1236G>A | *DPYD*_rs3918290  *DPYD*_rs55886062  *DPYD*_rs56038477  *DPYD*_rs67376798 | G:A *2A  G:G *13  G:A c.1236G>A  A:A | GENE ACTIVITY SCORE 0 |
| *DPYD*: wildtype/*2A  *13/*13  c.2846A>T/c.2846A>T | *DPYD*_rs3918290  *DPYD*_rs55886062  *DPYD*_rs56038477  *DPYD*_rs67376798 | G:A *2A  G:G *13  G:G  T:T c.2846A>T | GENE ACTIVITY SCORE 0 |
| *DPYD*: wildtype/*2A  *13/*13  wildtype/c.2846A>T | *DPYD*_rs3918290  *DPYD*_rs55886062  *DPYD*_rs56038477  *DPYD*_rs67376798 | G:A *2A  G:G *13  G:G  A:T c.2846A>T | GENE ACTIVITY SCORE 0 |
| *DPYD*: wildtype/*2A  wildtype/*13  c.1236G>A/c.1236G>A | *DPYD*_rs3918290  *DPYD*_rs55886062  *DPYD*_rs56038477  *DPYD*_rs67376798 | G:A *2A  T:G *13  A:A c.1236G>A  A:A | Unable to predict the gene activity score. Phenotyping should distinguish if *2A and *13 are present on separate alleles (GENE ACTIVITY SCORE 0) or on the same allele (GENE ACTIVITY SCORE 0.5(PHENO)). |
| *DPYD*: wildtype/*2A  wildtype/*13  wildtype/c.1236G>A | *DPYD*_rs3918290  *DPYD*_rs55886062  *DPYD*_rs56038477  *DPYD*_rs67376798 | G:A *2A  T:G *13  G:A c.1236G>A  A:A | Unable to predict the gene activity score (GENE ACTIVITY SCORE 0 to 1). Phenotyping should distinguish which variants are present on the same allele. |
| *DPYD*: wildtype/*2A  wildtype/*13  c.2846A>T/c.2846A>T | *DPYD*_rs3918290  *DPYD*_rs55886062  *DPYD*_rs56038477  *DPYD*_rs67376798 | G:A *2A  T:G *13  G:G  T:T c.2846A>T | Unable to predict the gene activity score. Phenotyping should distinguish if *2A and *13 are present on separate alleles (GENE ACTIVITY SCORE 0) or on the same allele (GENE ACTIVITY SCORE 0.5(PHENO)). |
| *DPYD*: wildtype/*2A  wildtype/*13  wildtype/c.2846A>T | *DPYD*_rs3918290  *DPYD*_rs55886062  *DPYD*_rs56038477  *DPYD*_rs67376798 | G:A *2A  T:G *13  G:G  A:T c.2846A>T | Unable to predict the gene activity score (GENE ACTIVITY SCORE 0 to 1). Phenotyping should distinguish which variants are present on the same allele. |

^#^NOTE: In patients with two different gene variants, gene activity score is dependent on location of the variants on the alleles. The variants can either be located on the same allele (resulting in one affected allele with reduced or absent DPD activity and one fully functional allele) or located on different alleles (resulting in two affected alleles).
