# Supplementary material for: Dutch Pharmacogenetics Working Group (DPWG) guideline for the gene–drug interaction of DPYD and fluoropyrimidines
Source: Eur J Hum Genet. 2019 Nov 19;28(4):508–17. doi: 10.1038/s41431-019-0540-0 (PMC7080718; doi:10.1038/s41431-019-0540-0)
Supplement: Supplementary file 5 — Dutch Pharmacogenetics Working Group (DPWG) Guideline for DPYD and 5-FU/capecitabine [file 41431_2019_540_MOESM5_ESM.docx]

**Supplementary Table 5:** Dutch Pharmacogenetics Working Group (DPWG) Guideline for *DPYD* and 5-FU/capecitabine: the therapeutic recommendation and its rationale, and the kinetic and clinical consequences for each aberrant gene activity score

| **Predicted phenotype** | **Therapeutic recommendation** | **Rationale of the therapeutic recommendation** | **Kinetic consequence** | **Clinical consequence** |
| --- | --- | --- | --- | --- |
| Gene activity score 0  References:  (1-13) | SYSTEMIC ROUTE OF ADMINISTRATION:  **Avoid fluorouracil and capecitabine**  Tegafur is not an alternative, as this is also metabolised by DPD.  **If it is not possible to avoid fluorouracil and capecitabine: determine the residual DPD activity in mononuclear cells from peripheral blood and adjust the initial dose accordingly.**  A patient with 0.5% of the normal DPD activity tolerated 0.8% of the standard dose (150 mg capecitabine every 5 days). A patient with undetectable DPD activity tolerated 0.43% of the standard dose (150 mg capecitabine every 5 days with every third dose skipped).  CUTANEOUS ROUTE OF ADMINISTRATION:  **Avoid fluorouracil**  NOTE: If a patient has two different genetic variations that lead to a non-functional DPD enzyme (e.g. *2A and *13), this recommendation only applies if the variations are on a different allele. If both variations are on the same allele, this patient actually has a gene activity score 1, for which no increased risk of severe, potentially fatal toxicity has been found with cutaneous use. These two situations can only be distinguished by determining the enzyme activity (phenotyping). This recommendation only applies if the patient has virtually no enzyme activity. | There are not enough data to be able to make a substantiated recommendation on dose adjustments for gene activity 0. The recommendation for *1/*2A is a dose reduction by 50%. This would be equivalent to a dose reduction by 100% for *2A/*2A and therefore a dose reduction to 0. This agrees with the severe toxicity found in one patient with genotype *2A/*2A when using 5-fluorouracil cream on the scalp. Because of the indications that the tolerated dose is close to zero and the scarce data on tolerated doses in patients with gene activity 0 (see below), an alternative is recommended.  The calculated dose reduction based on 2 patients is a reduction to 0.81% of the normal dose (0.72-0.89%; median 0.81%). However, this is based on too few patients to be used for a substantiated dose recommendation. In addition, in one of these patients, having undetectable DPD activity, the dose had to be reduced from 0.65% to 0.43% of the normal dose during treatment. However, there is a fairly good correlation between the residual DPD enzyme activity in peripheral blood mononuclear cells and the tolerated dose (Meulendijks 2016, Deenen 2016, Henricks 2017 and Henricks 2018 Int J Cancer, Henricks 2018 Lancet Oncol and Lunenburg 2018 Genes (Basel)). Therefore, if an alternative is not possible, adjusting the dose according to the residual DPD enzyme activity in peripheral blood mononuclear cells is recommended. This strategy has been shown to be feasible in two patients with gene activity 0. A patient with 0.5% of the normal DPD activity tolerated 0.8% of the normal dose (150 mg capecitabine every 5 days) (Henricks 2018 Int J Cancer). A patient with undetectable DPD activity, tolerated 0.43% of the normal dose (150 mg capecitabine every 5 days with every third dose skipped) (Henricks 2017). | For 2 patients with gene activity score 0 (genotypes *2A/(duplication of exon 17 and 18) and *2A/*2A) the dose-corrected AUC of fluorouracil increased by a factor 113 and 138 respectively after the first systemic capecitabine dose.  Extrapolation of the decrease in clearance by 50% identified for *1/*2A would suggest a clearance of 0% for *2A/*2A (gene activity score 0). This is equivalent to severe toxicity found in one patient with *2A/*2A after using fluorouracil cream on the scalp and the two previously described patients using very low tolerated systemic doses (0.8% and 0.43% of the standard dose). | SYSTEMIC ROUTE OF ADMINISTRATION:  All patients with gene activity score 0 with known toxicity (n=2, *2A/*2A), had grade III/IV toxicity and 50% died due to toxicity. Moreover, a patient with *2A/*2A developed severe toxicity after treatment with cutaneous fluorouracil cream.  CUTANEOUS ROUTE OF ADMINISTRATION:  A patient with *2A/*2A developed severe toxicity after treatment with cutaneous fluorouracil cream. All patients using systemic fluorouracil with gene activity score 0 with known toxicity (n=2, both *2A/*2A), had grade III/IV toxicity and 50% died due to toxicity. |
| PHENO  References:  (3-5, 8-12, 14-17) | It is not possible to recommend a dose adjustment for these patients based on the genotype only.  **Determine the residual DPD activity in mononuclear cells from peripheral blood** **and adjust the initial dose based on phenotype and genotype, or avoid fluorouracil and capecitabine.**  Tegafur is not an alternative, as this is also metabolised by DPD. | Clearance has only been determined for one patient with genotype *2A/c.2846A>T (Boisdron-Celle 2007). The clearance found for this patient was almost zero. For 1x c.2846A>T/c.2846A>T and 1x c.1236G>A/c.2846A>T, the average of the dose adjustment calculated based on 5-fluorouracil AUC was a reduction to 19% (12% for c.2846A>T/c.2846A>T and 44% for c.1236G>A/c.2846A>T) (Henricks 2017). Data on titrated or tolerated doses and on residual DPD enzyme activity showed a similar high range in values, both within as between the different genotypes. For 5 patients with c.1236G>A/c.1236G>A, the tolerated dose varied from 40% to 100% of the normal dose (Meulendijks 2016 and Henricks 2017). For one patient with c.2846A>T/c.2846A>T, the tolerated dose was 17% of the normal dose (Henricks 2017). For one patient with both c.1236G>A and c.2846A>T, the tolerated dose was 51% of the normal dose (Henricks 2017). For 4 patients with c.1236G>A/c.1236G>A, the DPD enzyme activity varied from 41% to 79% of the activity in patients without a gene variant (Meulendijks 2016 and Henricks 2017). For 2 patients with c.2846A>T/c.2846A>T, the DPD enzyme activity varied from 10% to 29% of the normal value (Henricks 2017). For one patient with both c.1236G>A and c.2846A>T, the DPD enzyme activity was 45% of the normal value (Henricks 2017). Lunenburg 2018 Genes (Basel) found 2 patients with gene activity 0.25-0.5 with null allele to tole-rate 50% of the normal dose. These patients had respectively 60% and 72% of the normal DPD enzyme activity. However, 4 patients with this phenotype who had previously developed sever toxicity on full dose fluoropyrimidines had DPD enzyme activities ranging from 1% to 38% of the normal value. The low DPD enzyme activities in these patients might be due to neutropenia, because DPD enzyme activity is measured in mononuclear cells. So in these 6 patients, the DPD activity was 33% of that measured in *1/*1-patients, but 66% if measured prior to toxicity.  Because of the high interpatient variability, the scarcity of data for calculating a dose recommendation, and the low prevalence of patients with two gene variants (0.35% of the 1138 genotyped patients in Henricks 2018 Lancet Oncol), the KNMP Pharmacogenetics Working Group recommends to fully personalise therapy in these patients, i.e. measure DPD enzyme activity and adjust the fluoropyrimidine dose accordingly.  Instead of dose personalisation, physicians may also choose an alternative. | Increase in the AUC of fluorouracil by 127% (1x c.1236G>A/c.2846A>T) or 766% (1x c.2846A>T/c.2846A>T).  Clearance decreased by almost 100% (1x *2A/c.2846A>T).  Extrapolation of the dose reductions identified for *1/*2A, *1/c.2846A>T and *1/c.1236G>A would lead to a dose reduction by 50-70% for c.1236G>A/c.1236G>A, c.1236G>A/c.2846A>T and c.2846A>T/c.2846A>T and by 75-85% for *2A/c.2846A>T. | 2 studies and a meta-analysis found an increased risk of ≥ 3 toxicity for c.1236G>A/c.1236G>A plus *1/c.1236G>A (gene activity score 1.5) versus *1/*1 or for c.1236G>A/c.1236G>A versus *1/c.1236G>A versus *1/*1. One study involving 34 patients with a gene activity score of 1.5 or PHENO, including 2 with PHENO (both c.1236G>A/c.1236G>A) found an increased risk of haematological and gastro-intestinal toxicity ≥ grade 3. One patient with *2A and c.2846A>T developed grade III/IV toxicity and died due to toxicity. A second patient with *2A and c.2846A>T developed grade V toxicity and tolerated only one cycle of FOLFOX plus cetuximab. Four patients (1x *2A and c.2846A>T, 3x *2A and 1236T) developed ≥ grade 3 toxicity (1x grade 3, 2x grade 4, 1x grade 5). Three of them were admitted to the hospital for 7 – 14 days.  Of 3 patients with genotype c.1236G>A/c.1236G>A, only one tolerated a standard dose. A second patient tolerated the treatment following dose reduction to 60% of the standard dose. In another study, 2x c.1236G>A/c.1236G>A, 1x c.1236G>A/c.2846A>T and 1x c.2846A>T/c.2846A>T tolerated an average of 55% of the standard dose, but there was strong variation between the patients (and genotypes) (17-100% of the standard dose). One patient with genotype *2A/c.2846A>T (gene variants on different alleles) received half the standard dose, but the palliative fluoropyrimidine therapy was nevertheless stopped after the first cycle due to side effects (≤ grade 3). One patient with genotype *1/*2A+c.2846A>T (gene variants on the same allele) did not develop toxicity at 50% of the standard dose.  The latter two patients had 60% and 72% of the standard DPD activity respectively. For 4 patients with c.1236G>A/c.1236G>A, the DPD activity varied from 41%-79% of the activity in patients without gene variants. For 2 patients with c.2846A>T/c.2846A>T, the DPD activity varied from 10%-29% of the standard value. For one patient with c.1236G>A and c.2846A>T, the DPD activity was 45% of the standard value. For four patients with both *2A and c.1236G>A or c.2846A>T, who had previously developed severe toxicity on the standard dose, the measured DPD activity varied from 1-38% (average 16%). As the DPD activity is determined in peripheral mononuclear cells, any residual neutropenia could potentially result in lower determined values. |
| Gene activity score 1  References:  (1, 3-6, 8-12, 14, 15, 17-45) | **Start with 50% of the standard dose or avoid fluorouracil and capecitabine.**  Adjustment of the subsequent dose should be guided by toxicity and effectiveness. However, in one study involving 17 patients with gene activity 1, the average dose after titration was 57% of the standard dose.  Tegafur is not an alternative, as this is also metabolised by DPD. | For 25x *1/*2A and 1x *1/*13, the weighted average of the dose adjustments calculated based on 5-fluorouracil clearance or AUC was a reduction to 47% (20-49%, median 46%) (Deenen 2016, Boisdron-Celle 2007 and Morel 2006). The weighted mean of 47% was translated to 50% to be more achievable in clinical practice. Several studies on tolerated doses, residual enzyme activities and a 50% starting dose for AS 1 support this dose recommendation. Titrated or tolerated doses were 56% of the normal dose in Deenen 2011 (n = 5), 61% in Van Kuilenburg 2012 (n = 9), < 50% in Magnani 2013 (n = 3), 48% in Deenen 2016 (n = 18), 53% in Lunenburg 2016 (n = 4), 57% in Henricks 2018 Lancet Oncol (n = 17) and 54% in Kleinjan 2019 (n = 4). DPD enzyme activities were 64% of that for *1/*1 in Deenen 2016 (n = 15) and 54% in Henricks 2018 Lancet Oncol (n = 9; standard deviation for *1/*2A of 8% (n = 8); median 56% (n = 8)). In addition, Henricks 2018 Int J Cancer found no differences in toxicity and efficacy between 40 *1/*2A-patients (37 for efficacy) on a starting dose of approximately 50% of the normal dose and *1/*1-patients on the normal dose. Henricks 2018 Lancet Oncol found no differences in toxicity between 17 patients with AS 1 on a star-ting dose of 50% of the normal dose and *1/*1-patients on the normal dose, despite bad tolerance being induced by a subsequent dose increase in 12% of the patients with AS 1. Deenen 2016 found no difference in toxicities between 18 patients with *1/*2A on an initial dose of maximally 50% of the normal dose and *1/*1-patients on the normal dose. Lunenburg 2016 found no grade ≥ 3 toxicity when treating three patients with *1/*2A with an initial dose of 50% of the normal dose.  Based on these data, the KNMP Pharmacogenetics Working Group recommends to start with 50% of the normal dose. If appropriate, the dose can subsequently be adjusted based on toxicity and efficacy.  Instead of dose adjustment, physicians may also choose an alternative. | Increase in the AUC of fluorouracil by 103% (16x *1/*2A).  52-80% decrease in clearance.  69-109% increase in half-life. | 8 of the 11 studies and two meta-analyses found an increased risk of grade ≥ 3 toxicity. Increased grade ≥ 3 toxicity: OR = 4.67-24.9; RR = 4.40-9.76. The highest ORs were found for haematological toxicity. There was a 74-793% increase in the percentage of patients with grade ≥ 3 toxicity. Out of 48 patients with genotype *1/*2A in published cohort studies, 73% developed grade ≥ 3 toxicity. The allele frequency of *2A in a group with grade III/IV toxicity was 1548-2879% higher. Toxicity generally occurred in the first cycle. Six patients died due to toxicity, including two that had used capecitabine.  No association with grade ≥ 3 toxicity was found for breast cancer patients receiving adjuvant/neoadjuvant therapy with fluorouracil, epirubicin and cyclophosphamide in a phase II study that showed 94% grade ≥ 3 toxicity and in a small study of 21 patients with grade ≥ 3 toxicity. Fluorouracil toxicity is not common in breast cancer patients treated with this combination therapy.  A large study found that the *2A allele only increased the risk of grade ≥ 3 toxicity in men (OR = 41.8) and not in women. Other studies did not find any differences between men and women.  When the dose was guided by toxicity, the average dose in the sixth cycle was 56% of the standard dose in 7 *1/*2A. Dose reduction down to 40% or 50% of the standard dose was not adequate in two *1/*2A patients in another study. A third study found a titrated dose of 57% of the standard dose for 16 *1/*2A plus 1 *1/*13 (after starting at 50% of the standard dose). The DPD activity in these patients was 53% of the activity in patients without a gene variation. A fourth study found a tolerated dose of 54% of the standard dose in 4 *1/*2A (after starting at 50% of the standard dose).  There was no difference in effectiveness (general survival, progression-free survival and percentage of patients with a complete or partial response or stable disease) and grade ≥ 3 toxicity between 40 *1/*2A (37 *1/*2A for effectiveness outcomes) at approximately 50% of the standard initial dose and patients without *2A on the standard initial dose. There was no difference in grade ≥ 3 toxicity between 18 *1/*2A at ≤ 50% of the standard dose and non-selected patients on the standard dose. There was no difference in grade ≥ 3 toxicity between 16 *1/*2A on 50% of the standard initial dose and patients without a gene variation on the standard dose, despite the fact that intolerance/toxicity was induced in 13% of the *1/*2A in this study by a dose increase after two cycles. In another study, 4 *1/*2A did not develop grade ≥ 3 toxicity at 50% of the standard dose. One of them had previously developed grade ≥ 3 toxicity during the first cycle at the standard dose. One of them tolerated a dose increase to 60%, the other two did not tolerate a dose increase to 80% and 100% respectively. There was no difference in grade ≥ 3 toxicity between 11 patients with a gene variation at a reduced dose – including 4 *1/*2A at 50% of the standard initial dose – and patients without a gene variation on the standard initial dose. There was no difference in grade ≥ 3 toxicity between 22 patients with a gene variation on a reduced dose – including 10 *1/*2A at 50% of the standard initial dose – and patients without a gene variation on the standard initial dose. |
| Gene activity score 1.5  References:  (3-5, 8-12, 14, 15, 17-19, 21, 22, 26, 27, 31-33, 39, 44-47) | **Start with 50% of the standard dose or avoid fluorouracil and capecitabine.**  After starting treatment, the dose should be adjusted based on toxicity and effectiveness. In a study involving 17 patients with genotype *1/c.2846A>T, the average dose after titration was 64% of the standard dose. For 51 patients with genotype *1/c.1236G>A, the average dose after titration was 74% of the standard dose.  Tegafur is not an alternative, as this is also metabolised by DPD. | For *1/c.2846A>T, the weighted average of the calculated dose adjustments was a reduction to 55%. However, Deenen 2011 investigated 8 patients with *1/c.2846A>T and found a toxicity-guided dose reduction to 76% of the standard dose. In addition, Lunenburg 2016 found no grade ≥ 3 toxicity when treating five patients with *1/c.1236G>A with an initial dose of 75% of the standard dose. Deenen 2011 did not find significantly more dose decreases for *1/c.1236G>A + c.1236G>A/c.1236G>A compared to *1/*1 when treating 568 patients with chemo-therapy with capecitabine 1000 mg/m2 twice daily. Kleinjan 2019 found the tolerated dose for 6 patients with *1/c.1236G>A to be 78% of the normal dose. The single patients with *1/c.2846A>T in Lunenburg 2018 and Kleinjan 2019 tolerated 60% and 85% of the normal dose, respectively. In a study with 51 patients with *1/c.1236G>A and 17 patients with *1/c.2846A>T, Henricks 2018 Lancet Oncol found the titrated dose to be 74% of the normal dose for *1/c.1236G>A and 64% for *1/c.2846A>T. The mean and median DPD enzyme activity in this study were respectively 80% (standard deviation 30%) and 74% of that for *1/*1 for *1/c.1236G>A, and respectively 66% (standard deviation 20%) and 67% for *1/c.2846A>T. In this study, the (planned) starting dose was 75% of the normal dose, but bad tolerance was induced in 6% of patients with *1/c.1236G>A by increasing the dose afterwards and in 12% of patients with *1/c.2846A>T, either by not applying a reduced starting dose or by increasing the dose after tolerance for the reduced starting dose. In this study, the percentage of patients with overall grade ≥ 3 toxicity was still higher for *1/236A and for *1/c.2846A>T on (planned) reduced starting dose than for *1/*1 on the normal starting dose. To avoid this additional toxicity in patients with *1/c.1236G>A and *1/c.2846A>T, the KNMP Pharmacogenetics Working Group recommends starting with a lower than the calculated dose (i.e. 50% instead of 75% or 65% of the normal dose) and subsequently adjust the dose based on toxicity and efficacy.  Instead of dose adjustment, physicians may also choose an alternative. | 40-58% decrease in clearance. | 4 of the 6 studies and two meta-analyses found an increased risk of grade ≥ 3 toxicity. One study involving 19 c.2846A>T carriers found no significantly increased risk of grade ≥ 3 toxicity for c.2846A>T , but did find an increased risk for c.2846A>T and *13 combined. This study found no increased risk of grade ≥ 3 toxicity for 58 c.1236G>A carriers, but did find a trend towards an increased risk of hospitalisation. One study found an increased risk of grade ≥ 2 toxicity. Increased grade ≥ 3 toxicity: OR = 2.2-9.35 and RR = 3.0; RR = 1.59 for gene activity score 1.5 plus PHENO (*1/c.1236G>A+c.1236G>A/c.1236G>A). The percentage of patients with grade ≥ 3 toxicity was 109-1175% higher. One *1/c.2846A>T died due to toxicity after two cycles of a standard dose. One patient with genotype *1/c.1236G>A, who started on the standard dose, developed grade 3-4 toxicity during the first cycle.  No association with grade ≥ 3 toxicity was found in one small study of 21 patients with grade ≥ 3 toxicity. 1 patient (*1/c.496A>G) died as a result of the toxicity.  When the dose for 8 *1/c.2846A>T was guided by toxicity, the average dose in the sixth cycle was 76% of the standard dose. 5 patients with genotype *1/c.1236G>A did not develop grade ≥ 3 toxicity at 75 % of the standard dose. The two patients for who the dose was then increased tolerated the standard dose. One study found a tolerated dose of 78% of the standard dose in 6 *1/c.1236G>A and 85% of the standard dose in 1 *1/c.2846A>T (after starting at 75% of the standard dose). In one study in which intolerance/toxicity was induced by dose increase or use of the standard dose from the start, in 6% of the *1/c.1236G>A and 12% of the *1/c.2846A>T, 51 *1/c.1236G>A and 17 *1/c.2846A>T had a higher incidence of grade ≥ 3 toxicity at a scheduled 75% of the standard initial dose than patients without a gene variation at the standard dose. The titrated dose for *1/c.1236G>A in this study was 74% of the standard dose and the DPD activity was an average 80% (standard deviation 30%) and median 74% of the value for patients without a gene variation. The titrated dose for *1/c.2846A>T in this study was 64% of the standard dose and the DPD activity was 66% (standard deviation 20%) and median 67% of the value for patients without a gene variation. There was no difference in grade ≥ 3 toxicity between 11 patients with a gene variation at a reduced dose – including 6 *1/c.1236G>A and 1 *1/c.2846A>T at 75% of the standard initial dose – and patients without a gene variation on the standard initial dose. There was no difference in grade ≥ 3 toxicity between 22 patients with a gene variation at a reduced dose – including 11 *1/c.1236G>A on 50-75% of the standard initial dose and 1 *1/c.2846A>T at 60% of the standard initial dose – and patients without a gene variation on the standard initial dose. |

5-FU: 5-fluorouracil; AUC: Area Under the Curve; PHENO: DPD enzyme activity cannot be predicted correctly, an additional phenotyping test is required to determine the DPD enzyme activity; DPD: dihydropyrimidine dehydrogenase; OR: Odds Ratio

References:

1. Henricks LM, Kienhuis E, de Man FM, van der Veldt AAM, Hamberg P, van Kuilenburg ABP, et al. Treatment algorithm for homozygous or compound heterozygous DPYD variant allele carriers with low dose capecitabine. JCO Precis Oncol. 2017.

2. Henricks LM, Siemerink EJM, Rosing H, Meijer J, Goorden SMI, Polstra AM, et al. Capecitabine-based treatment of a patient with a novel DPYD genotype and complete dihydropyrimidine dehydrogenase deficiency. Int J Cancer. 2018;142(2):424-30.

3. Rosmarin D, Palles C, Church D, Domingo E, Jones A, Johnstone E, et al. Genetic markers of toxicity from capecitabine and other fluorouracil-based regimens: investigation in the QUASAR2 study, systematic review, and meta-analysis. J Clin Oncol. 2014;32(10):1031-9.

4. Boisdron-Celle M, Remaud G, Traore S, Poirier AL, Gamelin L, Morel A, et al. 5-Fluorouracil-related severe toxicity: a comparison of different methods for the pretherapeutic detection of dihydropyrimidine dehydrogenase deficiency. Cancer letters. 2007;249(2):271-82.

5. Morel A, Boisdron-Celle M, Fey L, Soulie P, Craipeau MC, Traore S, et al. Clinical relevance of different dihydropyrimidine dehydrogenase gene single nucleotide polymorphisms on 5-fluorouracil tolerance. Mol Cancer Ther. 2006;5(11):2895-904.

6. Raida M, Schwabe W, Hausler P, Van Kuilenburg ABP, Van Gennip AH, Behnke D, et al. Prevalence of a common point mutation in the Dihydropyrimidine dehydrogenase (DPD) gene within the 5 '-splice donor site of intron 14 in patients with severe 5-fluorouracil (5-FU)-related toxicity compared with controls. Clinical Cancer Research. 2001;7(9):2832-9.

7. Johnson MR, Hageboutros A, Wang K, High L, Smith JB, Diasio RB. Life-threatening toxicity in a dihydropyrimidine dehydrogenase-deficient patient after treatment with topical 5-fluorouracil. Clin Cancer Res. 1999;5(8):2006-11.

8. SPC Fluorouracil PCH 15 October 2012.

9. SPC Efudix (fluorouracil) crème 07 September 2016.

10. SPC Xeloda (capecitabine) 26 July 2016.

11. SPC Fluorouracil 29 July 2016 (USA).

12. SPC Xeloda (capecitabine) 14 December 2016 (USA)

13. SPC Carac (fluorouracil) cream 16 December 2003 (USA).

14. Lunenburg CATC, van Staveren MC, Gelderblom H, Guchelaar HJ, Swen JJ. Evaluation of clinical implementation of prospective DPYD genotyping in 5-fluorouracil- or capecitabine-treated patients. Pharmacogenomics. 2016;17(7):721-9.

15. Lunenburg CATC, Henricks LM, Dreussi E, Peters FP, Fiocco M, Meulendijks D, et al. Standard fluoropyrimidine dosages in chemoradiation therapy result in an increased risk of severe toxicity in DPYD variant allele carriers. Eur J Cancer. 2018;104:210-8.

16. Lunenburg CATC, Henricks LM, van Kuilenburg ABP, Mathijssen RHJ, Schellens JHM, Gelderblom H, et al. Diagnostic and Therapeutic Strategies for Fluoropyrimidine Treatment of Patients Carrying Multiple DPYD Variants. Genes. 2018;9(12).

17. Lee AM, Shi Q, Pavey E, Alberts SR, Sargent DJ, Sinicrope FA, et al. DPYD variants as predictors of 5-fluorouracil toxicity in adjuvant colon cancer treatment (NCCTG N0147). J Natl Cancer Inst. 2014;106(12).

18. Madi A, Fisher D, Maughan TS, Colley JP, Meade AM, Maynard J, et al. Pharmacogenetic analyses of 2183 patients with advanced colorectal cancer; potential role for common dihydropyrimidine dehydrogenase variants in toxicity to chemotherapy. Eur J Cancer. 2018;102:31-9.

19. Henricks LM, Lunenburg CATC, de Man FM, Meulendijks D, Frederix GWJ, Kienhuis E, et al. DPYD genotype-guided dose individualisation of fluoropyrimidine therapy in patients with cancer: a prospective safety analysis. Lancet Oncol. 2018;19(11):1459-67.

20. Henricks LM, van Merendonk LN, Meulendijks D, Deenen MJ, Beijnen JH, de Boer A, et al. Effectiveness and safety of reduced-dose fluoropyrimidine therapy in patients carrying the DPYD*2A variant: a matched pair analysis. Int J Cancer. 2018.

21. Kleinjan JP, Brinkman I, Bakema R, van Zanden JJ, van Rooijen JM. Tolerance-based capecitabine dose escalation after DPYD genotype-guided dosing in heterozygote DPYD variant carriers: a single-center observational study. Anti-cancer drugs. 2019.

22. Meulendijks D, Henricks LM, Jacobs BAW, Aliev A, Deenen MJ, de Vries N, et al. Pretreatment serum uracil concentration as a predictor of severe and fatal fluoropyrimidine-associated toxicity. Br J Cancer. 2017;116(11):1415-24.

23. Kodali S, Bathini V, Rava P, Tipirneni E. Capecitabine-Induced Severe Toxicity Secondary to DPD Deficiency and Successful Treatment with Low Dose 5-Fluorouracil. J Gastrointest Cancer. 2016.

24. Meulendijks D, Henricks LM, van Kuilenburg AB, Jacobs BA, Aliev A, Rozeman L, et al. Patients homozygous for DPYD c.1129-5923C>G/haplotype B3 have partial DPD deficiency and require a dose reduction when treated with fluoropyrimidines. Cancer Chemother Pharmacol. 2016;78(4):875-80.

25. Deenen MJ, Meulendijks D, Cats A, Sechterberger MK, Severens JL, Boot H, et al. Upfront Genotyping of DPYD*2A to Individualize Fluoropyrimidine Therapy: A Safety and Cost Analysis. J Clin Oncol. 2016;34(3):227-34.

26. Meulendijks D, Henricks LM, Sonke GS, Deenen MJ, Froehlich TK, Amstutz U, et al. Clinical relevance of DPYD variants c.1679T>G, c.1236G>A/HapB3, and c.1601G>A as predictors of severe fluoropyrimidine-associated toxicity: a systematic review and meta-analysis of individual patient data. Lancet Oncol. 2015;16(16):1639-50.

27. Terrazzino S, Cargnin S, Del Re M, Danesi R, Canonico PL, Genazzani AA. DPYD IVS14+1G>A and 2846A>T genotyping for the prediction of severe fluoropyrimidine-related toxicity: a meta-analysis. Pharmacogenomics. 2013;14(11):1255-72.

28. Magnani E, Farnetti E, Nicoli D, Casali B, Savoldi L, Focaccetti C, et al. Fluoropyrimidine toxicity in patients with dihydropyrimidine dehydrogenase splice site variant: the need for further revision of dose and schedule. Intern Emerg Med. 2013;8(5):417-23.

29. Vulsteke C, Lambrechts D, Dieudonne A, Hatse S, Brouwers B, van Brussel T, et al. Genetic variability in the multidrug resistance associated protein-1 (ABCC1/MRP1) predicts hematological toxicity in breast cancer patients receiving (neo-)adjuvant chemotherapy with 5-fluorouracil, epirubicin and cyclophosphamide (FEC). Ann Oncol. 2013;24(6):1513-25.

30. van Kuilenburg AB, Hausler P, Schalhorn A, Tanck MW, Proost JH, Terborg C, et al. Evaluation of 5-fluorouracil pharmacokinetics in cancer patients with a c.1905+1G>A mutation in DPYD by means of a Bayesian limited sampling strategy. Clin Pharmacokinet. 2012;51(3):163-74.

31. Deenen MJ, Tol J, Burylo AM, Doodeman VD, De BA, Vincent A, et al. Relationship between single nucleotide polymorphisms and haplotypes in DPYD and toxicity and efficacy of capecitabine in advanced colorectal cancer. Clin Cancer Res. 2011;17(10):3455-68.

32. Gross E, Busse B, Riemenschneider M, Neubauer S, Seck K, Klein HG, et al. Strong association of a common dihydropyrimidine dehydrogenase gene polymorphism with fluoropyrimidine-related toxicity in cancer patients. PloS one. 2008;3(12):e4003.

33. Capitain O, Boisdron-Celle M, Poirier AL, Abadie-Lacourtoisie S, Morel A, Gamelin E. The influence of fluorouracil outcome parameters on tolerance and efficacy in patients with advanced colorectal cancer. Pharmacogenomics J. 2008;8(4):256-67.

34. Sulzyc-Bielicka V, Binczak-Kuleta A, Pioch W, Kladny J, Gziut K, Bielicki D, et al. 5-Fluorouracil toxicity-attributable IVS14 + 1G > A mutation of the dihydropyrimidine dehydrogenase gene in Polish colorectal cancer patients. Pharmacol Rep. 2008;60(2):238-42.

35. Schwab M, Zanger UM, Marx C, Schaeffeler E, Klein K, Dippon J, et al. Role of genetic and nongenetic factors for fluorouracil treatment-related severe toxicity: a prospective clinical trial by the German 5-FU Toxicity Study Group. J Clin Oncol. 2008;26(13):2131-8.

36. Jatoi A, Martenson JA, Foster NR, McLeod HL, Lair BS, Nichols F, et al. Paclitaxel, carboplatin, 5-fluorouracil, and radiation for locally advanced esophageal cancer: phase II results of preliminary pharmacologic and molecular efforts to mitigate toxicity and predict outcomes: North Central Cancer Treatment Group (N0044). Am J Clin Oncol. 2007;30(5):507-13.

37. Magne N, Etienne-Grimaldi MC, Cals L, Renee N, Formento JL, Francoual M, et al. Dihydropyrimidine dehydrogenase activity and the IVS14+1G>A mutation in patients developing 5FU-related toxicity. Br J Clin Pharmacol. 2007;64(2):237-40.

38. Saif MW, Syrigos K, Mehra R, Mattison LK, Diasio RB. Dihydropyrimidine dehydrogenase deficiency (DPD) in GI malignancies: Experience of 4-years. Pak J Med Sci. 2007;23(6):832-9.

39. Cho HJ, Park YS, Kang WK, Kim JW, Lee SY. Thymidylate synthase (TYMS) and dihydropyrimidine dehydrogenase (DPYD) polymorphisms in the Korean population for prediction of 5-fluorouracil-associated toxicity. Ther Drug Monit. 2007;29(2):190-6.

40. Salgado J, Zabalegui N, Gil C, Monreal I, Rodriguez J, Garcia-Foncillas J. Polymorphisms in the thymidylate synthase and dihydropyrimidine dehydrogenase genes predict response and toxicity to capecitabine-raltitrexed in colorectal cancer. Oncol Rep. 2007;17(2):325-8.

41. Largillier R, Etienne-Grimaldi MC, Formento JL, Ciccolini J, Nebbia JF, Ginot A, et al. Pharmacogenetics of capecitabine in advanced breast cancer patients. Clin Cancer Res. 2006;12(18):5496-502.

42. Salgueiro N, Veiga I, Fragoso M, Sousa O, Costa N, Pellon ML, et al. Mutations in exon 14 of dihydropyrimidine dehydrogenase and 5-Fluorouracil toxicity in Portuguese colorectal cancer patients. Genet Med. 2004;6(2):102-7.

43. Van Kuilenburg AB, Meinsma R, Zoetekouw L, Van Gennip AH. High prevalence of the IVS14 + 1G>A mutation in the dihydropyrimidine dehydrogenase gene of patients with severe 5-fluorouracil-associated toxicity. Pharmacogenetics. 2002;12(7):555-8.

44. van Kuilenburg AB, Haasjes J, Richel DJ, Zoetekouw L, Van Lenthe H, De Abreu RA, et al. Clinical implications of dihydropyrimidine dehydrogenase (DPD) deficiency in patients with severe 5-fluorouracil-associated toxicity: identification of new mutations in the DPD gene. Clin Cancer Res. 2000;6(12):4705-12.

45. Lee AM, Shi Q, Alberts SR, Sargent DJ, Sinicrope FA, Berenberg JL, et al. Association between DPYD c.1129-5923 C>G/hapB3 and severe toxicity to 5-fluorouracil-based chemotherapy in stage III colon cancer patients: NCCTG N0147 (Alliance). Pharmacogenet Genomics. 2016;26(3):133-7.

46. Kristensen MH, Pedersen PL, Melsen GV, Ellehauge J, Mejer J. Variants in the dihydropyrimidine dehydrogenase, methylenetetrahydrofolate reductase and thymidylate synthase genes predict early toxicity of 5-fluorouracil in colorectal cancer patients. J Int Med Res. 2010;38(3):870-83.

47. Yamaguchi K, Arai Y, Kanda Y, Akagi K. Germline mutation of dihydropyrimidine dehydrogenese gene among a Japanese population in relation to toxicity to 5-Fluorouracil. Jpn J Cancer Res. 2001;92(3):337-42.
