# Supplementary material for: Dutch Pharmacogenetics Working Group (DPWG) guideline for the gene–drug interaction of DPYD and fluoropyrimidines
Source: Eur J Hum Genet. 2019 Nov 19;28(4):508–17. doi: 10.1038/s41431-019-0540-0 (PMC7080718; doi:10.1038/s41431-019-0540-0)
Supplement: Supplementary file 6 — Dutch Pharmacogenetics Working Group (DPWG) Guideline for DPYD and tegafur with DPD inhibitors [file 41431_2019_540_MOESM6_ESM.docx]

**Supplementary Table 6:** Dutch Pharmacogenetics Working Group (DPWG) Guideline for *DPYD* and tegafur with DPD inhibitors: the therapeutic recommendation and its rationale, and the kinetic and clinical consequences for each aberrant gene activity score

| **Predicted phenotype** | **Therapeutic recommendation** | **Rationale of the therapeutic recommendation** | **Kinetic consequence** | **Clinical consequence** |
| --- | --- | --- | --- | --- |
| Gene activity score 0  References:  (1) | **Avoid tegafur**  Fluorouracil and capecitabine are not suitable alternatives, as these are also metabolised by DPD.  **If it is not possible to avoid tegafur: start with a very low dose and adjust the initial dose based on toxicity and efficacy.**  A substantiated recommendation for dose reduction cannot be made based on the literature. The recommendation for fluorouracil and capecitabine is to determine the residual DPD activity in mononuclear cells from peripheral blood and to adjust the initial dose accordingly. A patient with 0.5% of the normal DPD activity tolerated 0.8% of the standard capecitabine dose (150 mg every 5 days). A patient with undetectable DPD activity tolerated 0.43% of the standard capecitabine dose (150 mg every 5 days with every third dose skipped) | There are no data on the use of tegafur in combination with a DPD inhibitor for gene activity score 0. The SmPCs state that tegafur in combination with a DPD inhibitor is contraindicated in patients with dihydropyrimidine dehydrogenase deficiency, but do not substantiate this.  However, two patients using standard doses of tegafur-uracil who developed severe toxicity were found for each of the partially deficient phenotypes gene activity score 1 and 1.5. The toxicity was similar to that found in patients treated with capecitabine or fluorouracil, both of which are given without a DPD inhibitor.  The DPD inhibitor is 200x more potent in the tegafur-gimeracil-oteracil combination. However, fluorouracil is still metabolised by DPD after administration of this combination and DPD is therefore also involved in fluorouracil clearance.  For fluorouracil and capecitabine, the maximally tolerated dose of 50% of the normal dose for *1/*2A indicates that the maximally tolerated dose for *2A/*2A (gene activity 0) is close to zero, as do the scarce data on tole-rated doses in patients with gene activity 0. For this reason, an alternative is advised.  There is a fairly good correlation between the residual DPD enzyme activity in peripheral blood mononuclear cells and the tolerated fluorouracil or capecitabine dose. Therefore, if an alternative is not possible, adjusting the dose according to the residual DPD enzyme activity in peripheral blood mononuclear cells is advised. This strategy has been shown to be feasible for capecitabine in two patients with genotype *2A/*2A. A patient with 0.5% of the normal DPD activity tolerated 0.8% of the normal capecitabine dose (150 mg every 5 days). A patient with undetectable DPD activity, tolerated 0.43% of the normal capecitabine dose (150 mg every 5 days with every third dose skipped). This is why this strategy is also recommended for tegafur in case an alternative is not possible. | Studies regarding the kinetic consequences are unavailable. | Studies regarding the clinical consequences are unavailable. The SmPC states that this combination is contraindicated in patients with DPD deficiency. This probably refers to gene activity score 0. No safe dose for 5-FU (the metabolite of tegafur) has been found for patients assigned a gene activity score of 0. In addition, four patients with a less deficient DPD activity (assigned a gene activity score of 1 or 1.5) had a comparable toxicity for treatment with tegafur/uracil as found for treatment with 5-FU or capecitabine. |
| PHENO  References:  (1) | **Choose an alternative or start with a low dose and adjust the initial dose based on toxicity and efficacy.**  Do not choose 5-FU or capecitabine, as these are also metabolised by DPD.  A substantiated recommendation for dose reduction cannot be made based on the literature. For 5-FU and capecitabine, starting with 25% of the standard dose is recommended.  NOTE: This recommendation only applies if the two gene variations are on different alleles. If both variations are on the same allele, this patient is assigned a gene activity score of 1 and the recommendation for that gene activity score should be followed. These two situations can only be distinguished by determining the enzyme activity (phenotyping). | There are no data on the use of tegafur in combination with a DPD inhibitor for ‘phenotyping’. The SmPCs state that tegafur in combination with a DPD inhibitor is contraindicated in patients with a history of serious and unexpected reactions to fluoropyrimidine therapy, but do not substantiate this.  However, two patients using standard doses of tegafur-uracil who developed severe toxicity were found for each of the partially deficient phenotypes gene activity score 1 and 1.5. The toxicity was similar to that found in patients treated with capecitabine or fluorouracil, both of which are given without a DPD inhibitor. The recommendation for fluorouracil and capecitabine in patients in the genotype group phenotyping is to fully personalise therapy in these patients, i.e. measure DPD enzyme activity and adjust the fluoropyrimidine dose accordingly or to choose an alternative. This is why full personalisation of therapy or an alternative is also recommended for tegafur. | There are no studies into the kinetic consequences. | There are no studies into the clinical consequences of tegafur in combination with a DPD inhibitor for PHENO. However, four patients with a less strongly reduced DPD activity (gene activity score 1 or 1.5) had a comparable toxicity for treatment with tegafur/uracil as found for treatment with fluorouracil or capecitabine. In addition to this, four patients with gene activity score 1 could be treated with 90 % of the standard tegafur/uracil dose without grade 3-4 toxicity occurring. |
| Gene activity score 1.0  References:  (1-3) | **Avoid tegafur or start with a low dose and adjust the initial dose based on toxicity and efficacy**  Fluorouracil and capecitabine are not alternatives, as these are also metabolised by DPD.  It is not possible to offer substantiated advice for dose reduction based on the literature. For fluorouracil and capecitabine, starting with 50 % of the standard dose is recommended. | Treatment with tegafur in combination with the DPD inhibitor uracil in two patients with gene activity score 1 led to similar toxicity as found after treatment with fluorouracil or capecitabine. However, four patients with gene activity score 1 could be treated with 90% of the standard tegafur-uracil dose without grade 3-4 toxicity occurring. Similar to data found for fluorouracil and capecitabine, treatment with a reduced dose of tegafur-uracil seems possible for patients with gene activity score 1. This is why a dose reduction or alternative is recommended. | There are no studies into the kinetic consequences. | In a study, two patients had a comparable toxicity for treatment with tegafur/uracil as found for treatment with 5-FU or capecitabine. In another study, four patients could be treated with 90 % of the standard tegafur/uracil dose without grade 3-4 toxicity occurring. All six patients had the genotype *1/*2A. |
| Gene activity score 1.5  References:  (1, 3) | **Avoid tegafur or start with a low dose and adjust the initial dose based on toxicity and efficacy**  Fluorouracil and capecitabine are not alternatives, as these are also metabolised by DPD.  It is not possible to offer substantiated advice for dose reduction based on the literature. For fluorouracil and capecitabine, starting with 50 % of the standard dose is recommended and the dose should then be adjusted based on toxicity and effectiveness. In one study, the average dose of fluorouracil/capecitabine after titration was 64% of the standard dose for 17 patients with genotype *1/c.2846A>T and 74% of the standard dose for 51 patients with genotype *1/c.1236G>A**.** | Treatment with tegafur in combination with the DPD inhibitor uracil in two patients with gene activity score 1.5 (one with genotype *1/c.1236G>A and one with genotype *1/c.2846A>T) led to similar toxicity as found after treatment with fluorouracil or capecitabine. However, four patients with the more deficient phenotype gene activity score 1 could be treated with 90% of the standard tegafur-uracil dose without grade 3-4 toxicity occurring. Similar to data found for fluorouracil and capecitabine, treatment with a reduced dose of tegafur-uracil seems possible for patients with gene activity score 1 or higher. This is why a dose reduction or alternative is recommended. | There are no studies into the kinetic consequences. | Two patients with gene activity score 1.5 (1x *1/c.1236G>A and 1x *1/c.2846A>T) had a comparable toxicity for treatment with tegafur/uracil as found for treatment with fluorouracil or capecitabine. Four patients with gene activity score 1 could be treated with 90 % of the standard tegafur/uracil dose without grade 3-4 toxicity occurring. |

5-FU: 5-fluorouracil; AUC: Area Under the Curve; DPD: dihydropyrimidine dehydrogenase; PHENO: DPD enzyme activity cannot be predicted correctly, an additional phenotyping test is required to determine the DPD enzyme activity; OR: Odds Ratio

References:

1. SPC Teysuno (tegafur/gimeracil/oteracil) 05 April 2017.

2. Cubero DI, Cruz FM, Santi P, Silva ID, Del GA. Tegafur-uracil is a safe alternative for the treatment of colorectal cancer in patients with partial dihydropyrimidine dehydrogenase deficiency: a proof of principle. Ther Adv Med Oncol. 2012;4(4):167-72.

3. Deenen MJ, Terpstra WE, Cats A, Boot H, Schellens JH. Standard-dose tegafur combined with uracil is not safe treatment after severe toxicity from 5-fluorouracil or capecitabine. Ann Intern Med. 2010;153(11):767-8.
