# Supplementary material for: Dutch Pharmacogenetics Working Group (DPWG) guideline for the gene–drug interaction of DPYD and fluoropyrimidines
Source: Eur J Hum Genet. 2019 Nov 19;28(4):508–17. doi: 10.1038/s41431-019-0540-0 (PMC7080718; doi:10.1038/s41431-019-0540-0)
Supplement: Supplementary file 7 — Suggested clinical decision support texts for various health care professionals for 5-FU/capecitabine [file 41431_2019_540_MOESM7_ESM.docx]

**Supplementary Table 7:** Suggested clinical decision support texts for various health care professionals for 5-FU/capecitabine

| **Gene activity score 0: 5-fluorouracil (5-FU)/capecitabine, SYSTEMIC**  **Pharmacist text / Hospital text / Prescriber text**  The gene variation increases the risk of severe, potentially fatal toxicity. A reduced conversion of fluorouracil/capecitabine to inactive metabolites means that the standard dose is a more than 100-fold overdose.   - Avoid fluorouracil and capecitabine   Tegafur is not an alternative, as this is also metabolised by DPD.  If it is not possible to avoid fluorouracil and capecitabine: determine the residual DPD activity in mononuclear cells from peripheral blood and adjust the initial dose accordingly.  A patient with 0.5% of the normal DPD activity tolerated 0.8% of the standard dose (150 mg capecitabine every 5 days). A patient with undetectable DPD activity tolerated 0.43% of the standard dose (150 mg capecitabine every 5 days with every third dose skipped)  **Background information**  Mechanism: 5-Fluorouracil and its prodrug capecitabine are mainly converted by dihydropyrimidine dehydrogenase (DPD) to inactive metabolites. Genetic variations result in reduced DPD activity and thereby to reduced conversion of 5-fluorouracil to inactive metabolites. As a result, the intracellular concentration of the active metabolite of 5-fluorouracil can increase, resulting in severe, potentially fatal toxicity. For more information about the phenotype gene activity score 0: see the general background information about DPD on the KNMP Knowledge Bank or on www.knmp.nl (search for DPD).   Clinical consequences: All patients with gene activity score 0 with known toxicity (n=2, *2A/*2A), had grade III/IV toxicity and 50% died due to toxicity. Moreover, a patient with *2A/*2A developed severe toxicity after treatment with cutaneous fluorouracil cream.   Kinetic consequences: For 2 patients with gene activity score 0 (genotypes *2A/(duplication of exon 17 and 18) and *2A/*2A) the dose-corrected AUC of fluourouracil increased by a factor 113 and 138 respectively after the first systemic capecitabine dose.  Extrapolation of the decrease in clearance by 50% identified for *1/*2A would suggest a clearance of 0% for *2A/*2A (gene activity score 0). This is equivalent to severe toxicity found in one patient with *2A/*2A after using fluorouracil cream on the scalp and the two previously described patients using very low tolerated systemic doses (0.8% and 0.43% of the standard dose).  **Literature**   1. Henricks LM et al. Capecitabine-based treatment of a patient with a novel DPYD genotype and complete dihydropyrimidine dehydrogenase deficiency. Int J Cancer 2018;142:424-30. 2. Henricks LM et al. Treatment algorithm for homozygous or compound heterozygous DPYD variant allele carriers with low-dose capecitabine. JCO Precis Oncol - published online 2017 Oct 6. 3. Kodali S et al. Capecitabine-induced severe toxicity secondary to DPD deficiency and successful treatment with low dose 5-fluorouracil. J Gastrointest Cancer 2017;48:66-69. 4. Meulendijks D et al. Patients homozygous for DPYD c.1129-5923C>G/ haplotype B3 have partial DPD deficiency and require a dose reduction when treated with fluoropyrimidines. Cancer Chemother Pharmacol 2016;78:875-80. 5. Lunenburg CA et al. Evaluation of clinical implementation of prospective DPYD genotyping in 5-fluorouracil- or capecitabine-treated patients. Pharmacogenomics 2016;17:721-9. 6. Deenen MJ et al. Upfront genotyping of DPYD*2A to individualize fluoropyrimidine therapy: a safety and cost analysis. J Clin Oncol 2016;34:227-34. 7. Rosmarin D et al. Genetic markers of toxicity from capecitabine and other fluorouracil-based regimens: investigation in the QUASAR2 study, systematic review, and meta-analysis. J Clin Oncol 2014; 32:1031-9. 8. Deenen MJ et al. Relationship between single nucleotide polymorphisms and haplotypes in DPYD and toxicity and efficacy of capecitabine in advanced colorectal cancer. Clin Cancer Res 2011; 17:3455-68. 9. Gross E et al. Strong association of a common dihydropyrimidine dehydrogenase gene polymorphism with fluoropyrimidine-related toxicity in cancer patients. PLoS ONE 2008;3:e4003. 10. Boisdron-Celle M et al. 5-Fluorouracil-related severe toxicity: a comparison of different methods for the pretherapeutic detection of dihydropyrimidine dehydrogenase deficiency. Cancer Lett 2007;249:271-82. 11. Morel A et al. Clinical relevance of different dihydropyrimidine dehydrogenase gene single nucleotide polymorphisms on 5-fluorouracil tolerance. Mol Cancer Ther 2006;5:2895-904. 12. Van Kuilenburg AB et al. High prevalence of the IVS14 + 1G>A mutation in the dihydropyrimidine dehydrogenase gene of patients with severe 5-fluorouracil-associated toxicity. Pharmacogenetics 2002;12:555-8. 13. Raida M et al. Prevalence of a common point mutation in the dihydropyrimidine dehydrogenase (DPD) gene within the 5'-splice donor site of intron 14 in patients with severe 5-fluorouracil (5-FU)- related toxicity compared with controls. Clin Cancer Res 2001;7:2832-9. 14. van Kuilenburg AB et al. Clinical implications of dihydropyrimidine dehydrogenase (DPD) deficiency in patients with severe 5-fluorouracil-associated toxicity: identification of new mutations in the DPD gene. Clin Cancer Res 2000;6:4705-12. 15. Johnson MR et al. Life-threatening toxicity in a dihydropyrimidine dehydrogenase-deficient patient after treatment with topical 5-fluorouracil. Clin Cancer Res 1999;5:2006-11. 16. SPC’s Fluorouracil PCH, Xeloda, Efudix crème, Fluorouracil (VS), Xeloda (VS) en Carac cream (VS). |
| --- |

| **Gene activity score 0: 5-fluorouracil (5-FU) CUTANEOUS**  **Pharmacist text/ Hospital text / Prescriber text**  The gene variation increases the risk of severe, potentially fatal toxicity. A reduced conversion of fluorouracil/capecitabine to inactive metabolites means that the normal dose is an overdose.   - Avoid fluorouracil NOTE: If a patient has two different genetic variations that lead to a non-functional DPD enzyme (e.g. *2A and *13), this recommendation only applies if the variations are on a different allele. If both variations are on the same allele, this patient actually has a gene activity score 1, for which no increased risk of severe, potentially fatal toxicity has been found with cutaneous use. These two situations can only be distinguished by determining the enzyme activity (phenotyping). This recommendation only applies if the patient has virtually no enzyme activity.   **Background information**  Mechanism: 5-Fluorouracil is mainly converted by dihydropyrimidine dehydrogenase (DPD) to inactive metabolites. Genetic variations result in reduced DPD activity and thereby to reduced conversion of 5-fluorouracil to inactive metabolites. As a result, the intracellular concentration of the active metabolite of 5-fluorouracil can increase, resulting in severe, potentially fatal toxicity.  For more information about the phenotype gene activity score 0: see the general background information about DPD on the KNMP Knowledge Bank or on www.knmp.nl (search for DPD).  Clinical consequences: A patient with *2A*/**2A developed severe toxicity after treatment with cutaneous 5-fluorouracil cream. All patients using systemic 5-fluorouracil with gene activity score 0 with known toxicity (n=2, both *2A*/**2A), had grade III/IV toxicity and 50% died due to toxicity.  Kinetic consequences: For 2 patients with a gene activity score 0 (genotypes *2A/(duplication of exon 17 and 18) and *2A/*2A) the dose-corrected AUC of fluourouracil increased by a factor 113 and 138 respectively after the first systemic capecitabine dose.  Extrapolation of the decrease in clearance by 50% identified for *1/*2A would suggest a clearance of 0% for *2A/*2A (gene activity score 0). This is equivalent to severe toxicity found in one patient with *2A/*2A after using fluorouracil cream on the scalp and the two previously described patients using very low tolerated systemic doses (0.8% and 0.43% of the standard dose).  **Literature:**   1. Henricks LM et al. Capecitabine-based treatment of a patient with a novel DPYD genotype and complete dihydropyrimidine dehydrogenase deficiency. Int J Cancer 2018;142:424-30. 2. Henricks LM et al. Treatment algorithm for homozygous or compound heterozygous DPYD variant allele carriers with low-dose capecitabine. JCO Precis Oncol - published online 2017 Oct 6. 3. Kodali S et al. Capecitabine-induced severe toxicity secondary to DPD deficiency and successful treatment with low dose 5-fluorouracil. J Gastrointest Cancer 2017;48:66-69. 4. Lunenburg CA et al. Evaluation of clinical implementation of prospective DPYD genotyping in 5-fluorouracil- or capecitabine-treated patients. Pharmacogenomics 2016;17:721-9. 5. Deenen MJ et al. Upfront genotyping of DPYD*2A to individualize fluoropyrimidine therapy: a safety and cost analysis. J Clin Oncol 2016;34:227-34. 6. Rosmarin D et al. Genetic markers of toxicity from capecitabine and other fluorouracil-based regimens: investigation in the QUASAR2 study, systematic review, and meta-analysis. J Clin Oncol 2014; 32:1031-9. 7. Deenen MJ et al. Relationship between single nucleotide polymorphisms and haplotypes in DPYD and toxicity and efficacy of capecitabine in advanced colorectal cancer. Clin Cancer Res 2011; 17:3455-68. 8. Gross E et al. Strong association of a common dihydropyrimidine dehydrogenase gene polymorphism with fluoropyrimidine-related toxicity in cancer patients. PLoS ONE 2008;3:e4003. 9. Boisdron-Celle M et al. 5-Fluorouracil-related severe toxicity: a comparison of different methods for the pretherapeutic detection of dihydropyrimidine dehydrogenase deficiency. Cancer Lett 2007;249:271-82. 10. Morel A et al. Clinical relevance of different dihydropyrimidine dehydrogenase gene single nucleotide polymorphisms on 5-fluorouracil tolerance. Mol Cancer Ther 2006;5:2895-904. 11. Van Kuilenburg AB et al. High prevalence of the IVS14 + 1G>A mutation in the dihydropyrimidine dehydrogenase gene of patients with severe 5-fluorouracil-associated toxicity. Pharmacogenetics 2002;12:555-8. 12. Raida M et al. Prevalence of a common point mutation in the dihydropyrimidine dehydrogenase (DPD) gene within the 5'-splice donor site of intron 14 in patients with severe 5-fluorouracil (5-FU)-related toxicity compared with controls. Clin Cancer Res 2001;7:2832-9. 13. van Kuilenburg AB et al. Clinical implications of dihydropyrimidine dehydrogenase (DPD) deficiency in patients with severe 5-fluorouracil-associated toxicity: identification of new mutations in the DPD gene. Clin Cancer Res 2000;6:4705-12. 14. Johnson MR et al. Life-threatening toxicity in a dihydropyrimidine dehydrogenase-deficient patient after treatment with topical 5-fluorouracil. Clin Cancer Res 1999;5:2006-11. 15. SPC Efudix crème en Carac cream (VS). |
| --- |

| **PHENO: 5-fluorouracil (5-FU)/capecitabine**  **Pharmacist text / Hospital text / Prescriber text**  The gene variation increases the risk of severe, potentially fatal toxicity. A reduced conversion of fluorouracil/capecitabine to inactive metabolites means that the normal dose is an overdose.   - It is not possible to recommend a dose adjustment for these patients based on the genotype only.   Determine the residual DPD activity in mononuclear cells from peripheral blood and adjust the initial dose based on phenotype and genotype, or avoid fluorouracil and capecitabine.  Tegafur is not an alternative, as this is also metabolised by DPD.  **Background information**  Mechanism: 5-Fluorouracil and its prodrug capecitabine are mainly converted by dihydropyrimidine dehydrogenase (DPD) to inactive metabolites. Genetic variations result in reduced DPD activity and thereby to reduced conversion of 5-fluorouracil to inactive metabolites. As a result, the intracellular concentration of the active metabolite of 5-fluorouracil can increase, resulting in severe, potentially fatal toxicity. For more information about the phenotype ”phenotyping”: see the general background information about DPD on the KNMP Knowledge Bank or on www.knmp.nl (search for DPD).  Clinical consequences: 2 studies and a meta-analysis found an increased risk of ≥ 3 toxicity for c.1236G>A/c.1236G>A plus *1/c.1236G>A (gene activity score 1.5) versus *1/*1 or for c.1236G>A/c.1236G>A versus *1/c.1236G>A versus *1/*1. One study involving 34 patients with a gene activity score of 1.5 or PHENO, including 2 with PHENO (both c.1236G>A/c.1236G>A) found an increased risk of haematological and gastro-intestinal toxicity ≥ grade 3. One patient with *2A and c.2846A>T developed grade III/IV toxicity and died due to toxicity. A second patient with *2A and c.2846A>T developed grade V toxicity and tolerated only one cycle of FOLFOX plus cetuximab. Four patients (1x *2A and c.2846A>T, 3x *2A and c.1236G>A) developed ≥ grade 3 toxicity (1x grade 3, 2x grade 4, 1x grade 5). Three of them were admitted to the hospital for 7 – 14 days.  Of 3 patients with genotype c.1236G>A/c.1236G>A, only one tolerated a standard dose. A second patient tolerated the treatment following dose reduction to 60% of the standard dose. In another study, 2x c.1236G>A/c.1236G>A, 1x c.1236G>A/c.2846A>T and 1x c.2846A>T/c.2846A>T tolerated an average of 55% of the standard dose, but there was strong variation between the patients (and genotypes) (17-100% of the standard dose). One patient with genotype *2A/c.2846A>T (gene variants on different alleles) received half the standard dose, but the palliative fluoropyrimidine therapy was nevertheless stopped after the first cycle due to side effects (≤ grade 3). One patient with genotype *1/*2A+c.2846A>T (gene variants on the same allele) did not develop toxicity at 50% of the standard dose.  The latter two patients had 60% and 72% of the standard DPD activity respectively. For 4 patients with c.1236G>A/c.1236G>A, the DPD activity varied from 41%-79% of the activity in patients without gene variants. For 2 patients with c.2846A>T/c.2846A>T, the DPD activity varied from 10%-29% of the standard value. For one patient with c.1236G>A and c.2846A>T, the DPD activity was 45% of the standard value. For four patients with both *2A and c.1236G>A or c.2846A>T, who had previously developed severe toxicity on the standard dose, the measured DPD activity varied from 1-38% (average 16%). As the DPD activity is determined in peripheral mononuclear cells, any residual neutropaenia could potentially result in lower determined values.   Kinetic consequences: Increase in the AUC of fluorouracil by 127% (1x c.1236G>A/c.2846A>T) or 766% (1x c.2846A>T/c.2846A>T).  Clearance decreased by almost 100% (1x *2A/c.2846A>T).  Extrapolation of the dose reductions identified for *1/*2A, *1/c.2846A>T and *1/c.1236G>A would lead to a dose reduction by 50-70% for c.1236G>A/c.1236G>A, c.1236G>A/c.2846A>T and c.2846A>T/c.2846A>T and by 75-85% for *2A/c.2846A>T.  **Literature**   1. Lunenburg CATC et al. Diagnostic and therapeutic strategies for fluoropyrimidine treatment of patients carrying multiple DPYD variants. Genes (Basel) 2018;9:E585. 2. Lunenburg CATC et al. Standard fluoropyrimidine dosages in chemoradiation therapy result in an increased risk of severe toxicity in DPYD variant allele carriers. Eur J Cancer 2018;104:210-8. 3. Henricks LM et al. DPYD genotype-guided dose individualisation of fluoropyrimidine therapy in patients with cancer: a prospective safety analysis. Lancet Oncol 2018;19:1459-67 en persoonlijke communicatie (getitreerde dosis en mediane DPD-activiteit). 4. Henricks LM et al. Treatment algorithm for homozygous or compound heterozygous DPYD variant allele carriers with low-dose capecitabine. JCO Precis Oncol 2017 - published online Oct 6. 5. Kodali S et al. Capecitabine-induced severe toxicity secondary to DPD deficiency and successful treatment with low dose 5-fluorouracil. J Gastrointest Cancer 2017;48:66-69. 6. Meulendijks D et al. Patients homozygous for DPYD c.1129-5923C>G/ haplotype B3 have partial DPD deficiency and require a dose reduction when treated with fluoropyrimidines. Cancer Chemother Pharmacol 2016;78:875-80. 7. Lunenburg CA et al. Evaluation of clinical implementation of prospective DPYD genotyping in 5-fluorouracil- or capecitabine-treated patients. Pharmacogenomics 2016;17:721-9. 8. Lee AM et al. Association between DPYD c.1129-5923 C>G/hapB3 and severe toxicity to 5-fluorouracil-based chemotherapy in stage III colon cancer patients: NCCTG N0147 (Alliance). Pharmacogenet Genomics 2016;26:133-7. 9. Deenen MJ et al. Upfront genotyping of DPYD*2A to individualize fluoropyrimidine therapy: a safety and cost analysis. J Clin Oncol 2016;34:227-34. 10. Meulendijks D et al. Clinical relevance of DPYD variants c.1679T>G, c.1236G>A/HapB3, and c.1601G>A as predictors of severe fluoropyrimidine-associated toxicity: a systematic review and meta-analysis of individual patient data. Lancet Oncol 2015;16:1639-50. 11. Lee AM et al. DPYD variants as predictors of 5-fluorouracil toxicity in adjuvant colon cancer treatment (NCCTG N0147). J Natl Cancer Inst 2014;106:dju298. 12. Deenen MJ et al. Relationship between single nucleotide polymorphisms and haplotypes in DPYD and toxicity and efficacy of capecitabine in advanced colorectal cancer. Clin Cancer Res 2011; 17:3455-68. 13. Boisdron-Celle M et al. 5-Fluorouracil-related severe toxicity: a comparison of different methods for the pretherapeutic detection of dihydropyrimidine dehydrogenase deficiency. Cancer Lett 2007;249:271-82. 14. Morel A et al. Clinical relevance of different dihydropyrimidine dehydrogenase gene single nucleotide polymorphisms on 5-fluorouracil tolerance. Mol Cancer Ther 2006;5:2895-904. 15. SPC’s Fluorouracil PCH, Xeloda, Efudix crème, Fluorouracil (VS) en Xeloda (VS). |
| --- |

| **Gene activity score 1: 5-fluorouracil (5-FU)/capecitabine**  **Pharmacist text / Hospital text / Prescriber text**  The gene variation increases the risk of severe, potentially fatal toxicity. A reduced conversion of fluorouracil/capecitabine to inactive metabolites means that the normal dose is an overdose.   - Start with 50% of the standard dose or avoid fluorouracil and capecitabine.  Adjustment of the subsequent dose should be guided by toxicity and effectiveness. However, in one study involving 17 patients with gene activity 1, the average dose after titration was 57% of the standard dose.  Tegafur is not an alternative, as this is also metabolised by DPD.   **Background information**  Mechanism: 5-Fluorouracil and its prodrug capecitabine are mainly converted by dihydropyrimidine dehydrogenase (DPD) to inactive metabolites. Genetic variations result in reduced DPD activity and thereby to reduced conversion of 5-fluorouracil to inactive metabolites. As a result, the intracellular concentration of the active metabolite of 5-fluorouracil can increase, resulting in severe, potentially fatal toxicity. For more information about the phenotype gene activity score 1: see the general background information about DPD on the KNMP Knowledge Bank or on www.knmp.nl (search for DPD).   Clinical consequences: 8 of the 11 studies and two meta-analyses found an increased risk of grade ≥ 3 toxicity. Increased grade ≥ 3 toxicity: OR = 4.67-24.9; RR = 4.40-9.76. The highest ORs were found for haematological toxicity. There was a 74-793% increase in the percentage of patients with grade ≥ 3 toxicity. Out of 48 patients with genotype *1/*2A in published cohort studies, 73% developed grade ≥ 3 toxicity. The allele frequency of *2A in a group with grade III/IV toxicity was 1548-2879% higher. Toxicity generally occurred in the first cycle. Six patients died due to toxicity, including two that had used capecitabine.  No association with grade ≥ 3 toxicity was found for breast cancer patients receiving adjuvant/neoadjuvant therapy with fluorouracil, epirubicin and cyclophosphamide in a phase II study that showed 94% grade ≥ 3 toxicity and in a small study of 21 patients with grade ≥ 3 toxicity. Fluorouracil toxicity is not common in breast cancer patients treated with this combination therapy.  A large study found that the *2A allele only increased the risk of grade ≥ 3 toxicity in men (OR = 41.8) and not in women. Other studies did not find any differences between men and women.  When the dose was guided by toxicity, the average dose in the sixth cycle was 56% of the standard dose in 7 *1/*2A. Dose reduction down to 40% or 50% of the standard dose was not adequate in two *1/*2A patients in another study. A third study found a titrated dose of 57% of the standard dose for 16 *1/*2A plus 1 *1/*13 (after starting at 50% of the standard dose). The DPD activity in these patients was 53% of the activity in patients without a gene variation. A fourth study found a tolerated dose of 54% of the standard dose in 4 *1/*2A (after starting at 50% of the standard dose).  There was no difference in effectiveness (general survival, progression-free survival and percentage of patients with a complete or partial response or stable disease) and grade ≥ 3 toxicity between 40 *1/*2A (37 *1/*2A for effectiveness outcomes) at approximately 50% of the standard initial dose and patients without *2A on the standard initial dose. There was no difference in grade ≥ 3 toxicity between 18 *1/*2A at ≤ 50% of the standard dose and non-selected patients on the standard dose. There was no difference in grade ≥ 3 toxicity between 16 *1/*2A on 50% of the standard initial dose and patients without a gene variation on the standard dose, despite the fact that intolerance/toxicity was induced in 13% of the *1/*2A in this study by a dose increase after two cycles. In another study, 4 *1/*2A did not develop grade ≥ 3 toxicity at 50% of the standard dose. One of them had previously developed grade ≥ 3 toxicity during the first cycle at the standard dose. One of them tolerated a dose increase to 60%, the other two did not tolerate a dose increase to 80% and 100% respectively. There was no difference in grade ≥ 3 toxicity between 11 patients with a gene variation at a reduced dose – including 4 *1/*2A at 50% of the standard initial dose – and patients without a gene variation on the standard initial dose. There was no difference in grade ≥ 3 toxicity between 22 patients with a gene variation on a reduced dose – including 10 *1/*2A at 50% of the standard initial dose – and patients without a gene variation on the standard initial dose.    Kinetic consequences: Increase in the AUC of fluorouracil by 103% (16x *1/*2A).  52-80% decrease in clearance.  69-109% increase in half-life.  **Literature**   1. Kleinjan JP et al. Tolerance-based capecitabine dose escalation after DPYD genotype-guided dosing in heterozygote DPYD variant carriers: a single-center observational study. Anticancer Drugs 2019 Jan 8 [Epub ahead of print]. 2. Henricks LM et al. Effectiveness and safety of reduced-dose fluoropyrimidine therapy in patients carrying the DPYD*2A variant: a matched pair analysis. Int J Cancer 2018 Nov 28 [Epub ahead of print]. 3. Lunenburg CATC et al. Standard fluoropyrimidine dosages in chemoradiation therapy result in an increased risk of severe toxicity in DPYD variant allele carriers. Eur J Cancer 2018;104:210-8. 4. Henricks LM et al. DPYD genotype-guided dose individualisation of fluoropyrimidine therapy in patients with cancer: a prospective safety analysis. Lancet Oncol 2018;19:1459-67 en persoonlijke communicatie (getitreerde dosis en mediane DPD-activiteit). 5. Madi A et al. Pharmacogenetic analyses of 2183 patients with advanced colorectal cancer; potential role for common dihydropyrimidine dehydrogenase variants in toxicity to chemotherapy. Eur J Cancer 2018;102:31-9. 6. Meulendijks D et al. Pretreatment serum uracil concentration as a predictor of severe and fatal fluoropyrimidine-associated toicity. Br J Cancer 2017;116:1415-24. 7. Kodali S et al. Capecitabine-induced severe toxicity secondary to DPD deficiency and successful treatment with low dose 5-fluorouracil. J Gastrointest Cancer 2017;48:66-69. 8. Meulendijks D et al. Patients homozygous for DPYD c.1129-5923C>G/ haplotype B3 have partial DPD deficiency and require a dose reduction when treated with fluoropyrimidines. Cancer Chemother Pharmacol 2016;78:875-80. 9. Lunenburg CA et al. Evaluation of clinical implementation of prospective DPYD genotyping in 5-fluorouracil- or capecitabine-treated patients. Pharmacogenomics 2016;17:721-9. 10. Deenen MJ et al. Upfront genotyping of DPYD*2A to individualize fluoropyrimidine therapy: a safety and cost analysis. J Clin Ocol 2016;34:227-34. 11. Meulendijks D et al. Clinical relevance of DPYD variants c.1679T>G, c.1236G>A/HapB3, and c.1601G>A as predictors of severe fluoropyrimidine-associated toxicity: a systematic review and meta-analysis of individual patient data. Lancet Oncol 2015;16:1639-50. 12. Lee AM et al. DPYD variants as predictors of 5-fluorouracil toxicity in adjuvant colon cancer treatment (NCCTG N0147). J Natl Cancer Inst 2014;106:dju298. 13. Rosmarin D et al. Genetic markers of toxicity from capecitabine and other fluorouracil-based regimens: investigation in the QUASAR2 study, systematic review, and meta-analysis. J Clin Oncol 2014; 32:1031-9. 14. Terrazzino S et al. DPYD IVS14+1 G>A and 2846A>T genotyping for the prediction of severe fluoropyrimidine-related toxicity: a meta-analysis. Pharmacogenomics 2013;14:1255-72. 15. Magnani E et al. Fluoropyrimidine toxicity in patients with dihydropyrimidine dehydrogenase splice site variant: the need for further revision of dose and schedule. Intern Emerg Med 2013; 8:417-23. 16. Vulsteke C et al. Genetic variability in the multidrug resistance associated protein-1 (ABCC1/MRP1) predicts hematological toxicity in breast cancer patients receiving (neo-)adjuvant chemotherapy with 5-fluorouracil, epirubicin and cyclophosphamide (FEC). Ann Oncol 2013; 24:1513-25. 17. van Kuilenburg AB et al. Evaluation of 5-fluorouracil pharmacokinetics in cancer patients with a c.1905+1 G>A mutation in DPYD by means of a Bayesian limited sampling strategy. Clin Pharmacokinet 2012;51:163-74. 18. Deenen MJ et al. Relationship between single nucleotide polymorphisms and haplotypes in DPYD and toxicity and efficacy of capecitabine in advanced colorectal cancer. Clin Cancer Res 2011; 17:3455-68. 19. Gross E et al. Strong association of a common dihydropyrimidine dehydrogenase gene polymorphism with fluoropyrimidine-related toxicity in cancer patients. PLoS ONE 2008;3:e4003. 20. Capitain O et al. The influence of fluorouracil outcome parameters on tolerance and efficacy in patients with advanced colorectal cancer. Pharmacogenomics J 2008;8:256-67. 21. Sulzyc-Bielicka V et al. 5-Fluorouracil toxicity-attributable IVS14 + 1G > A mutation of the dihydropyrimidine dehydrogenase gene in Polish colorectal cancer patients. Pharmacol Rep 2008;60:238-42. 22. Schwab M et al. Role of genetic and nongenetic factors for fluorouracil treatment-related severe toxicity: a prospective clinical trial by the German 5-FU Toxicity Study Group. J Clin Oncol 2008;26:2131-8. 23. Mercier C et al. Prospective phenotypic screening for DPD deficiency prior to 5-FU administration: Decrease in toxicity, not in efficacy. J Clin Oncol 2008;26(May 20 suppl):abstr 14556. (meeting abstract) 24. Jatoi A et al. Paclitaxel, carboplatin, 5-fluorouracil, and radiation for locally advanced esophageal cancer: phase II results of preliminary pharmacologic and molecular efforts to mitigate toxicity and predict outcomes: North Central Cancer Treatment Group (N0044). Am J Clin Oncol 2007;30:507-13. 25. Magné N et al. Dihydropyrimidine dehydrogenase activity and the IVS14+1G>A mutation in patients developing 5FU-related toxicity. Br J Clin Pharmacol 2007;64:237-40. 26. Saif MW et al. Dihydropyrimidine dehydrogenase deficiency (GPD) in GI malignancies: experience of 4-years. Pak J Med Sci Q 2007;23:832-9. 27. Boisdron-Celle M et al. 5-Fluorouracil-related severe toxicity: a comparison of different methods for the pretherapeutic detection of dihydropyrimidine dehydrogenase deficiency. Cancer Lett 2007;249:271-82. 28. Cho HJ et al. Thymidylate synthase (TYMS) and dihydropyrimidine dehydrogenase (DPYD) polymorphisms in the Korean population for prediction of 5-fluorouracil-associated toxicity. Ther Drug Monit 2007;29:190-6. 29. Salgado J et al. Polymorphisms in the thymidylate synthase and dihydropyrimidine dehydrogenase genes predict response and toxicity to capecitabine-raltitrexed in colorectal cancer. Oncol Rep 2007;17:325-8. 30. Morel A et al. Clinical relevance of different dihydropyrimidine dehydrogenase gene single nucleotide polymorphisms on 5-fluorouracil tolerance. Mol Cancer Ther 2006;5:2895-904. 31. Largillier R et al. Pharmacogenetics of capecitabine in advanced breast cancer patients. Clin Cancer Res 2006;12:5496-502. 32. Salgueiro N et al. Mutations in exon 14 of dihydropyrimidine dehydrogenase and 5-Fluorouracil toxicity in Portuguese colorectal cancer patients. Genet Med 2004;6:102-7. 33. Van Kuilenburg AB et al. High prevalence of the IVS14 + 1G>A mutation in the dihydropyrimidine dehydrogenase gene of patients with severe 5-fluorouracil-associated toxicity. Pharmacogenetics 2002;12:555-8. 34. Raida M et al. Prevalence of a common point mutation in the dihydropyrimidine dehydrogenase (DPD) gene within the 5'-splice donor site of intron 14 in patients with severe 5-fluorouracil (5-FU)- related toxicity compared with controls. Clin Cancer Res 2001;7:2832-9. 35. van Kuilenburg AB et al. Clinical implications of dihydropyrimidine dehydrogenase (DPD) deficiency in patients with severe 5-fluorouracil-associated toxicity: identification of new mutations in the DPD gene. Clin Cancer Res 2000;6:4705-12. 36. SPC’s Fluorouracil PCH, Xeloda, Efudix crème, Fluorouracil (VS) en Xeloda (VS). |
| --- |

| **Gene activity score 1.5: 5-fluorouracil (5-FU)/capecitabine**  **Pharmacist text / Hospital text / Prescriber text**  The gene variation increases the risk of severe, potentially fatal toxicity. A reduced conversion of fluorouracil/capecitabine to inactive metabolites means that the normal dose is an overdose.   - Start with 50% of the standard dose or avoid fluorouracil and capecitabine. After starting treatment, the dose should be adjusted based on toxicity and effectiveness. In a study involving 17 patients with genotype *1/c.2846A>T, the average dose after titration was 64% of the standard dose. For 51 patients with genotype *1/c.1236G>A, the average dose after titration was 74% of the standard dose. Tegafur is not an alternative, as this is also metabolised by DPD.   **Background information**  Mechanism: 5-Fluorouracil and its prodrug capecitabine are mainly converted by dihydropyrimidine dehydrogenase (DPD) to inactive metabolites. Genetic variations result in reduced DPD activity and thereby to reduced conversion of 5-fluorouracil to inactive metabolites. As a result, the intracellular concentration of the active metabolite of 5-fluorouracil can increase, resulting in severe, potentially fatal toxicity. For more information about the phenotype gene activity score 1.5: see the general background information about DPD on the KNMP Knowledge Bank or on www.knmp.nl (search for DPD).   Clinical consequences: 4 of the 6 studies and two meta-analyses found an increased risk of grade ≥ 3 toxicity. One study involving 19 c.2846A>T carriers found no significantly increased risk of grade ≥ 3 toxicity for c.2846A>T, but did find an increased risk for c.2846A>T and *13 combined. This study found no increased risk of grade ≥ 3 toxicity for 58 c.1236G>A carriers, but did find a trend towards an increased risk of hospitalisation. One study found an increased risk of grade ≥ 2 toxicity. Increased grade ≥ 3 toxicity: OR = 2.2-9.35 and RR = 3.0; RR = 1.59 for gene activity score 1.5 plus PHENO (*1/c.1236G>A+c.1236G>A/c.1236G>A). The percentage of patients with grade ≥ 3 toxicity was 109-1175% higher. One *1/c.2846A>T died due to toxicity after two cycles of a standard dose. One patient with genotype *1/c.1236G>A, who started on the standard dose, developed grade 3-4 toxicity during the first cycle.  No association with grade ≥ 3 toxicity was found in one small study of 21 patients with grade ≥ 3 toxicity. 1 patient (*1/c.496A>G) died as a result of the toxicity.  When the dose for 8 *1/c.2846A>T was guided by toxicity, the average dose in the sixth cycle was 76% of the standard dose. 5 patients with genotype *1/c.1236G>A did not develop grade ≥ 3 toxicity at 75 % of the standard dose. The two patients for who the dose was then increased tolerated the standard dose. One study found a tolerated dose of 78% of the standard dose in 6 *1/c.1236G>A and 85% of the standard dose in 1 *1/c.2846A>T (after starting at 75% of the standard dose). In one study in which intolerance/toxicity was induced by dose increase or use of the standard dose from the start, in 6% of the *1/c.1236G>A and 12% of the *1/c.2846A>T, 51 *1/c.1236G>A and 17 *1/c.2846A>T had a higher incidence of grade ≥ 3 toxicity at a scheduled 75% of the standard initial dose than patients without a gene variation at the standard dose. The titrated dose for *1/c.1236G>A in this study was 74% of the standard dose and the DPD activity was an average 80% (standard deviation 30%) and median 74% of the value for patients without a gene variation. The titrated dose for *1/c.2846A>T in this study was 64% of the standard dose and the DPD activity was 66% (standard deviation 20%) and median 67% of the value for patients without a gene variation. There was no difference in grade ≥ 3 toxicity between 11 patients with a gene variation at a reduced dose – including 6 *1/c.1236G>A and 1 *1/c.2846A>T at 75% of the standard initial dose – and patients without a gene variation on the standard initial dose. There was no difference in grade ≥ 3 toxicity between 22 patients with a gene variation at a reduced dose – including 11 *1/c.1236G>A on 50-75% of the standard initial dose and 1 *1/c.2846A>T at 60% of the standard initial dose – and patients without a gene variation on the standard initial dose.    Kinetic consequences:  40-58% decrease in clearance.  **Literature**   1. Kleinjan JP et al. Tolerance-based capecitabine dose escalation after DPYD genotype-guided dosing in heterozygote DPYD variant carriers: a single-center observational study. Anticancer Drugs 2019 Jan 8 [Epub ahead of print]. 2. Lunenburg CATC et al. Diagnostic and therapeutic strategies for fluoropyrimidine treatment of patients carrying multiple DPYD variants. Genes (Basel) 2018;9:E585. 3. Lunenburg CATC et al. Standard fluoropyrimidine dosages in chemoradiation therapy result in an increased risk of severe toxicity in DPYD variant allele carriers. Eur J Cancer 2018;104:210-8. 4. Henricks LM et al. DPYD genotype-guided dose individualisation of fluoropyrimidine therapy in patients with cancer: a prospective safety analysis. Lancet Oncol 2018;19:1459-67 en persoonlijke communicatie (getitreerde dosis en mediane DPD-activiteit). 5. Madi A et al. Pharmacogenetic analyses of 2183 patients with advanced colorectal cancer; potential role for common dihydropyrimidine dehydrogenase variants in toxicity to chemotherapy. Eur J Cancer 2018;102:31-9. 6. Meulendijks D et al. Pretreatment serum uracil concentration as a predictor of severe and fatal fluoropyrimidine-associated toxicity. Br J Cancer 2017;116:1415-24. 7. Lunenburg CA et al. Evaluation of clinical implementation of prospective DPYD genotyping in 5-fluorouracil- or capecitabine-treated patients. Pharmacogenomics 2016;17:721-9. 8. Lee AM et al. Association between DPYD c.1129-5923 C>G/hapB3 and severe toxicity to 5-fluorouracil-based chemotherapy in stage III colon cancer patients: NCCTG N0147 (Alliance). Pharmacogenet Genomics 2016;26:133-7. 9. Meulendijks D et al. Clinical relevance of DPYD variants c.1679T>G, c.1236G>A/HapB3, and c.1601G>A as predictors of severe fluoropyrimidine-associated toxicity: a systematic review and meta-analysis of individual patient data. Lancet Oncol 2015;16:1639-50. 10. Lee AM et al. DPYD variants as predictors of 5-fluorouracil toxicity in adjuvant colon cancer treatment (NCCTG N0147). J Natl Cancer Inst 2014;106:dju298. 11. Rosmarin D et al. Genetic markers of toxicity from capecitabine and other fluorouracil-based regimens: investigation in the QUASAR2 study, systematic review, and meta-analysis. J Clin Oncol 2014; 32:1031-9. 12. Terrazzino S et al. DPYD IVS14+1 G>A and 2846A>T genotyping for the prediction of severe fluoropyrimidine-related toxicity: a meta-analysis. Pharmacogenomics2013; 14:1255-72. 13. Deenen MJ et al. Relationship between single nucleotide polymorphisms and haplotypes in DPYD and toxicity and efficacy of capecitabine in advanced colorectal cancer. Clin Cancer Res 2011; 17:3455-68. 14. Kristensen MH et al. Variants in the dihydropyrimidine dehydrogenase, methylenetetrahydrofolate reductase and thymidylate synthase genes predict early toxicity of 5-fluorouracil in colorectal cancer patients. J Int Med Res 2010; 38:870-83. 15. Gross E et al. Strong association of a common dihydropyrimidine dehydrogenase gene polymorphism with fluoropyrimidine-related toxicity in cancer patients. PLoS ONE 2008;3:e4003. 16. Capitain O et al. The influence of fluorouracil outcome parameters on tolerance and efficacy in patients with advanced colorectal cancer. Pharmacogenomics J 2008;8:256-67. 17. Boisdron-Celle M et al. 5-Fluorouracil-related severe toxicity: a comparison of different methods for the pretherapeutic detection of dihydropyrimidine dehydrogenase deficiency. Cancer Lett 2007;249:271-82. 18. Cho HJ et al. Thymidylate synthase (TYMS) and dihydropyrimidine dehydrogenase (DPYD) polymorphisms in the Korean population for prediction of 5-fluorouracil-associated toxicity. Ther Drug Monit 2007;29:190-6. 19. Morel A et al. Clinical relevance of different dihydropyrimidine dehydrogenase gene single nucleotide polymorphisms on 5-fluorouracil tolerance. Mol Cancer Ther 2006;5:2895-904. 20. Yamaguchi K et al. Germline mutation of dihydropyrimidine dehydrogenese gene among a Japanese population in relation to toxicity to 5-fluorouracil. Jpn J Cancer Res 2001;92:337-42. 21. van Kuilenburg AB et al. Clinical implications of dihydropyrimidine dehydrogenase (DPD) deficiency in patients with severe 5-fluorouracil-associated toxicity: identification of new mutations in the DPD gene. Clin Cancer Res 2000;6:4705-12. 22. SPC’s Fluorouracil PCH, Xeloda, Efudix crème, Fluorouracil (VS) en Xeloda (VS). |
| --- |
