# Supplementary material for: Dutch Pharmacogenetics Working Group (DPWG) guideline for the gene–drug interaction of DPYD and fluoropyrimidines
Source: Eur J Hum Genet. 2019 Nov 19;28(4):508–17. doi: 10.1038/s41431-019-0540-0 (PMC7080718; doi:10.1038/s41431-019-0540-0)
Supplement: Supplementary file 8 — Suggested clinical decision support texts for health care professionals for tegafur with DPD inhibitors [file 41431_2019_540_MOESM8_ESM.docx]

**Supplementary Table 8:** Suggested clinical decision support texts for health care professionals for tegafur with DPD inhibitors

| **Gene activity score 0: tegafur**  **Pharmacist text / Hospital text / Prescriber text**  The gene variation increases the risk of severe, possibly fatal toxicity. A reduced conversion of tegafur to inactive metabolites means that the normal dose is an overdose.   - Avoid tegafur Fluorouracil and capecitabine are not suitable alternatives, as these are also metabolised by DPD.   If it is not possible to avoid tegafur: start with a very low dose and adjust the initial dose based on toxicity and efficacy. A substantiated recommendation for dose reduction cannot be made based on the literature. The recommendation for fluorouracil and capecitabine is to determine the residual DPD activity in mononuclear cells from peripheral blood and to adjust the initial dose accordingly. A patient with 0.5% of the normal DPD activity tolerated 0.8% of the standard capecitabine dose (150 mg every 5 days). A patient with undetectable DPD activity tolerated 0.43% of the standard capecitabine dose (150 mg every 5 days with every third dose skipped)  **Background information**  Mechanism: Tegafur is mainly converted by CYP2A6 to 5-fluorouracil. 5-Fluorouracil is mainly (> 80 %) converted by dihydropyrimidine dehydrogenase (DPD) to inactive metabolites. Genetic variations result in reduced DPD activity and thereby to reduced conversion of 5-fluorouracil to inactive metabolites. As a result, the intracellular concentration of the active metabolite of 5-fluorouracil can increase, resulting in severe, potentially fatal toxicity. Tegafur is used in combination with the DPD inhibitor gimeracil (molar ratio 1:0.4) and was used in combination with the DPD inhibitor uracil (molar ratio 1:4). Both DPD inhibitors exhibit competitive inhibition of DPD. This is why efficacy is achieved at lower concentrations of the metabolites formed by DPD, which seem to contribute to the toxicity. Inhibition by DPD inhibitors is reversible and reduces over time. For more information about the phenotype gene activity score 0: see the general background information about DPD on the KNMP Knowledge Bank or on www.knmp.nl (search for “DPD”).   Clinical consequences: There are no studies into the clinical consequences of tegafur in combination with a DPD inhibitor for gene activity score 0. The SmPC states that this combination is contra-indicated in patients with DPD deficiency. This probably refers to gene activity score 0. No safe dose has been found for gene activity score 0 for 5-fluorouracil (the metabolite of tegafur). In addition to this, four patients with a less strongly reduced DPD activity (gene activity score 1 or 1.5) had a comparable toxicity for treatment with tegafur/uracil as found for treatment with 5-fluorouracil or capecitabine.   Kinetic consequences: There are no studies into the kinetic consequences.  **Literature**   1. Deenen MJ et al. Standard-dose tegafur combined with uracil is not safe treatment after severe toxicity from 5-fluorouracil or capecitabine. Ann Intern Med 2010;153:767-8. 2. SPC Teysuno. |
| --- |

| **PHENO: tegafur**  **Pharmacist text / Hospital text / Prescriber text**  The gene variation increases the risk of severe, possibly fatal toxicity. A reduced conversion of tegafur to inactive metabolites means that the normal dose is an overdose.   - Avoid tegafur or start with a low dose and adjust the initial dose based on toxicity and efficacy   Fluorouracil and capecitabine are not alternatives, as these are also metabolised by DPD.  It is not possible to offer substantiated advice for dose reduction based on the literature. For fluorouracil and capecitabine, it is recommended to determine the residual DPD activity in mononuclear cells from peripheral blood and adjust the initial dose based on phenotype and genotype.  **Background information**  Mechanism: Tegafur is mainly converted by CYP2A6 to 5-fluorouracil. 5-Fluorouracil is mainly (> 80 %) converted by dihydropyrimidine dehydrogenase (DPD) to inactive metabolites. Genetic variations result in reduced DPD activity and thereby to reduced conversion of 5-fluorouracil to inactive metabolites. As a result, the intracellular concentration of the active metabolite of 5-fluorouracil can increase, resulting in severe, potentially fatal toxicity. Tegafur is used in combination with the DPD inhibitor gimeracil (molar ratio 1:0.4) and was used in combination with the DPD inhibitor uracil (molar ratio 1:4). Both DPD inhibitors exhibit competitive inhibition of DPD. This is why efficacy is achieved at lower concentrations of the metabolites formed by DPD, which seem to contribute to the toxicity. Inhibition by DPD inhibitors is reversible and reduces over time. For more information about the phenotype ”phenotyping”: see the general background information about DPD on the KNMP Knowledge Bank or on www.knmp.nl (search for “DPD”).   Clinical consequences: There are no studies into the clinical consequences of tegafur in combination with a DPD inhibitor for PHENO. However, four patients with a less strongly reduced DPD activity (gene activity score 1 or 1.5) had a comparable toxicity for treatment with tegafur/uracil as found for treatment with fluorouracil or capecitabine. In addition to this, four patients with gene activity score 1 could be treated with 90 % of the standard tegafur/uracil dose without grade 3-4 toxicity occurring.  Kinetic consequences: There are no studies into the kinetic consequences.  **Literature**   1. Cubero DI et al. Tegafur-uracil is a safe alternative for the treatment of colorectal cancer in patients with partial dihydropyrimidine dehydrogenase deficiency: a proof of principle. Ther Adv Med Oncol 2012;4:167-72. 2. Deenen MJ et al. Standard-dose tegafur combined with uracil is not safe treatment after severe toxicity from 5-fluorouracil or capecitabine. Ann Intern Med 2010;153:767-8. 3. SPC Teysuno. |
| --- |

| **Gene activity score 1.0: tegafur**  **Pharmacist text / Hospital text / Prescriber text**  The gene variation increases the risk of severe, possibly fatal toxicity. A reduced conversion of tegafur into inactive metabolites means that the normal dose is an overdose.   - Avoid tegafur or start with a low dose and adjust the initial dose based on toxicity and efficacy Fluorouracil and capecitabine are not alternatives, as these are also metabolised by DPD. It is not possible to offer substantiated advice for dose reduction based on the literature. For fluorouracil and capecitabine, starting with 50 % of the standard dose is recommended.   **Background information**  Mechanism: Tegafur is mainly converted by CYP2A6 to 5-fluorouracil. 5-Fluorouracil is mainly (> 80 %) converted by dihydropyrimidine dehydrogenase (DPD) to inactive metabolites. Genetic variations result in reduced DPD activity and thereby to reduced conversion of 5-fluorouracil to inactive metabolites. As a result, the intracellular concentration of the active metabolite of 5-fluorouracil can increase, resulting in severe, potentially fatal toxicity. Tegafur is used in combination with the DPD inhibitor gimeracil (molar ratio 1:0.4) and was used in combination with the DPD inhibitor uracil (molar ratio 1:4). Both DPD inhibitors exhibit competitive inhibition of DPD. This is why efficacy is achieved at lower concentrations of the metabolites formed by DPD, which seem to contribute to the toxicity. Inhibition by DPD inhibitors is reversible and reduces over time. For more information about the phenotype gene activity score 1: see the general background information about DPD on the KNMP Knowledge Bank or on www.knmp.nl (search for “DPD”).   Clinical consequences: In a study, two patients had a comparable toxicity for treatment with tegafur/uracil as found for treatment with 5-fluorouracil or capecitabine. In another study, four patients could be treated with 90 % of the standard tegafur/uracil dose without grade 3-4 toxicity occurring. All six patients had the genotype *1/*2A.   Kinetic consequences: There are no studies into the kinetic consequences.  **Literature**   1. Cubero DI et al. Tegafur-uracil is a safe alternative for the treatment of colorectal cancer in patients with partial dihydropyrimidine dehydrogenase deficiency: a proof of principle. Ther Adv Med Oncol 2012;4:167-72. 2. Deenen MJ et al. Standard-dose tegafur combined with uracil is not safe treatment after severe toxicity from 5-fluorouracil or capecitabine. Ann Intern Med 2010;153:767-8. 3. SPC Teysuno. |
| --- |

| **Gene activity score 1.5: tegafur**  **Pharmacist text / Hospital text / Prescriber text**  The gene variation increases the risk of severe, possibly fatal toxicity. A reduced conversion of tegafur into inactive metabolites means that the normal dose is an overdose.   - Avoid tegafur or start with a low dose and adjust the initial dose based on toxicity and efficacy Fluorouracil and capecitabine are not alternatives, as these are also metabolised by DPD. It is not possible to offer substantiated advice for dose reduction based on the literature. For fluorouracil and capecitabine, starting with 50 % of the standard dose is recommended and the dose should then be adjusted based on toxicity and effectiveness. In one study, the average dose of fluorouracil/capecitabine after titration was 64% of the standard dose for 17 patients with genotype *1/c.2846A>T and 74% of the standard dose for 51 patients with genotype *1/c.1236G>A.     **Background information**  Mechanism: Tegafur is mainly converted by CYP2A6 to 5-fluorouracil. 5-Fluorouracil is mainly (> 80 %) converted by dihydropyrimidine dehydrogenase (DPD) to inactive metabolites. Genetic variations result in reduced DPD activity and thereby to reduced conversion of 5-fluorouracil to inactive metabolites. As a result, the intracellular concentration of the active metabolite of 5-fluorouracil can increase, resulting in severe, potentially fatal toxicity. Tegafur is used in combination with the DPD inhibitor gimeracil (molar ratio 1:0.4) and was used in combination with the DPD inhibitor uracil (molar ratio 1:4). Both DPD inhibitors exhibit competitive inhibition of DPD. This is why efficacy is achieved at lower concentrations of the metabolites formed by DPD, which seem to contribute to the toxicity. Inhibition by DPD inhibitors is reversible and reduces over time. For more information about the phenotype gene activity score 1.5: see the general background information about DPD on the KNMP Knowledge Bank or on www.knmp.nl (search for “DPD”).   Clinical consequences: Two patients with gene activity score 1.5 (1x *1/c.1236G>A and 1x *1/c.2846A>T) had a comparable toxicity for treatment with tegafur/uracil as found for treatment with fluorouracil or capecitabine. Four patients with gene activity score 1 could be treated with 90 % of the standard tegafur/uracil dose without grade 3-4 toxicity occurring.  Kinetic consequences: There are no studies into the kinetic consequences.  **Literature**   1. Cubero DI et al. Tegafur-uracil is a safe alternative for the treatment of colorectal cancer in patients with partial dihydropyrimidine dehydrogenase deficiency: a proof of principle. Ther Adv Med Oncol 2012;4:167-72. 2. Deenen MJ et al. Standard-dose tegafur combined with uracil is not safe treatment after severe toxicity from 5-fluorouracil or capecitabine. Ann Intern Med 2010;153:767-8. 3. SPC Teysuno. |
| --- |
