# Supplementary material for: Dutch Pharmacogenetics Working Group (DPWG) guideline for the gene–drug interaction of DPYD and fluoropyrimidines
Source: Eur J Hum Genet. 2019 Nov 19;28(4):508–17. doi: 10.1038/s41431-019-0540-0 (PMC7080718; doi:10.1038/s41431-019-0540-0)
Supplement: Supplementary file 9 — The clinical implication score of DPYD-fluoropyrimidines is “essential”, based on the criteria and corresponding scores given by the DPWG [file 41431_2019_540_MOESM9_ESM.docx]

**Supplementary Table 9:** The clinical implication score of *DPYD*-fluoropyrimidines is “essential”, based on the criteria and corresponding scores given by the DPWG

| **Clinical Implication Score Criteria** | **Possible Score** | **Given**  **Score** |
| --- | --- | --- |
| **Clinical effect associated with gene/drug interaction**  •       3 (D) ≤ CTCAE Grade ≤ 4 (E)  •  4 (E) < CTCAE Grade ≤ 5 (F)  •       Increased efficacy | +  ++  + | ++^1^ |
| **Level of evidence supporting the associated clinical effect**  •       One study with level of evidence score 3  •       At least two studies with level of evidence score 3  •       Three or more studies with level of evidence score 3 | +  ++ +++ | +++^2^ |
| **Effectiveness of the intervention**  Number needed to genotype (NNG)  • 100 < NNG ≤ 1000  • 10 < NNG ≤ 100  • NNG ≤ 10 | +  ++  +++ | ++^3^ |
| **PGx information in the drug-label**  • Recommendation to genotype  • At least one genotype/phenotype mentioned as a contraindication | +  + | +^4^ |
| **Total Score:** | 9+ | 8+ |
| **Corresponding Clinical Implication Score*:** | | **Essential** |

^1.^Patients assigned to be DPD deficient but have received normal doses of fluoropyrimidines been associated with CTCAE Grade 5 toxicity.

^2.^Eight studies of sufficient quality have shown an association with CTCAE Grade 5 toxicity (references in Supplementary Table 1: 10, 15, 16, 18, 26, 29, 30 and 33).

^3.^The NNG was calculated using the “Calculations of the number of adverse events prevented with an effective preemptive genotyping program” (1). The pooled odds ratios and relative risks for*2A, 1236A, 2846T and *13 was 5.2, extracted from meta-analyses Meulendijks et al., Terrazzino et al., and Rosmarin et al. (2-4) The calculated NNG was 53.9.

^4.^ In the European Union, DPD deficiency is mentioned in the current version of the summary of product characteristics (SPC) of capecitabine in the sections Contraindications and Special Warnings and Precautions for Use (5). Similar information on DPD deficiency is provided in the United States by the Food and Drug Administration (FDA) for capecitabine (6). Comparable reports are made in SPCs of 5-FU (7, 8)

* essential, beneficial, potentially beneficial or not required.

References:

1. University V. Calculations of the number of adverse events prevented with an effective preemptive genotyping program vanderbilt.edu: Vanderbilt University; 2017 [Available from: <http://data.vanderbilt.edu/rapache/Case4PG> Accessed 20 November 2017

2. Meulendijks D, Henricks LM, Sonke GS, Deenen MJ, Froehlich TK, Amstutz U, et al. Clinical relevance of DPYD variants c.1679T>G, c.1236G>A/HapB3, and c.1601G>A as predictors of severe fluoropyrimidine-associated toxicity: a systematic review and meta-analysis of individual patient data. Lancet Oncol. 2015;16(16):1639-50.

3. Terrazzino S, Cargnin S, Del Re M, Danesi R, Canonico PL, Genazzani AA. DPYD IVS14+1G>A and 2846A>T genotyping for the prediction of severe fluoropyrimidine-related toxicity: a meta-analysis. Pharmacogenomics. 2013;14(11):1255-72.

4. Rosmarin D, Palles C, Pagnamenta A, Kaur K, Pita G, Martin M, et al. A candidate gene study of capecitabine-related toxicity in colorectal cancer identifies new toxicity variants at DPYD and a putative role for ENOSF1 rather than TYMS. Gut. 2015;64(1):111-20.

5. European Medicines Agency. [Capecitabine (Xeloda)] Summary of Product Characteristics.: UK: Roche Registration limited; 2008 [updated 26 July 2016. Available from: <www.ema.europa.eu>. Accessed 20 November 2017.

6. US FDA. Precribing information Xeloda [Available from: <http://www.fda.gov>. Accessed 20 November 2017.

7. Dutch Medicines Agency. SPC Fluorouracil Accord 50mg/ml [Available from: <http://www.cbg-meb.nl>. Accessed 20 November 2017.

8. US FDA. Prescribing information fluorouracil Teva Pharms USA [Available from: <http://www.fda.gov> Accessed 20 November 2017.
