# Supplementary material for: Dutch Pharmacogenetics Working Group (DPWG) guideline for the gene–drug interaction of DPYD and fluoropyrimidines
Source: Eur J Hum Genet. 2019 Nov 19;28(4):508–17. doi: 10.1038/s41431-019-0540-0 (PMC7080718; doi:10.1038/s41431-019-0540-0)
Supplement: Supplementary file 10 — Supplementary Material [file 41431_2019_540_MOESM10_ESM.docx]

**Supplementary Material 1:** Search terms used to perform the literature review of *DPYD*-[5-FU/capecitabine/tegafur] interactions

Search strategy:

Pubmed was used to search English, Dutch, German articles were accepted. Keywords were the drugs of interest (fluorouracil, capecitabine and tegafur/S1), the gene and variations (*DPYD*, DPD, dihydropyrimidine dehydrogenase), and others (e.g. metabolizer, pharmacogenetics, polymorphism).

The complete search string was:

Fluorouracil and capecitabine:

Search performed in 2009: ("Fluorouracil"[Mesh] OR fluorouracil) AND ("Dihydropyrimidine Dehydrogenase Deficiency"[Mesh] OR metabolizer OR metaboliser OR polymorph* OR "Polymorphism, Genetic"[MeSH] OR "Pharmacogenetics"[MeSH]) AND (English[lang] OR German[lang] OR Dutch[lang])

("capecitabine "[Substance Name] OR capecitabine) AND ("Dihydropyrimidine Dehydrogenase Deficiency"[Mesh] OR metabolizer OR metaboliser OR polymorph* OR "Polymorphism, Genetic"[MeSH] OR "Pharmacogenetics"[MeSH]) AND (English[lang] OR German[lang] OR Dutch[lang])

("Fluorouracil"[Mesh] OR fluorouracil OR "capecitabine "[Substance Name] OR capecitabine) AND (("Dihydrouracil Dehydrogenase (NADP)"[Mesh] OR (dihydropyrimidine dehydrogenase)) AND mutation) AND (English[lang] OR German[lang] OR Dutch[lang])

Search performed in 2014: ("Fluorouracil"[Mesh] OR fluorouracil OR "capecitabine" [Supplementary Concept] OR capecitabine) AND ("Dihydropyrimidine Dehydrogenase Deficiency"[Mesh] OR "Dihydropyrimidine Dehydrogenase Deficiency" OR metabolizer OR metaboliser OR polymorph* OR "Polymorphism, Genetic"[MeSH] OR "Pharmacogenetics"[MeSH]) AND (English[lang] OR German[lang] OR Dutch[lang])

Search performed in 2017: ("Fluorouracil"[Mesh] OR fluorouracil OR "Capecitabine"[Mesh] OR capecitabine OR fluoropyrimidines) AND ("Dihydrouracil Dehydrogenase (NADP)"[Mesh] OR  "Dihydropyrimidine Dehydrogenase Deficiency"[Mesh] OR "Dihydropyrimidine Dehydrogenase Deficiency" OR "Dihydropyrimidine Dehydrogenase" OR *DPYD* OR DPD) AND (English[lang] OR German[lang] OR Dutch[lang])

Search performed in 2019: ("Fluorouracil"[Mesh] OR fluorouracil OR "Capecitabine"[Mesh] OR capecitabine OR fluoropyrimidines) AND ("Dihydrouracil Dehydrogenase (NADP)"[Mesh] OR "Dihydropyrimidine Dehydrogenase Deficiency"[Mesh] OR "Dihydropyrimidine Dehydrogenase Deficiency" OR "Dihydropyrimidine Dehydrogenase" OR DPYD OR DPD) AND (English[lang] OR German[lang] OR Dutch[lang]) AND "2017/10/19 14.00"[MHDA]:"2019/01/30 16.20"[MHDA]

Tegafur:

Search performed in 2009 and 2012: ("Tegafur"[Mesh] OR tegafur[Text Word]) AND ("Dihydropyrimidine Dehydrogenase Deficiency"[Mesh] OR metabolizer OR metaboliser OR polymorph* OR "Polymorphism, Genetic"[MeSH] OR "Pharmacogenetics"[MeSH]) AND (English[lang] OR German[lang] OR Dutch[lang])

Search performed in 2015: ("Tegafur"[Mesh] OR "S 1 (combination)" [Supplementary Concept] OR  "tegafur-gimeracil-oteracil" [Supplementary Concept] OR tegafur[Text Word] OR S1 OR S-1 OR Teysuno) AND ("Dihydropyrimidine Dehydrogenase Deficiency"[Mesh] OR Dihydropyrimidine Dehydrogenase OR DPD OR *DPYD*) AND (English[lang] OR German[lang] OR Dutch[lang])

Search performed in 2017: ("Tegafur"[Mesh] OR "S 1 (combination)" [Supplementary Concept] OR  "tegafur-gimeracil-oteracil" [Supplementary Concept] OR tegafur OR S1 OR S-1 OR “S 1” OR Teysuno) AND ("Dihydrouracil Dehydrogenase (NADP)"[Mesh] OR  "Dihydropyrimidine Dehydrogenase Deficiency"[Mesh] OR "Dihydropyrimidine Dehydrogenase Deficiency" OR "Dihydropyrimidine Dehydrogenase" OR *DPYD* OR DPD) AND (English[lang] OR German[lang] OR Dutch[lang])

Search performed in 2019: ("Tegafur"[Mesh] OR "S 1 (combination)" [Supplementary Concept] OR  "tegafur-gimeracil-oteracil" [Supplementary Concept] OR tegafur OR S1 OR S-1 OR “S 1” OR Teysuno) AND ("Dihydrouracil Dehydrogenase (NADP)"[Mesh] OR  "Dihydropyrimidine Dehydrogenase Deficiency"[Mesh] OR "Dihydropyrimidine Dehydrogenase Deficiency" OR "Dihydropyrimidine Dehydrogenase" OR DPYD OR DPD) AND (English[lang] OR German[lang] OR Dutch[lang]) AND "2017/10/19 11.50"[MHDA]:"2019/02/12 16.45"[MHDA]
